# How Should the Worldwide Knowledge of Traditional Cancer Healing Be Integrated with Herbs and Mushrooms into Modern Molecular Pharmacology?

**DOI:** 10.3390/ph15070868

**Published:** 2022-07-14

**Authors:** Yulia Kirdeeva, Olga Fedorova, Alexandra Daks, Nikolai Barlev, Oleg Shuvalov

**Affiliations:** 1 Institute of Cytology, Russian Academy of Sciences, 194064 St. Petersburg, Russia; yulia.kirdeeva@yandex.ru (Y.K.); fedorovaolgand@gmail.com (O.F.); alexandra.daks@gmail.com (A.D.); 2 Orekhovich Institute of Biomedical Chemistry, 119435 Moscow, Russia

**Keywords:** traditional medicine, ethnomedicine, medical plants and mushrooms, cancer, anti-neoplastic compounds, medical herbs, pharmacology, standardization, bioavailability, safety

## Abstract

Traditional herbal medicine (THM) is a “core” from which modern medicine has evolved over time. Besides this, one third of people worldwide have no access to modern medicine and rely only on traditional medicine. To date, drugs of plant origin, or their derivates (paclitaxel, vinblastine, vincristine, vinorelbine, etoposide, camptothecin, topotecan, irinotecan, and omacetaxine), are very important in the therapy of malignancies and they are included in most chemotherapeutic regimes. To date, 391,000 plant and 14,000 mushroom species exist. Their medical and biochemical capabilities have not been studied in detail. In this review, we systematized the information about plants and mushrooms, as well as their active compounds with antitumor properties. Plants and mushrooms are divided based on the regions where they are used in ethnomedicine to treat malignancies. The majority of their active compounds with antineoplastic properties and mechanisms of action are described. Furthermore, on the basis of the available information, we divided them into two priority groups for research and for their potential of use in antitumor therapy. As there are many prerequisites and some examples how THM helps and strengthens modern medicine, finally, we discuss the positive points of THM and the management required to transform and integrate THM into the modern medicine practice.

## 1. Introduction

Cancer is the second greatest cause of mortality worldwide, accounting for nearly 10 million deaths in 2020 (World Health Organization, www.who.int/; accessed on 16 February 2022). Thus, this continuous challenge forces scientists to search for new antineoplastic drugs and approaches, and investigate their combinations, to better fight various types of malignancies.

Chemotherapy in combination with surgery is now the standard way to treat cancer. We analyzed the National Institutes of Health (NIH) list of cancer chemotherapeutic drugs (https://www.cancer.gov/about-cancer/treatment/drugs; accessed on 16 February 2022). Twenty-six of them (Table S1) are natural compounds derived from plants, actinomycetes, and marine organisms, or semi-synthetic derivates of these compounds. Despite the fact that this number does not look impressive, these compounds constitute the most frequently used drugs: doxorubicin, paclitaxel, docetaxel, etoposide, camptothecin, irino- and topotecan, vinblastine, vincristine, and vinorelbine. They are included in most chemotherapeutic regimes (Table S2) and have made a key impact on the chemotherapeutic cancer treatment. The chemical manipulation of these compounds continues to create new improved drugs.

However, our planet harbors a great biodiversity with about 391,000 plant species worldwide. These individual species produce tens of thousands of chemical compounds with a wide range of biological activities. Undoubtedly, dozens of them possess antineoplastic activity and may become important anticancer therapeutics. This assumption is confirmed through the examples of the biological compounds already mentioned above, which have been successfully applied to cure various types of malignancies.

On the other hand, up to 2 billion people (approximately one third of the population) have no access to modern medicines. For instance, in high-income countries, where comprehensive medical services are generally accessible, more than 80% of children with cancer are cured, opposed to less than 30% in low- and middle-income countries [1]. Under these circumstances of economic disparity, people from poor countries have no other choice but to rely on traditional medicine, which represents empirically collected evidence over many hundreds of years. Firstly, traditional medicine relies on the application of plants which are reservoirs of thousands of biologically active compounds. Thus, different cultures have adapted to use certain plants in their region to treat a spectrum of illnesses, including malignancies.

The use of traditional medicine is beneficial not only due to a lack of access to modern medicine, but also through sociocultural factors. The best examples are India, China, and Japan.

The Ayurveda medical system, which has roots that are millennia old, is based on a holistic (“whole-body”) healing system, which deals not only with the body but also with the mind and spirit [2,3]. A part of this system is associated with medical plants. Ayurvedic formulations are often complex and consist of several herbal-mineral ingredients, and are governed by well-described pharmacological principles of preparation, compatibility, and administration. With the support of the Government of India, a book in two parts—Ayurvedic Pharmacopoeia of India (API)—has been established. Part I (Volumes 1–6) of it contains information about natural substances (medical plants, minerals), whereas part II contains healing formulations which can be created from the constituents described in part I.

Ayurveda has been very popular in India for millennia and is of considerable interest all over the world. It applies dozens of plants with strong antineoplastic properties, which are now the focus of anticancer research [2,4].

Another example is traditional Chinese medicine (TCM). This is also a holistic body approach, which is aimed at restoring the body’s balance and harmony between the natural opposing forces of “yin” and “yang”, which can block the free circulation of internal ‘’qi’’ energy and cause disease. Traditional Chinese medicine includes acupuncture, diet, herbal therapy, meditation, physical exercise, and massages. The material part of TCM has partially evolved into Chinese proprietary medicine (CPM). This takes the form of a finished product, such as a capsule, tablet, or injection, all featuring the effective ingredients for use are documented in TCM [5]. CPM is a modern from of TCM which, due to standardization, can be used in modern medicine [6]. China’s government strongly supports this, exports CMP products to different countries for trials and therapy, and sets up research partnerships with the big international pharmaceutical companies [7,8].

Originally based on traditional Chinese medicine, Japan has created its own traditional medical system—Kampo—which has then evolved separately from TCM. Thus, Kampo is a uniquely Japanese form of medicine. It had been Japan’s primary health care system for over 1500 years. Despite the government approval of the Medical Care Law in 1874, which called for the adoption of the German model of health care and legitimized only western medical licenses, Japanese physicians continued to use and develop Kampo. Thus, 148 Kampo formulation extracts, 241 crude drugs, and 5 crude drug preparations are reported to be officially approved by National Health Insurance system, as well as under the Good Manufacturing Practice (GMP) Law, which was established by the government in 1987 to ensure that all Kampo products are of uniformly high quality [9]. Kampo is mainly based on plant extracts and formulations and is prescribed in line with modern drugs to treat various diseases including cancer and takes part in various clinical evaluations [10,11,12,13,14,15,16].

All of these traditional medical systems use herbs to a large extent. Despite these three examples, various other regions have their own medical traditions where herbs play most important roles (the traditional medicine of Maya, New Guinea, Philippines, etc.), which are not discussed in this paper but have been described in detail in several reviews [17,18,19].

Many of the herbs and formulations empirically defined over the centuries have also proven to be effective in preclinical and clinical investigations. They affect tumor cells both directly and through the modulation of the immune system, as well as through interrupts with cellular signaling pathways, miRNAs, and metabolic pathways [20,21,22], etc. We discuss here the antineoplastic properties of folk medicine plants and mushrooms; the molecular mechanisms of their bioactive constituents; and the advantages and limitations of using plants, mushrooms, and their active compounds in parallel with modern antineoplastic drugs.

We collected information from the common databases (MEDLINE/PubMed, Google Scholar, Web of Science, Scopus, Elsevier, SpringerLink, Wiley Online Library), as well as from several books and dissertations, and open databases.

Below, we summarized the information about some plants and mushrooms which have been applied by ethnomedicine to cure malignancies on five continents for a long time. We considered their antineoplastic properties and will focus on the molecular mechanisms of their activity. Finally, based on the data collected, we suggest two priority groups from the selected plants, mushrooms, and their bioactive compounds, for research and potential use in antineoplastic therapy.

## 2. Cancer Features Affected by Natural Drugs

**Figure 1:**
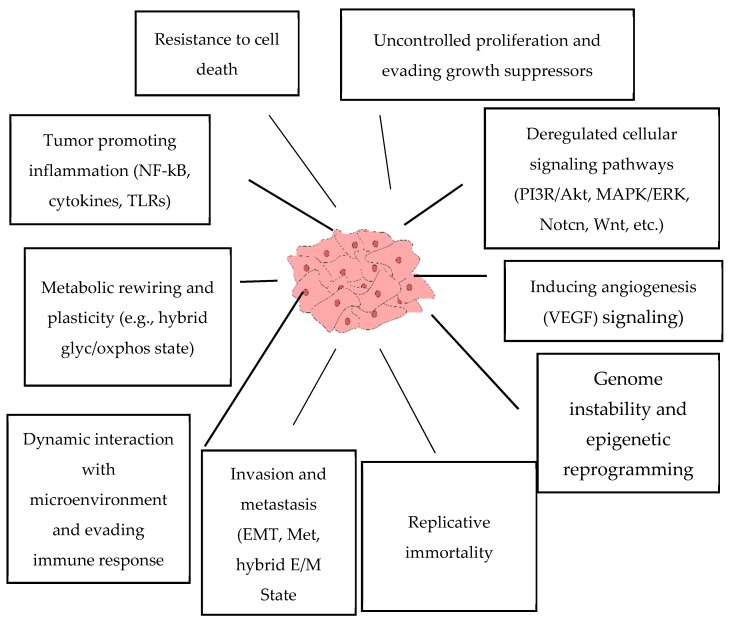
Main “hallmarks” of cancer.

There are common features of malignant cells which are well established (Figure 1) [23]. It is clear that both the genetic background, and somatic factors including cell-to-cell interactions, immunity, humoral factors, microenvironmental conditions, metabolic alterations, and others, are orchestrated during neoplasia. As a result, the altered balance in the equilibrium between oncogenes and tumor suppressors favors malignization. This disbalance results in uncontrolled cell division, resistance to apoptosis, metabolic rewiring, altered interactions with the microenvironment, as well as the acquisition of the ability to migrate and invade neighboring tissues, induce angiogenesis, evade the immune system, and become resistant to therapeutics, etc.

The genetic background is associated with “switch-on” mutations in gene coding for important oncogenes and “switch-off” mutations in tumor suppressors. Many known “switch-on” mutations of oncogenes, which are frequently observed in different neoplasia, lead to the constant activation of signaling pathways, including phosphoinositide 3-kinase/AKT serine/threonine kinase 1 (PI3K/AKT), Kirsten rat sarcoma virus/mitogen-activated protein kinases (Ras/MAPKs), Wnt family member 1, and others, which in turn drive and maintain cancer development. On the other hand, “switch-off” mutations in tumor suppressors, such as p53 (tumor protein p53), retinoblastoma (Rb), phosphatase and tensin homolog (PTEN), von Hippel–Lindau (VHL) tumor suppressor, and CDK4 inhibitor P16-INK4 (p16INK4), turn off their functions and mitigate the ability to combat malignization [24]. This results in the abnormalities within signaling pathway networks.

Certainly, all of the signaling pathways in our cells are involved in neoplasia development including PI3K/AKT, the extracellular signal-regulated kinase/mitogen-activated protein kinase (ERK/MAPK), Wnt, the Janus kinase/signal transducer and activator of transcription (JAK/STAT), transforming growth factor beta 1 (TGF-β), Hippo (mammalian Ste20-like 1 and 2) kinase—yes-associated protein 1 and transcriptional coactivator with PDZ-binding motif (Hippo—YAP/TAZ), Notch, and others [25]. One of the key roles in malignant cells is occupied by the PI3K/AKT and ERK/MAPK signaling pathways. Different biological stimuli and other signaling pathways converge on them. Thus, various growth factors, such as the epidermal growth factor (EGF), the fibroblast growth factor (FGF), the insulin-like growth factor (IGF), and the vascular endothelial growth factor (VEGF), bind to and activate their tyrosine kinase receptors which induce the signal transduction following the involvement of PI3K/AKT and ERK/MAPK signaling [26,27]. Thereby, the inhibition of these pathways is usually associated with the attenuation of tumor growth, migration, invasion, as well as the induction of apoptosis which makes them desirable drug targets.

AKT activates the master regulator of anabolism (mTOR) and deactivates AMP-activated protein kinase (AMPK) which is the inducer of autophagy. The inhibition of mTOR is one of the emerging successful strategies to kill malignant cells [28]. However, autophagy possesses a dual role in cancer. While it may contribute to cell death, it may also have cancer promoting properties including chemoresistance [29].

AMPK and mTOR are closely related to metabolic rewiring which is another “hallmark of cancer”. This supplies cancer cells with the materials needed for growth and provides an adaptational plasticity to changing conditions [30,31,32,33]. As an antineoplastic strategy, the use of metabolic inhibitors first started in the 1950s [34]. Now, their use during successful cancer therapies is once again a hot topic for discussion. The metabolic inhibitors which have been used clinically are methotrexate and its analogs, as well as gemcitabine, 5-fluouracil, lonidamine, AZD3965, telaglenastat, and others [32]. Moreover, new drugs have been designed, and preclinical and clinical studies are underway.

The balance between the expression of oncogenes and tumor suppressors is also regulated by epigenetics [35,36]. DNA and histone methyl transferases, histone deacetylases, and other chromatin-modifying participants are important targets for anti-cancer therapy [37,38,39].

As uncontrol growth and a resistance to programmed cell death are two major cancer cell features, the blocking of cell division and the induction of their death are the two key attributes of any chemotherapeutic methods. Several strategies can be implemented for this. The first relies on DNA-damaging agents, which stop the cell cycle and induce apoptosis (doxorubicin, cisplatin, etoposide, camptothecin, and others). Another way is based on mitotic poisons disrupting microtubule dynamics (paclitaxel, vinblastine, and vincristine). One more is the application of targeted therapy—drugs which specifically inhibit important modulators of the cell cycle and apoptosis. Some examples of them are tyrosine kinase inhibitors (TKIs—gefitinib, lapatinib, and sunitinib [40]), inhibitors of cyclin-dependent kinase (CDK—palbociclib, ribociclib, and abemaciclib [41]), inhibitors of antiapoptotic proteins B-cell lymphoma 2 (Bcl-2), and myeloid leukemia cell differentiation protein (Mcl-1—venetoclax and navitoclax) [42].

Upon cancer, the main reason of death is metastasis which disrupts the functions of organs. The initiation of metastazing is associated with the epithelial–mesenchymal transition (EMT). Epithelial cancer cells which underwent EMT become able to invade surrounding tissues and blood and lymph vessels, disseminate across the body, extravasate into new related niches, and establish secondary tumors. To create new tumor heaths, cancer cells undergo the reverse to a EMT process—mesenchymal–epithelial transition (MET) [43]. Today, it is suggested that malignant cells dwell in hybrid E/M state which allows them to switch between EMT and MET if required [43,44]. The targeting invasive and migration properties of tumor cells is very important for all chemotherapeutic regimes.

Cancer stem cells (CSCs) are a small subpopulation of cells within malignancy groups with capabilities of self-renewal, differentiation, and tumorigenicity when transplanted into an animal host [45]. Generally, they are resistant to chemotherapeutics, possess tumor-initiating and metastasis-initiating capacities, and are responsible for tumor recurrence and development [46,47].

Immunity plays a critical role in the clearance from neoplasia. Various molecular mechanisms make the anticancer response of immune cells ineffective, e.g., macrophages, as well as B- and T- lymphocytes. Thus, stimulating the activity of immune system by various mechanisms represents a very promising approach [48,49].

In this review, we aimed to focus on those plants and mushrooms, whose biological activity in cancer treatment has been proven, and discuss the mechanisms of their action using the knowledge of modern molecular medicine.

## 3. Plants from Different Continents Used in Ethnomedicine for the Treatment of Malignancies

Ethnobotanical and ethnomedicine studies point to medical plants with certain properties. Below, we summarized data on plants with anti-neoplastic capabilities which are implicated to heal cancer by indigenous people from five continents.

### 3.1. Africa

Due to its weak economic development, Africa is a continent with an elevated level of poverty. For most people, modern methods of cancer treatment are not available, such as chemotherapy, irradiation, and surgical resection [50,51].

The most famous example of African plant with anticancer properties is Madagascar periwinkle plant, *Catharanthus roseus* G. Don (Syn. *Vinca roseus* Linn), which is a source of vincristine and vinblastine. These compounds are often used to treat different malignancies worldwide.

Approximately 45,000 plant species grow in Africa [52] with the richest species diversity maximum in countries of West Sea coast from Gabon to Guinea, South African republic, and Western Africa (spanning Kenya, Tanzania, Uganda, Ethiopia, and South Sudan) [53].

According to a number of reviews, about a hundred of plants with anti-neoplastic properties are reported by ethnomedicine practitioners and are still used for a cancer treatment. However, for most of them, extremely limited information about their efficiency and selectivity in pre-clinical studies, active compounds, and molecular mechanisms of action is currently available.

***Acacia nilotica*** (the *Fabacea* family, “*Egyptian mimosa*”) which grows almost everywhere in Africa is widely used in traditional African medicine. It has been shown to possess antispasmodic, anti-inflammatory, antithrombotic, antioxidant, antidiarrheal, antibacterial, antihypertensive, and anticancer properties [54].

The seeds of this plant are used by people to treat breast, colon, head, and neck tumors [55]. Several in vitro and in vivo studies have demonstrated anticancer properties of alcohol and methanol extracts derived from *A. nilotica*. This plant turned out to be toxic predominantly to a breast cancer model (MCF7) than for normal liver cells [56]. Other researches have shown anticancer activity of this plant against glioblastoma [57], colon cancer [58], and other types of malignant cell models [55].

In vivo study has shown the significantly decreased development of solid and ascitic tumors induced by Dalton’s ascitic lymphoma in BALB/c mice [59], as well as *Helicobacter pylori*-induced colon tumors [60].

Foremost, the well-known chemicals quercetin, kaempferol, and ethyl-gallate are thought to be associated with antitumor and other activities of *A. nilotica* [54,61,62]. Pyrogallol was also shown to be the important anticancer chemical of *A. nilotica* which was able to strongly reduce colon tumors in mice models [58]. For all of these individual compounds, the significant anti-neoplastic properties have been demonstrated.

A detailed survey of *A. nilotica* traditional application, phytochemistry, and pharmacology is presented in review [54].

***Guiera senegalensis*** is a small shrub (the *Combretaceae* family) which grows in the savannah region of West and Central Africa. It is widely used in African traditional medicine to treat different ailments including malignancies [55,63].

A number of phenolic compounds which may mediate antitumor effects of *G. senegalis* were identified: gisorhamnetin, eupatorin, alpinumisoflavone, procyanidin B3, syringin, gallic acid, galloylquinic acid derivatives, quercetin, rhamnetin, kamferol, myricetin, (−)- epicatechin, and alkaloid guieranone A, etc. [63,64].

Plant-derived aqueous and methanolic extracts were cytotoxic against breast cancer [64]. The alkaloid guieranone A isolated from *G. senegalensis* demonstrated cytotoxic activity rather similar to doxorubicin against a panel of malignant cell models but not to normal hepatocytes [65]. The authors have also demonstrated the significant inhibition of angiogenesis. The study of silver nanoparticles derived from the leaves extract of *G. senegalensis* has shown a significant antiproliferative effect on human prostate (PC3), breast (MCF7), and liver (HepG2) cell models [66].

Thus, despite the antineoplastic activity of this plant, there are still not many studies devoted to this subject. However, the composition of chemicals with anti-cancer properties makes this plant perspective for cancer research.

***Combretum caffrum*** is the Eastern Cape South African bushwillow tree. The bark of this plant was shown to contain combretastatins—closely related stilbenes (combretastatins A), dihydrostilbenes (combretastatins B), phenanthrenes (combretastatins C), and macrocyclic lactones (combretastatins D).

Three common structural features of combtretastatins are: trimethoxy “A”-ring, a “B”-ring containing substituents o at C3’ and C4’, and (often) an ethene bridge between the two rings, which provides necessary structural rigidity and allows synthesis of different derivates [67].

The most promising and frequently tested compound in preclinical and clinical trials is water-soluble prodrug phospho-combretastatin A4 (CA-4P) which can be rapidly metabolized to combretastatin A4 (CA-4). This molecule exhibits anti-tumor properties by the attenuation of proliferation, and by targeting tumor vasculature paves [68]. It has a similar structure to colchicine, and binds tubulin at the same site. Moreover, CA-4 is effective against multidrug-resistant (MDR) cancer cells. A comprehensive overview of the structure, probable mechanisms of action, and potential applications is described in this review [68].

There are several detailed reviews systematizing the use of specific plants for the treatment of oncology in various regions of Africa: Western Africa [51,69], Central, Eastern, and North Africa [50], and South Africa [70,71,72,73].

There is no doubt that Africa, with its huge plant species diversity, is fraught with many currently unexplored plants and their biologically active compounds with strong antitumor properties. Some other African plants and their chemicals with anti-neoplastic activity are listed in Table 1.

According to a review by Alves-Silva and colleagues [28], the frequency with which different parts of the plant are used for cancer treatment: seeds (27%), hole aerial parts of plants (23%), leaves (22%), followed by roots (8%), fruits (7%), flowers (4%), bulbs (2%), cortex (2%), stamen (2%), rhizome (1%), hole plant mass (1%), and rinds (1%). For sure, the long-standing ethnical knowledge about the use of specific parts of a particular plant may reflect the distribution and amount of biologically active compounds among the plant. As stated by the same authors, the preparation methods for consumption are as follows: decoction (30%), grind with honey (24%), infusion (20%), brut (6%), extraction (4%), powder (4%), oil (3%), pomade (2%), ingestion (2%), cataplasm (1%), chewing (1%), washing (1%), mouth washing (1%), and inhalation (1%). Diverse types of preparation can be associated with the specific assimilation of biologically active compounds required across the body for treatment certain types of malignancies.

### 3.2. South America

South America is the territory of growth for about 82,000 plant species [83] which is approximately 1.6 times more than in Africa. However, the degree of study of their biochemical diversity and antitumor properties is similar to Africa.

***Tabebuia impetiginosa*** (“Lapacho”, the *Bignoniaceae* family) is a tree with rosy or purple flowers widely distributed among South and Central America. This is a very important medical tree which is used to treat inflammatory diseases, bacterial and viral infections, snake’s venom, and cancer [84]. In Brazil, *T. impetiginosa* is the most used plant to cure neoplasia. The stem bark and/or inner bark of this tree is utilized. It contains iridoid, lignan, isocoumarin, phenylethanoid, and phenolic glycosides [85]. Naphthoquinones lapachol and β-lapachone are the most attractive compounds from a medical point of view.

The application of both *T. impetiginosa* extracts and lapachone exhibits strong antiproliferative and cytotoxic activities [86,87,88,89] for human breast, colon, and hepatic cancer cell models. Lapachone was sold in Brazil by the Pernambuco Pharmaceutical Laboratory (LAFEPE) and used to cure malignancies [90].

It was shown that lapachol is a pyruvate kinase M2 (PKM2) inhibitor [91], thus quenching glycolysis and anabolic capacities. PKM2 is an enzyme which branches glucose flux into biosynthetic pathways [92,93]. β-lapachone inhibited lung metastasis in colorectal cancer models [87]. It selectively killed NADPH quinone oxidoreductase 1 (NQO1)-overexpressing hepatoma cells which were accompanied by ROS induction and PARP1 hyperactivation, causing a decrease in NAD+ and ATP levels, as well as a dramatic increase in DNA double-strand break lesions [94]. NQO1 is a prognostic marker in HCC; it was increased 18-fold in HCC versus normal livers, and its high level predicts poor outcome [95,96].

Active studies of lapachones started in the 1960s when these compounds were isolated from *T. impetiginosa*, but then were terminated due to their side effects. However, further experiments have shown that β-lapachone, α-lapachone, and some of their synthetic analogs are safe and are promising antineoplastic compounds (for a comprehensive review, see [89,97]).

As an example, Rone and colleagues have created lapachone-containing ruthenium (II) complexes which enhanced lapachone toxicity to cancer cells relative to normal cells over 100-fold. The cytotoxic effects were mediated by Aurora-B down-regulation and G2/M-phase cell cycle arrest [98]. The other group [99] developed long-circulating lapachone nanoparticles which remarkably prolonged its half-life in the body and increased brain intake in order to affect glioma cells.

A number of patents cover promising synthetic derivates of lapachones. Further chemical modifications are required to improve their safety and bioavailability. Recently, positive results were obtained in phase I/Ib of a multi-center clinical trial (NCT02514031) of β-lapachone with gemcitabine/nab-paclitaxel in patients with advanced pancreatic cancer [100]. However, further insights into the molecular mechanisms of lapachone anticancer activity are required.

Besides lapachone, furanonaphthoquinones from *T. impetiginosa* possess anticancer capabilities. They were the key structures required to hamper signal transducer and activator of transcription 3 (STAT3) phosphorylation which inhibits the JAK/STAT pathway [101].

Taken together, these data demonstrate the potential of *T. impetiginosa* and lapachones in cancer healing.

***Aloe vera* and *A. arborescence*** (the *Asphodelaceae* family) are stemless or very short-stemmed succulent plants of the genus Aloe. These species grow on several continents and are very frequently used to treat various diseases in Brazil including rheumatism, eczema, blood clots, diabetes, gastritis, inflammation, and malignancies.

*A. vera* and *A. arborescence* contain different biologically active secondary metabolites including anthraquinones, dihydroisocoumarins, naphthalenes, and polyketides [102]. Anthraquinones aloe-emodin, aloin A (barbaloin), and aloin B (isobarbaloin) are especially interesting for anticancer therapy. Extracts and individual compounds of Aloe induce cell cycle arrest [103,104] and apoptosis [105], exhibit antiangiogenic and antimetastatic properties [105,106], and decrease glucose flux and telomerase activity in a huge number of studies (including both solid and blood neoplasia) [107,108]. A comprehensive review of anticancer properties of *Aloe vera*, *A. arborescence*, and its active compounds is given in [108].

Aloe-emodin (Ae) exerts a plethora of important pharmacological properties including the anticancer ones (reviewed in [109]). The treatment of colorectal cancer cells with Ae induced ER stress and the activation of key components of the PERK pathway—glucose-related protein 78 (GRP78) and transcriptional factor C/EBP homologous protein (CHOP) up-regulation, protein kinase R (PKR)-like ER kinase (p-PERK), and eukaryotic initiation factor-2α (p-eIF2α) [110]. In NSCLCs, this compound activated MAPK signaling and inhibited Akt/mTOR pathway which led to an increase in ROS and autophagy [111].

Wang’s group have found that the AE compound is a competitive inhibitor of telomerase (hTERT) and a G-quadruplex structure stabilizer. In addition, Ae transcriptionally repressed tTERT via the up-regulation of E2F1 and the down-regulation of c-myc expressions [112]. G-quadruplexes are specific structures in DNA and RNA which are frequently observed in promotors of proliferation-related genes, chromosome ends, and telomeric regions, and are involved in transcription regulation. Due to the stability of G-quadruplexes and their presence within most human promoters of oncogenes, and at telomeres, G4 structures are promising targets and are currently being tested as a way to block the transcription of oncogenes and telomere elongation in cancer cells [113]. In line with this evidence, other groups have reported that Ae and Ae-8-glucoside are G4-binding ligands, especially for c-KIT and c-Myc oncogenes [114].

In melanoma cells, aloin down-regulates HMGB1 expression at the transcriptional level, preventing its translocation to the cytoplasm and interaction with TLR4, which indeed blocks HMGB1-mediated ERK activation [115]. In line with these data, in gastric cancer, the other group has shown an aloin-mediated inhibition of HMGB1 expression and release, as well as a HMGB1-induced activation of the Akt-mTOR-P70S6K and ERK-P90RSK-CREB signaling pathways [116].

Finally, aloin was shown to mitigate doxorubicin-induced cardiotoxicity by reducing proinflammatory cytokines—TNF-α, IL-1β, and IL-6 (Birari 2020) [117].

The polysaccharide acemannan exerts antitumor activity through the stimulation of the immune system and the production of antitumor cytokines, and has been approved by the U.S. Department of Agriculture (USDA) for treatment of fibrosarcoma in cats and dogs (Acemanna, CarraVet Acemannan immunostimulant) [108].

Although there have been numerous in vitro and in vivo studies, the antineoplastic potential of *Aloe* ssp. has not been fully studied. However, several clinical trials have been conducted. The combined adjuvant chemotherapy which includes Aloe arborescence, oxaliplatin, and 5-fluorouracil (5-FU), given to 240 patients with metastatic solid tumors, significantly improved tumor regressions and 3-year survival rates [118]. Two other trials have also indicated the potential of Aloe for anticancer therapy [119,120].

Despite the strong anticancer properties of Aloe, caution and further research is needed before its intake. Several studies have described the potential carcinogenic effects of Ae and aloin. Thus, Ae reportedly may have hepato- and nephrotoxicity [109] whereas aloin is able to induce the Wnt/β-catenin pathway [121].

***Capsicum frutenese*** is a member of the *Solanaceae* family which is frequently used in South American ethnomedicine to treat cancer. Other pepper species, including *C. chinensies* (*Chili pepper*), are also used. The spicy taste of these plants is caused mainly by alkaloid capsaicin.

A huge number of studies have demonstrated the capsaicin-mediated anticancer effects [122,123]. In non-small cell lung cancer (NSCLC), capsaicin inhibits vascular endothelial growth factor (VEGF) expression and angiogenesis via the p53-HIF1-VEGF pathway [124]. It was also shown that capsaicin, in combination with sorafenib, inhibited epidermal growth factor receptor (EGFR) and PI3K/Akt/mTOR signaling [125]. This synergic effect attenuated the growth, migration, and invasion, and also induced apoptosis, in three hepatocellular carcinoma cell lines. In nasopharyngeal carcinoma, capsaicin extinguished the PI3K/Akt/mTOR pathway which induced autophagy and apoptosis [126].

It is interesting to note that Chang and colleagues [127] have shown that Ecto-NADPH oxidase disulfide thiol exchanger 2 (ENOX2) is a direct target of capsaicin. Authors have shown that capsaicin induces autophagy-related apoptosis in p53-mutant oral carcinoma cells, but only autophagy-dependent cytotoxicity (without apoptosis) in cells with wild-type p53.

However, several contrary results have also been reported, implicating capsaicin’s pro-cancer properties [128]. For instance, high doses of capsaicin activated AMPK, and also induced autophagy, EMT, and chemoresistance [129]. These contradictions may depend on various factors including both the background of the cells and experimental conditions. While different studies report autophagy as a mechanism of capsaicin-mediated effects [129], the opposite results can also be linked to this fundamental process. It is already known that autophagy has a dual role in cancer, creating both pro-survival and antineoplastic effects [29]. Autophagy is typically associated with apoptosis. However, in other cases, it protects cancer cells from chemotherapy [130]. Arguably, the exact effects of capsaicin may depend on whether autophagy plays a pro- or anti-survival role in corresponding malignant cells.

Taken together, there is a possibility that capsaicin is a potential anticancer therapeutic; however, due to contradictory results, more detailed studies about its properties are required.

Some other South American plants and their compounds with anti-neoplastic activity are listed in Table 2.

### 3.3. Asia

Asia occupies a vast territory with various climate zones which range from tropical to arctic. It is a habitat for 100,000 plant species, many of which have been medically used in ethnomedicine for centuries.

***Cephalotaxus harringtonia*** (Japanese plum yew, the *Cephalotaxaceae* family) is an evergreen tree up which can grow up to 10 m tall and is native to Japan. Initially, the ethanolic extract from the seed of *Cephalotaxus harringtonia* showed antineoplastic activity against mouse leukemia L-1210 and P388 cells. Several alkaloids with potential antitumor activity were isolated from this extract and from other parts of the plant [142]. They are identified as cephalotaxin esters: harringtonine, isoharringtonine, homoharringtonine (HHT), and doxyharringtonine.

Clinical trials of HHT have been actively conducted in China and the USA in acute myeloid leukemia (AML) and chronic lymphocytic leukemia (CLL) [143]. The initial data obtained showed conflicting results; thus, interest among American scientists towards HHT has significantly weakened, unlike their Chinese colleagues.

Meanwhile, Chinese scientists continued clinical trials with varying regimes and HHT dosing. They carried out detailed studies and then successfully used HHT in a HAG combination scheme (homoharringtonine, cytarabine, and G-CSF) to treat hematological malignancies, including AML and myelodysplastic syndrome [144,145]. Thus, HHT became a part of the standard AML therapy in China [143]. In 2012, the Food and Drug Administration (FDA) approved omacetaxine—a semisynthetic purified HHT derivate for the treatment of patients with chronic myelogenous leukemia (CML) refractory or intolerance to two or more TKIs [146].

The mechanism of HHT and omacetaxine action is the inhibition of translation. These compounds compete with tRNA to bind the A-site cleft in the large ribosomal subunit which blocks elongation. Furthermore, another mechanism of HHT action in AML cells was discovered. It has been shown that HHT directly binds the NF-κB-repressing factor (NKRF) and arrests it in the cytoplasm, which in turn strengthens p65-NKRF interaction, thereby attenuating the transactivation activity of p65 on the MYC gene [147]. HHT was also shown to decrease p-JAK2, p-STAT5, and p-AKT, which suggests it may be a broad-spectrum PTK inhibitor [148]. Thus, multiple mechanisms of HHT activity may exist.

***Oldenlandia diffusa*** (*Hedyotis diffusa*) or “Snake-Needle Grass” and *O. corymbose* are the annual plants widely distributed in China, Japan, and Korea. In China, this plant is actively used in traditional medicine. *Oldenlandia diffusa* has analgetic, antibacterial, anti-inflammatory, antitumor, cardiotonic, diuretic, and sedative effects on the body. Regarding cancer, it is well known in Chinese folk medicine, primarily for the treatment of liver, lung, and stomach malignancies [149].

*O. diffusa* has been extensively used as a part of adjuvant therapy for metastatic breast cancer and gastric cancer patients in traditional Chinese medicine (TCM) with proven efficacy [150,151]. Regarding breast cancer studies, extracts of *O. diffusa* possessed cytotoxicity towards highly invasive breast cancer cells, but not towards normal cells of different origins. It abrogates the expression of metalloproteinases (MMPs) and caveolin-1 [152]. The extract inhibited p-ERK, p-38, NF-κB, MMP-9, and Icam-1 [153], and may also inhibit AMPK [154].

*Hedyotis diffusa* contains various iridoids (asperuloside, geniposidic acid, diffusoside, and alpigenoside), triterpenes (arborinone, ursolic acid, and oleanolic acid), flavonoinds (quercetin, rutin, and kaempferol), athraquinones, phenolic acids (p-coumaric acid, caffeic acid, and caffeoyl-quinic acids), and a broad spectrum of volatile oils (reviewed in [155]). Such a diverse composition of compounds with antineoplastic properties may explain the use of *O. diffusa* by Chinese people as an anticancer substance for centuries.

Feng and colleagues have demonstrated that *Hedyotis diffusa* extract attenuated the phosphorylation of AKT, ERK1/2, JNK, p38, ribosomal protein S6 kinase beta-1 (p70S6K), STAT3, and the secretion of pro-inflammatory interleukins IL-1β, IL-6, and TNF-α. Additionally, at the time, it also induced anti-inflammatory IL-4 and IL-10 [156].

A number of studies have shown that oleanic and ursolic acids fractioned from this plant are very important compounds due to their antitumor properties. The ursolic-acid-mediated inhibition of the RAF/ERK, IKK/NF-κB [157], and STAT3 pathways [158] is reported. It has also been shown that ursolic acid suppressed proliferation and induced apoptosis in breast cancer cells, but not in non-malignant cells. Ursolic acid also repressed metastasis in both zebrafish and mouse models via the suppression of glycolysis through the activation of SP1/caveolin-1 signaling [159]. Another research group has demonstrated that ursolic acid inhibited energy metabolism. It inhibited Akt which was also associated with decreased HK2, PKM2, ATP, and lactate levels [160]. The derivate of ursolic acid mimics glucose, and competes with it for hexokinase 2 (HK2) binding [161].

Oleanolic acid (OA), which is another bioactive component of *Hedyotis diffusa*, similarly attenuates cancer development through several mechanisms [162]. In gastric cancer, OA was shown to down-regulate glucose uptake and aerobic glycolysis through the inhibition of YAP and HIF-1α [163], and through the induction of autophagic death by deactivating PI3K/AKT/mTOR and ERK/p38 MAPK [164,165].

OA was shown to activate ferroptosis in Hela cells by promoting the expression of ACSL4 [166] (Xiaofei, et al., 2021) in the purine salvage pathway. It suppressed the purine salvage pathway (PSP), thus interfering with nucleotide synthesis. OA induced the autophagy-dependent degradation of hypoxanthine–guanine phosphoribosyltransferase (HGPRT) and 5’-nucleotidase (5’-NT), i.e., two enzymes of PSP [167]. The other group was able to show that OA may suppress angiogenesis in colorectal cancer by blocking VEGFR2 signaling [168].

For medical purposes in China, *Hedytois diffusa* is often used in tandem with another plant—*Scutellaria barbata*. This pare is a “core” of Chinese herbal medicine (CHM) which is utilized to treat different types of tumors [151,169].

***Scutellaria barbata*** (*SB*) is a perennial herb (the *Lamiaceae* family) living in southern central China. This medical plant is frequently used in TCM to cure malignancies, inflammation, infection, cirrhosis, etc. Among the chemical compounds identified, there are: flavonoids (scutellarein, scutellarin, carthamidin, isocarthamidin, wogonin, naringenin, apigenin, hispidulin, eriodictyol, and luteolin), diterpenoids (scutellones, scuterivulactones, barbatins, and scutebarbatines), and volatile oils (linalool, α-terpineol, thymol, and globulol) [155]. Flavonoids (scutellarein, scutellarin, and carthamidin) are thought to be the main compounds that are responsible for anticancer properties of SB.

BZL101 is an orally specified aqueous SB extract which has been extensively studied for the treatment of metastatic breast cancer. It provokes cell cycle arrest, apoptosis [170], inhibition of glycolysis, and OXPHOS [171].

Scutellarein inhibited the enhancer of zeste homolog 2 (EZH2), increased the expression of its target forkhead box protein O1 (FOXO1), and reduced tumor growth and metastasis [172]. Moreover, in HCC, scutellarein increased the level of PTEN—a negative regulator of Akt signaling pathway [173].

Another flavonoid compound—scutellarin—mitigates colitis-derived colorectal cancer by inhibiting the Wnt/β-catenin signaling pathway [174]. In gastric cancer cells, this compound up-regulates PTEN, which attenuates p-PI3K and EMT [167].

Extracts of this plant reduced p-STAT3, the expression of cyclin D1 and CDK4 [175], as well as the Wnt/β-catenin signaling pathway [176]. It may also attenuate the PI3K/AKT pathway, inhibit ABC transporters, and restore susceptibility to 5-FU [177].

Thus, the combination of *O. diffusa* and *S. barbata* extracts displays proven anti-neoplastic capacity and involves multiple mechanisms acting in a synergistic way. The study of a combination of extracts or individual compounds of these plants is a promising area of anticancer research.

The power of plants to fight cancer is exhaustively represented by traditional Chinese medicine (TCM) and Indian Ayurveda.

#### 3.3.1. Traditional China Herbal Medicine

The herbal part of TCM relies on the application of a cocktail consisting of several herbs, used in the treatment of complex diseases such as cancer. It has at least 2000 years of history. According to Chen and colleagues, Chinese Pharmacopoeia (2015 edition) counted 25 formulations with antineoplastic properties [178].

Traditional personal medicine (TPM) is the improved and more standardized kind of TCM application. TPM includes herbal medicines in traditional Chinese medicine, modernized into a ready-to-use form (such as tablets, oral solutions, or dry suspensions), as opposed to herbs that require cooking (hot water extraction).

The benefit of TCM formulas in the therapy of various neoplasms is based on multiple components, which can target multiple signaling pathways, providing synergistic therapeutic effects. Plants described in the earlier section are often the components of various TCM formulations. The analysis of a number of TCM formulas uncovered the mechanisms of their antitumor activities and enumerates their bioactive anticancer compounds [178,179,180,181].

Wu and colleagues have analyzed the application of the top 15 TPMs and modern western drugs according to the frequency of their use in a particular type of malignancy and the cost per patient [5]. This statistical analysis has shown that TPMs are used with about the same frequency as western therapeutics, whereas the cost per patient was lower for TPMs. It is interesting to note that different TPMs can be applied to treat certain types of malignancy with varying frequencies. Moreover, TPMs are often applicated in combination with western medicines [5]. The most frequently used antineoplastic formulations are given in Table 3 with brief descriptions.

#### 3.3.2. Ayurvedic Medicine

*Ayurveda*, translated from Sanskrit, meaning “life knowledge”, is an ancient Indian traditional medical system which has been practiced for more than 5000 years and is still applied now by many cultural tribes in Indian sub-continent. Ayurvedic medicine is a unique holistic approach where herbal medicines, special diets, yoga, relaxation methods, and lifestyle management are key strategies for curing various chronic diseases such as diabetes, cancer, cardiovascular, neurological disorders, and many others.

As reported by Kuruppu and colleagues, between 70 and 80% of people in India, Nepal, and Shri Lanka practice this medical system [197]. Ayurveda attracts attention in other regions and countries, including the USA and Europe, as an alternative medical way for health recovery and maintenance [198,199].

About 1700 medical substances of herbal, animal, and mineral origin give birth to 40,000 different formulations for internal consumption and hundreds for external application (Sujatha, et al., 2021).

Ayurveda is supported by the government of India through the *Ayurvedic Pharmacopoeia of India* (API). This is a unique book divided in two parts. Part I (volumes 1–6) contains information about medical plants and their substances, whereas part II contains formulations from compounds described in part I. All in all, 450 medical herbs are listed in this book.

Bhandari and colleagues reported about 10 formulations which are readily available in the Indian market to cure neoplasia [200]. Thus, Ayurveda accounts a few dozen plants with anticancer properties [2,197,201]. Some of them have been also used in TCM and elsewhere, so they were described earlier. Several other very important anticancer ayurvedic plants are discussed below.

***Withania somnifera*** (*WS*, “ashwagandha” or “winter cherry”, the *Solanaceae* family) is an annual evergreen shrub which grows in India, the Middle East, and in some African regions. This is a very important Ayurvedic plant which is used as an energy balancer, and to cure arthritis, anxiety, insomnia, bronchitis, male disfunctions, etc. Ashwagandha is also sold in western markets as a food supplement to increase energy and endurance [202].

The main biologically active chemical constituents of WS are alkaloids (isopelletierine, anaferine, cuseohygrine, anahygrine, etc.), steroidal lactones (withanolides and withaferins), and saponins [203]. The extracts of Ashwagandha selectively killed cancer cells and inhibited xenograft’s growth [204,205] through mitochondria-dependent apoptosis and G2/M cell cycle arrest. In other studies, extracts of *WS* suppressed the growth of malignant cell models and xenografts of breast, prostate, lung, gastrointestinal cancer, glioma, etc. This was associated with the down-regulation of p-AKT, VEGF, MMP-2, ERKp44/42 [206], cyclin D1, NF-kB, HSP-70, and NCAM, bcl-xl [207], as well as the reactivation of FOXO3a/Par4 [208]. The antineoplastic activity of WS is significantly associated with the presence of steroidal lactone whithaferin A.

A large number of studies have demonstrated the pleiotropic whithaferin-A-mediated down-regulation of cancer. This affects many characteristics of malignant cells (reviewed in [209,210]).

Withaferin A and withanone were able to attenuate EMT, driven by TNF-α and TGF-β in NSCLC cell lines H1299 and A549 [211]. Withaferin A inhibited glycolysis and complex III of the respiratory chain in breast cancer mouse models, indicating that it can interfere with metabolic rewiring in neoplasms [212]. A couple of studies reported that withaferin A can effectively target cancer stem cells (CSCs) [213,214].

Bearing in mind the safety and antitumor properties of *Withania somnifera* (Ashwagandha), its active constituent withaferin A should be studied in detail regarding therapeutical usage.

***Curcuma longa*** (“Tumeric”, the *Zingiberaceae* family) is a flowering plant, which is native to South Asia, India, and Indonesia. Its roots and rhizomes are widely used as a spice named “turmeric” which is a key ingredient in curry. This plant helps to reduce inflammation, hepatic and neurodegenerative disorders, metabolic syndrome, obesity, and other illnesses.

The major biologically active constituents of turmeric are diarylheptanoids, which occur in a mixture of dubbed curcuminoids (curcumin, desmethoxycurcumin, and bis-desmethoxycurcumin) that generally amount to approximately 1–6% of the plant by dry weight [215]. In addition, *C. longa* is another species of the Curcuma genus that contains a diverse composition of volatile (zingiberone, tumerone, and atlantone) compounds with a set of biological activities, including anticancer activity (reviewed in [216]).

However, the main pharmacological activity of *C. longa* is attributed to curcumin [217]. Curcumin acts through the modulation of multiple signaling pathways. It is known to inhibit the activity of transcriptional factors (STATs, Notch-1, NF-κB, PPAR-γ, WTG-1, and β-catechin), growth factors (FGF, VEGF, TGF-β1, TF, CTGF, and EGF), a number of receptors and kinases (EGFR, HER-2, CXCR4, MAPK, ERK1/2, RAK, PKA/B/C, Bcr-Abl, JNK, and IKK), and pro-survival proteins (Survivin, Mcl-1, Bcl-xL, cIAP-1, cIAP-2, and Bcl-2) [218,219].

Curcumin down-regulates cyclooxygenase (COX-2), EGFR, and ERK1/2 in lung and pancreatic cancer [220]. A number of literature sources report that curcumin activates autophagy in various malignancies, including melanoma, pancreatic [221] and gastric cancer [222], and glioma [223]. It may also target CSCs in esophageal carcinoma [224], hepatocellular carcinoma [225], and glioma [223]. Curcumin makes cancer cells more vulnerable to chemotherapeutic agents (doxorubicin, paclitaxel, 5-fluorouracil, and cisplatin) [226,227,228,229] and radiotherapy [230,231].

***Zingiber officinale*** is a widely known plant because its whole rhizome—ginger—is widely used as a spice and in folk medicine. Its healing effects extend to diseases of the gastrointestinal tract, as well as the broncho pulmonary system.

Ginger is rich in phenolic compounds including gingerols (6-gingerol, 8-gingerol, and 10-gingerol), shogaols, paradols, quercetin, zingerone, gingerenone-A, and 6-dehydrogingerdione. Moreover, ginger contains bioactive volatile oils. Its terpene compounds are zingiberene, β-bisabolene, α-curcumene, α-farnesene, and β-sesquiphellandrene [232,233].

There is a lot of evidence which shows the potential of ginger to prevent and suppress tumors, especially gastrointestinal cancer (GI). Ginger extracts and its individual constituents allow the multitargeted influence on cancer cells affecting Bcl2, p38/MAPK, EGFR, VEGF, AKT, ERK1/2, etc. [234].

In vivo studies have shown that ginger extract reduced NF-κB and TNF-α expression in rat livers with induced cancer [235]. Furthermore, 6-shogaol inhibits JAK2 and c-Src kinases [236], interleukin (IL)-6-induced STAT3, and TNF-α-induced NF-κB activation [237]. Zingerone and its derivates synergistically suppressed TGF-β-induced EMT and the invasion of hepatocellular carcinoma [238].

In addition, 6-shogaol reduced breast CSCs (CD44 + CD24−) and killed spheroids. This was associated with reduced Notch and its targets Hes1 and cyclin D1, and induced autophagy-based cell death [239].

In mice bearing Ehrlich carcinoma, the administration of doxorubicin in combination with ginger extract reduced the tumor volume and increased the survival rate by activating the AMPK pathway and reducing the cyclin D level [240]. In addition, both ginger extract and its isolated constituencies were shown to overcome methotrexate [241] and dodetaxel resistance [242] in AML and prostate cancer.

A more detailed description of ginger effects on the properties of various malignancies types is reviewed in [232,243,244].

***Boswellia serrata*** and other *Boswellia* species are very important ayurveda plants which have been used for centuries to treat chronic ailments—arthritis, inflammatory bowel disease, diabetes, asthma, cancer, and others.

This plant is the source of “Frankincense”, which is oleo gum resin extracted from the Boswellia species. Frankincense is a mixture of essential oils, polysaccharides, and resin acids. It contains a number of different types of boswellic acids (BAs) which are pentacyclic terpenoids. The main ones are: α- and β-BA, acetylated α- and β-BAs (ABA), 11-keto-β-BA (KBA), and 3-O-acetyl-11-keto-β-BA (AKBA) [245,246].

Essential oils are represented by α-thujene, α-terpineol, eudesmol, verbenene, thujone, pinocarveol, etc. [247,248]. Both BAs and volatile oils are responsible for Boswellia’s antineoplastic properties [246].

A number of studies have shown the anticancer properties of frankincense [249]. A study on the cytotoxicity of oleo gum resin fractions revealed anticancer activity at the IC50 levels even lower than for doxorubicin and 5-fluouracil [250].

An in vivo study has demonstrated that frankincense suppressed melanoma in C57BL/6 mice with no detrimental effects on body weight; observable histopathologic differences in the brain, heart, liver, and kidney tissues; and hematological biochemical parameters [251]. The cytotoxicity was associated with a decreased Bcl2/BAX ratio.

A number of papers are devoted to the anticancer properties of BAs and their natural variants [246]. They down-regulate NF-kb and STAT3 [252,253], MAPK, AKT, ERK1/2, and other key signaling mediators.

As a possible mechanism of activity, Shen and colleagues [254] have shown that BAs may induce epigenetic alterations by modulating DNA methylation. The authors have shown that, in CRC cell lines, there was a modest increase in genome-wide DNA demethylation. This resulted in the re-expression of SAMD14 and co-suppressor genes SMPD3, as well as in the inhibition of DNMT activity. In line with this evidence, Mazzio and colleagues [255] have carried out transcriptomic profiling of TNBC MDA-MB-231 cells treated with *Boswellia serrata* or 3-O-acetyl-B-boswellic acid. They found that this treatment elicits the activation of several key components of the PERK pathway (unfolded protein response (UPR))—PERK, CHOP, GADD34, and ATF3), the induction of tumor suppressor genes and mTOR inhibitors (e.g., sestrin 2 (SESN2)), and Tribbles homolog 3 (TRIB3). On the contrary, this treatment inhibited the hyaluronan binding (CEMIP) of oncogenes, transglutaminase 2 (TG2), and SRY box 9 (SOX9) which was associated with cell death induction.

Taken together, *Boswellia serrata* and BAs possess significant antineoplastic effects. BAs are considered to be excellent structures to develop lead compounds which may also be conjugated with other therapeutic drugs [256]. Numerous semisynthetic BAs have been developed with very good cytotoxicity [257].

The phase 1 clinical trial on Boswellia (which is the extract from frankincense) for breast cancer primary tumors (NCT03149081) is ongoing and intends to assess its influence on markers of proliferation, angiogenesis, and apoptosis.

Plants of the genus ***Xanthium*** (the *Asteraceae* family) are sources of bicyclic sesquiterpene lactone xanthatin. Several species, including *Xanthium strumarium*, have been used as medical plants in Ayurveda, Chinese, and other traditional Asian medical systems.

In triple negative breast cancer MDA-MB-231 cells, xanthatin decreased the catalytic function of topoisomerase II, which led to GADD45γ up-regulation and arrested cells in the G2-M state [258]. Using the same breast cancer cell model and mouse xenografts, other authors have shown that xanthatin inhibits vascular endothelial growth factor receptor 2 (VEGFR2), reducing VEGF-stimulated angiogenesis, microvessel density, and tumor growth [259].

In A549 cells (NSCLCs), xanthatin disrupted NF-κB signaling and induced p53, which resulted in G2-M arrest and the activation of the intrinsic apoptotic pathway [260]. Xanthatin also suppressed NSCLC by diminishing STAT3 and GSK3β transcription factors [261,262].

Through its application to child retinoblastoma cell models and zebrafish xenografts, Yang and colleagues have shown that xanthatin targets polo-like kinase 1 (PLK1), mediating G2-M cell cycle arrest and apoptosis [263]. In colon cancer cells, xanthatin similarly stopped cells in G2-M. It elevated ROS, autophagy, and apoptotic response [264], while suppressing glycolysis and mTOR signaling [265].

Xanthatin induced the cell death of glioma cell lines and xenografts via the elevation of endoplasmic reticulum (ER) stress-related proteins, including glucose-regulated protein 78 C/EBP-homologous protein (CHOP) [266].

In this way, xanthatin is very interesting in terms of antitumor therapy.

In general, many Asian plants are known with neoplastic properties described in reviews [267,268,269,270]. In addition, other plants and their active substances with emerging antitumor activities have recently been identified [271,272], which indicates that there are many such plants that have not yet been discovered.

Other major medical plants traditionally used in ayurveda for cancer healing are described in the excellent review [2].

### 3.4. Europe

***Hypericum perforatum*** (HP, St. John’s wort, SJW, the *Hypericaceae* family) is a flowering plant native to Europe and Asia. It was also introduced to North and South America, South Africa, and Australia. HP is still used in folk medicine and now is commercially grown in different countries. It is effective in the treatment of anxiety and depression which is mediated by inhibiting the uptake of monoamine neurotransmitters (serotonin, dopamine, noradrenaline, GABA, and L-glutamate) [273]. *HP* is effective against inflammation and urinary cystitis.

The pharmacological properties of *Hypericum perforatum* are associated mainly with hyperforin (HPF) and hypericin, which usually present in a total hydro-alcoholic HP extract concentrations ranging between 1 and 5% and 0.1 and 0.3%, respectively [274]. Other *HP* constituents are hyperoside, rutin, quercetin, catechin polyphenols, acylphloroglucinols, and naphthodianthrones [275].

*HP* extracts and hyperforin decrease the inflammation by suppressing 5-lipoxygenase (5-LO), cytochrome c oxidase subunit I (COX-1) activity [276], and prostaglandin PGE2 production [277]. It also reduces ROS [278] and genotoxic stress [279]. These, and other, properties of *HP* protect from carcinogenesis [274].

In multiple studies, both *HP* extracts and hyperforin have demonstrated antineoplastic activity against different types of malignancies. They attenuated cell growth, angiogenesis, and metastases, while inducing apoptosis (reviewed in [274]). Hyperforin inhibited EGFR/ERK/NF-κB [280] and AKT [281] signaling pathways. It suppressed antiapoptotic proteins Bcl-2 and Mcl-1 [282]; reduced the production of angiogenic chemokines CXCL8 and CCL2 [283]; and decreased metalloproteasis MMP2, MMP9, elastase, and cathepsin, which are important for migration and invasiveness [284].

It is interesting to note that the mechanism of hyperforin’s antineoplastic activity is thought to be associated with its protonophor properties. Due to a low ∆pH between intercellular space and cytosol in non-cancer cells, protonofor hyperforin does not significantly change their intracellular pH. At the same time, the ∆pH of cancer cells is much higher because of the acidic extracellular space and more basic cytoplasm. In this case, hyperforin induces the increased H+ influx which leads to cytosol acidification and interferes with biochemical processes in malignant cells [274,285].

The analysis of 87 HP samples which were collected from 14 countries concluded that there was limited chemical variability [286]. In view of HP’s inclusion in European Pharmacopeia and standardization opportunities, it can be considered a potential anti-neoplastic substance.

***Betula pubescens*** (*BP*, syn. *Betula alba*, the *Betulaceae* family), commonly known as white birch, is native and abundant throughout northern Europe and northern Asia. Its bark contains betulinic acid (BA)—a lupane-type pentacyclic triterpenoid saponin.

In tumor cells, BA activates the mitochondrial pathway of apoptosis. It permeabilized the outer mitochondrial membrane, and also induced cytochrome c release and caspase activation [287].

Several signaling pathways are shown to be affected by BA [288]. It dampens STAT3 and HIF-1α which reduce angiogenesis. BA augments the MAPK/p38 and JNK signaling pathways [289]. Guo and colleagues have shown that mTOR signaling was targeted by BA which initiated apoptosis [290]. In turn, another group has demonstrated that BA suppressed p-AKT and mTOR while inducing autophagy [291]. Apparently, this autophagic response can mitigate BA-induced mitochondria-dependent cell death [292].

In breast cancer cells, BA attenuated glycolysis and respiration [293]. It is important to note that BA attenuates the expression of multidrug resistance proteins P-gp, BCRP, and ABCB5, hence decreasing chemotherapeutic resistance mediated by mutant EGFR [294].

To improve the water solubility and antineoplastic activity of BA, different research groups are currently working on the synthesis of its soluble derivatives [295].

***Glycyrrhiza glabra*** (*GG*, “Liquorice”, the *Fabaceae* family) is an herbaceous perennial legume flowering plant native to Europe, Western and Central Asia, Siberia, and Northern Africa. This plant was used in folk medicine in ancient Egypt, Rome, Greece, and China, and has been included in Ayurveda.

*GG* has a rich biochemical composition as 400 compounds were isolated from this plant. The most important among them are triterpenoid glycyrrhizin; saponin glycyrrhizic acid; the flavonoids liquiritin and isoliquiritin; and the isoflavones glabridin and hispaglabridins A and B [296]. This set of chemicals mediate a huge number of beneficial pharmacological properties, including antitussive, expectorant, antimicrobial, anticoagulant, and memory-enhancing activity, as well as antioxidant, anti-inflammatory, antidiabetic, hepatoprotective, immunomodulator, and antineoplastic capabilities.

Regarding the antineoplastic properties of *GG*, isoliquiritigenin (2′,4′,4-trihydroxychalcone, ISL) is one of the most important compounds extracted from licorice roots. ISL displays a suite of antitumor properties [297]. In lung cancer cell models, ISL inhibited proliferation by interfering with AKT/mTOR [298] and FAK/Src signaling pathways [299]. Src family kinase (SFK) transmits signals from integrins, growth factors, and G protein-coupled receptors to AKT/mTOR, MAPK/ERK, and Hippo signaling pathways. Src/FAK mediates modifications in the actin cytoskeleton and focal adhesion complexes, facilitating migration and invasion [300]. In renal carcinoma cells, ILS down-regulates Jak2/STAT3 and MDM2 ubiquitin ligase. MDM2 possesses oncogenic properties, i.e., the main negative regulator of the p53 tumor suppressor [301], as well as through various p53-independent ways [302,303]. ISL treatment of melanoma cells decreased miR-301b and recovered its target leucine-rich repeats and immunoglobulin-like domains 1 (LRIG1) which down-regulates tumor growth [304].

In silico docking experiments suggest that ISL can possibly directly target VEGF-2, both wild-type and double-mutant (L858R/T790M) EGFR, 78-kDa glucose-regulated protein (GRP78), sirtuin 1 (SIRT1), COX-2, and Ikkb [297].

Besides ILS, other compounds of GG (glycyrrhizin, glycyrrhizinic acid, etc.) possess antineoplastic activities (reviewed in [297]). This, together with the safety of GG, which is proven by centuries of use, makes this plant a very promising candidate for anticancer therapy studies.

***Silybum marianum*** (***SM***, “milk thistle”, the *Asteraceae* family) is a biennial herb, 30 to 200 cm tall with red-to-purple-colored flowers. Its native distribution area includes the Mediterranean Sea coast, the coast of southeast England, Iran, and Afghanistan. However, this plant was introduced to other continents and was cultivated due to its medical properties.

*SM* is important for medicine as a source of silymarine, which is the standardized extract from the thistle milk seeds, containing at least seven flavonolignans (silybin A, silybin B, isosilybin A, isosilybin B, silychristin, isosilychristin, and silydianin) and one flavonoid (taxifolin). Symilarin accounts for 65–80% of *SM* seeds. Its compounds provide the main SM pharmacological activity. The important pharmacological activity also has silybinin which is a semi-purified fraction of silymarin, consisting of a mixture of two diastereoisomers, silybin A and silybin B, in an approximate 1:1 ratio [305].

Silymarine possesses hepatoprotective (chronic hepatitis B and C, alcoholic hepatitis, fatty liver disease, and cirrhosis), antidiabetic, anti-ischemic, and skin-protective properties, as well as others [306]. In the oral cancer models, sylimarin induced the extrinsic apoptotic pathway, decreased tumor volumes, and prolonged mouse lifespan [307]. In human colorectal cancer cells, silymarin may down-regulate the Wnt signaling pathway through β-catenin proteasomal degradation and TCF4 transcriptional inhibition [308].

Silibinin also possesses anticancer activities: the inhibition of proliferation, migration, and metastasis; angiogenesis; and the induction of apoptosis due to the down-regulation of EGFR, Akt, MAPK, and Wnt signaling pathways [309,310]. Silibinin suppressed the growth of human gastric cancer cells by down-regulating MAPK signaling. In the TNBC cell line MDA-MB-231, this compound also reduced the TGF-β-mediated expression of fibronectin and metalloproteinases MMP2, MMP9, and metastasis in xenograft models [311]. In hepatocellular carcinoma models, the combination of silibinin with sorafenib was demonstrated to down-regulate Akt-STAT3 signaling, anti-apoptotic proteins (Bcl-2 and Mcl-1), and stemness-related proteins (homeobox transcription factor (NANOG)) and Krueppel-like factor 4 (Klf4) [312].

Several studies have demonstrated that silymarin is safe for humans and is tolerated even at a high dose of 700 mg three times a day for 24 weeks (reviewed in [313]). This obstacle, in light of its anti-neoplastic and hepatoprotective capabilities, as well as the presence of standardization, makes silymarin the excellent candidate for cancer treatment, especially for hepatocarcinoma.

Some other European plants and their compounds with anti-neoplastic activity are listed in Table 4.

### 3.5. North America

***Panax quinquefolius*** (*PQ*, Panax americanus, the *Araliaceae* family) is a perennial herbaceous plant 30–100 cm high with a thick tuberous rhizome. It mainly grows in the USA, in the wooded areas of Maine and Missouri, and in Canada, in the provinces of Ontario, British Columbia, and Quebec. It is known that various Indian peoples took decoctions and infusions from the ginseng root to treat ulcers, asthma, and various inflammatory eye diseases, as well as to increase fertility levels. PA is a close relative of Panax ginseng which is the most widely used ginseng in China, Korea, and Japan. These plants have similar pharmacological properties.

According to the FDA, ginsengs are generally recognized as safe (GRAS) plants, and their inhibitory effects on malignant tumors have been widely accepted in the USA and Europe [328]. Ginseng is characterized by the presence of ginsenosides, which are ginsengs triterpenes saponins (Rx), considered to be the main bioactive compounds of ginseng. They are also metabolized by the gut microbiota to undergo sequential de-glycosylation and are finally converted to prosaposin or sapogenins within the human body. Based on the structure of aglicon, Rx may be divided into five types: panaxatriol saponin, protopanaxadiol, protopanaxatriol, oleanolic acid, and ocotillol types [329].

Various ginsenosides have demonstrated anticancer properties in vitro and in vivo following the inhibition of cell cycle, angiogenesis, and the induction of apoptosis in different types of malignancies [328,330].

Ginsenoside Rg3 reduced colon carcinoma in HCT116 cells, whereas its derivate 20(S)-protopanaxadiol effectively attenuated NF-κB, JNK, and MAPK/ERK signaling pathways [331]. Other ginsenosides, Rb3, R1, and Rc, bound Hsp90α, suppressing the activity of SRC and PI3K kinases. This led to the inactivation of Akt and ERK pathways and lung cancer suppression [332]. In patient-derived xenograft mouse models and glioblastoma stem cell lines, ginsenosids Rg3 and Rh2 suppressed cell viability and the self-renewal capacity of GSCs via the inhibition of the Wnt/β-catenin signaling pathway [333]. In pancreatic cancer in vitro and in vivo models, Rg3 treatment reduced the levels of vasculogenic mimicry, matched with the decrease in VE-cadherin, EphA2, MMP-2, and MMP-9 mRNA [334].

The structure–activity relationships of ginsenosides and the molecular mechanisms of their actions are summarized in the following review [335]. According to literature data surveys, Rh1, Rh2, and Rg3 have strong anti-cancer activities. Because of a number of biologically active compounds identified, as well as FDA-reported safety concerns, Panax-standardized plant material and ginsenosides are promising candidates for anti-neoplastic adjuvant therapy.

Some other North American plants and their compounds with anti-neoplastic activity are listed in Table 5.

### 3.6. Australia

Although Australia is a habitat for more than 21,000 plant species, there is extremely limited information about their medical use by indigenous peoples. There is especially little data on their anti-neoplastic properties. The well-known example is ***Eremophila galeata*** (*EG*, the *Scrophulariaceae* family), a flowering shrub which is endemic to Western Australia. This plant has a long history of use in medicine by indigenous peoples because of its valuable pharmacological properties [345].

Petersen and colleagues identified that the crude extract prepared from EG leaves significantly sensitized HT-29 cells to SN-38—a modern topoisomerase I inhibitor. One of its major compounds, the 5,3′,5′-trihydroxy-3,6,7,4′-tetramethoxyflavone, strongly suppressed the breast cancer resistance protein (BCRP/ABCG2) [346] which belongs to the family of ATP-binding cassette proteins. BCRP mediates multidrug resistance and promotes an efflux of such potent drugs, such as methotrexate, irinotecan, topotecan, sorafenib, gefitinib, and doxorubicin, from cancer cells [347].

Some other medical Australian plants with anticancer properties are listed in Table 6. Thereby, Australian plants are extremely unstudied to date in terms of antitumor properties.

## 4. Mushrooms

About 2.2–3.8 million fungi exist on our planet, including 14,000 mushroom species [354]. Interest in mushrooms as a medical supply is rooted in the mists of time and prevails to these days. Mushrooms are used in the traditional medicine of China, Ayurveda, East Asia, Europe, South America, etc. A number of mushroom species are implicated as food supplements to improve health in different regions, including the USA and Europe. Several big company sale food supplements contain, or are fully derived from, mushrooms. For instance, iHerb (USA, California, www.iherb.com; accessed on 30 March 2022), Fungi Perfecti (USA, Olympia, www.fungi.com; accessed on 30 March 2022), Ommushrooms (USA, Carlsbad, www.ommushrooms.com; accessed on 30 March 2022), Terezia (Czech Republic, Praha, https://www.terezia.eu/en/; accessed on 30 March 2022), Realmushrooms (Canada, Roberts Creek (BC), www.realmushrooms.com/; accessed on 30 March 2022), Time Health (UK, www.timehealth.co.uk; accessed on 30 March 2022), Zipvit (UK, Staffordshire, https://www.zipvit.co.uk/; accessed on 30 March 2022), Hangzhou Molai Biotech Co., Ltd. (China, Hangzhou, https://phytonutri.en.made-in-china.com; accessed on 30 March 2022), and Shaanxi Shineherb Biotech Co., Ltd. (China, Shaanxi, www.shineherb.en.made-in-china.com; accessed on 30 March 2022) are among them. The main mushrooms which are explored by humans as beneficial for health are reishi, cordyceps, turkey tail, maitake, lion’s mane, chaga, and others.

Nowakowski and colleagues have summarized 92 mushroom species with antineoplastic activity, which could be effective against 38 various cancers [355]. Mushrooms display a great number of secondary metabolites with different biological activities [356,357,358]. In addition, these metabolites are different from secondary metabolites of plants. Regarding cancer healing, mushroom and fungi, in general, as well as their biochemical diversity, are almost fully unexplored to date.

Below, we give some examples of mushrooms that possess antitumor properties and have been used in traditional medicine.

***Lentinula edodes*** is a mushroom that grows in East Asia. It is known as “Xianggu” in China and “Shiitake” in Japan. The mushroom has been used as food and in traditional Chinese medicine for at least 2000 years. It possesses analgesic, tonic, and antiparasitic activities [359].

Preclinical studies have identified that shiitake has immunostimulating, antibacterial, antiviral, hepatoprotective, antimutagenic, antihypercholemic, and anticancer properties due to the content of lentin, lignin, and erytadenine in the fruiting body [359].

Among others, shiitake chemical composition includes polysaccharides, polysaccharopeptides, lectinss, and lentinan, the last of which is especially focused on the medical attributes of this mushroom [355].

The polysaccharide lentinan (1,3 beta-d-glucan), when isolated from shiitake, has shown strong antitumor properties. There are studies demonstrating the existence of the direct cytotoxic effects of shiitake extracts on cancer cells in parallel with minimal impact on non-malignant cells. One group reports the direct apoptotic effects of shiitake mycelia extracts on human hepatocellular carcinoma cells with minimal toxicity to normal rat cells [360]. Other researchers have shown the direct cytotoxic effects of fruit bodies, but not mycelia extract, on MCF7 cells, with far less significant cytostatic effects on fibroblasts [361].

In several in vitro studies, the synergistic effects of lentinan with docetaxel, paclitaxel, and cisplatin on proliferation and apoptosis have been shown. Lentinan sensitized lung cancer cells to paclitaxel through ROS-TXNIP-NLRP3 inflammasome and ASK1/p38MAPK signaling pathways [362]. It also sensitized bladder cancer to gemcitabine [363] and gastric cancer cells to docetaxel and cisplatin [364]. Lentinan increased the sensitivity of HepG2 hepatoma cells and xenograft H22-bearing mice to oxaliplatin, which was associated with NF-kb, STAT3, and surviving suppression [365].

One more application may be doxorubicin-conjugated lentinan nanoparticles, which increased cytotoxicity for breast cancer while decreasing it for human normal cells [366].

The direct antitumor activity of water-extracted polysaccharide on cancer cells has also been demonstrated using athymic nude mice and human colon cancer cells [367]. In this model, lentinan-induced ROS mediated both TNF-α and mitochondria-dependent apoptosis.

Nevertheless, the main mechanism of the lentinan-mediated anticancer response is proposed to be associated with the stimulation of the immune system. Different mechanisms are suggested to be responsible for this. The modulation of the TLR4/dectin1-MAPK and Syk-PKC-NFκB signaling in immune cells is reported. In patients with digestive cancer, lentinan removed the dominant state of Th2 which restored Th1-Th2 lymphocyte (Tregs) balance [368,369,370,371]. Th1 cells possess antitumor activity and produce IFN-γ and IL-12, whereas Th2 is characterized by IL-4 and IL-10 production and may promote malignization [372,373].

In clinical concentrations, lentinan down-regulated PD-L1 which enhances the efficiency of adaptive immunity.

Lentinan decreased the granulocytes–lymphocytes (G/L) ratio in gastric cancer patients opposed to those who have only received chemotherapy, and prolonged their survival [374]. The G/L ratio (neutrophil–lymphocyte ratio) is suggested as a prognostic marker, and is associated with an increased tumor progression, invasion, and shortened survival in different types of malignancies including gastric cancer [375,376]. Solid tumors express granulocyte colony-stimulating factor (G-CSF) which induces the proliferation of leukocytes (neutrophils) and myeloid-derived suppressor cells (MDSCs). Both of them suppress the proliferation of lymphocytes and lymphocyte-activated tumor cells killing those which favor malignization [377]. Lentinan was shown to decrease the G-CSF serum level and inhibited MDSCs via a CARD9-NF-κB-Ido pathway which may be responsible for a decrease in the G/L ratio and partially responsible for anticancer properties [378,379].

Wang and colleagues reported that the addition of lentinan to the combination therapy of vinorelbine and cisplatin in a cohort of 73 patients with NSCLC resulted in an approximately two-fold increase in NKT-cells [379]. This was accompanied by the shift of Tregs status from Th2 to Th1, in accordance with the elevation of IFN-γ, TNF-α, and IL-12.

In China and Japan, lentinan was used as an adjuvant therapeutic drug. The meta-analysis of 650 gastric cancer patients has shown that lentinan significantly increased their survival and was mostly effective in patients with lymph node metastasis [380]. Lentinan also increased the lifespan of patients with hepatocellular carcinoma [381] and improved the quality of life of patients with unresectable pancreatic cancer [382].

Zhang and colleagues reported about 9500 cancer patients who were treated with lentinan for a period of 12 years [371]. A number of studies demonstrated that lentinan improved a patient’s survival rate, seemingly irrespective of the tumor type [374,383].

Nevertheless, the mechanisms of this phenomenon are not fully understood today. In summary, shiitake and lentinan are valuable for cancer treatment, but further intensive studies of their antineoplastic mechanisms with possible side effects and limitations are required, as well as well-designed clinical trials.

***Ganoderma lucidum* (*Gl*)** is a mushroom that grows on plum trees in many Asian countries. It is commonly known as “Reishi” in Japan and “Ling-zhi” in China. In traditional Chinese medicine, reishi has been called the “mushroom of immortality” or the “spirit plant” and has been actively used to prevent cardiovascular diseases; strengthen the immune system; and cure neurological afflictions, allergies, and liver disorders for many centuries [384]. Moreover, reishi is a part of adjuvant therapy of cancer and diabetes.

As in the case of shiitake, reishi suppresses tumor cells both directly and through fine-tuning of the immune system. Severe combined immunodeficient (SCID mice) cells, bearing human inflammatory breast cancer cells, when treated with *Gl* extract, significantly reduced tumor growth and weight, accompanied with the attenuation of Ki-67, vimentin, p-ERK1/2, Akt, and mTOR (as well as its targets p70S6K and eIF4G) [385]. The in vitro model has also proven the reishi-mediated suppression of protein synthesis and proliferation, whereas it was not toxic to non-tumor breast MCF10A cell lines [386]. The *Gl* extract was able to attenuate lamellipodia formation, thus inhibiting the motility of MDA-MB-231 breast cancer cell lines. This was associated with a reduction in Rac kinase activity, as well as p-FAK (Tyr925), Cdc42, and c-Myc expression [387].

Different compounds with medical properties have been identified in reishi extracts. Although the plethora of them may be responsible for antitumor activity, ganoderic acid (GA) and *Ganoderma lucidum polysaccharides* (*GLPs*) are proposed to be the most important of them [388]. The antineoplastic activity of reishi is manifested as both direct cytotoxicity to cancer cells or indirect cytotoxicity through the stimulation of the immune system [389].

GA is a natural triterpenoid whose molecular structure is similar to steroid hormones and has multiple isoforms [390]. It is proposed that GA targets several receptors (IGFR-1, VEGFR-1 and -2, and ER) [391] and is shown to inhibit the PI3K/Akt/mTOR pathway [392], induce DNA damage [393], down-regulate MMP-2 and -9 [394], and affect other oncogenic activities [388].

*Ganoderma lucidum polysaccharides* (*GLPs*) are considered to be the main antitumor compound of reishi [389,395]. *GLP* inhibited autophagic flux in colorectal and gastric cancer cells [396,397] and suppressed “aerobic glycolysis” (the Warburg effect) [398]. It down-regulated vimentin and EMT-associated TF Slug, and also inhibited the JAK/STAT5 pathway, motility, and the invasion of ovarian cancer cells [399]. Water-soluble glucose-enriched *Gl* polysaccharide attenuated the activation of EGFR and Akt, suppressed oral cancer cells, and sensibilized them to cisplatin, while protecting normal human oral epithelial cells from cisplatin-mediated cytotoxicity [400].

The major antitumor activity of GLP occurs through the modulation of the immune system [401]. GLP increased the proliferation and differentiation of B-lymphocytes, the activity of T-lymphocytes, and their IFN-γ production [402]. It increased several-fold the number of natural killer (NK) cells [403], and also increased the granulocyte–macrophage colony-stimulating factor (GM-CSF), the granulocyte colony-stimulating factor (G-CSF), and the macrophage colony-stimulating factor (M-CSF) [404].

Zhang and co-authors developed gold GLP composite nanoparticles which activated dendritic cells, promoted the proliferation of T killers and Tregs in splenocytes, elevated the percentage of CD4+/CD44+ memory T cells, and reduced tumor weight and metastasis in the 4T1 breast cancer mouse cell model [405].

*GLP* may be a promising prebiotic substance for the treatment of colorectal cancer. Using a mouse model of inflammatory colorectal cancer, Guo and colleagues reported that *GLP* treatment normalized dysbiosis; improved the gut barrier function; and suppressed IL-1β, iNOS, COX-2, and macrophage infiltration [406].

A randomized double-blind placebo-controlled study has shown beneficial effects for healthy volunteers upon *Gl* intake in terms of hepatoprotective and antioxidant activity [407]. An evaluation of 120 breast and lung cancer patients whose treatment was supplemented or not with *Gl* revealed the reverse correlation between *Gl* intake and immunosuppressive factors COX2 and TGF-β1 and positive correlation with anticancer IL-12 [408].

In conclusion, reishi is a safe non-toxic plant, and has been utilized as an alternative adjuvant in the therapy of cancer patients without obvious toxicity. It acts in synergy with antineoplastic drugs and is used clinically to treat various malignancies [389]. It deserves more attention as a potential adjuvant.

***Grifola frondosa***, commonly known as maitake, is an edible and medicinal mushroom that grows in Asian regions, especially in China, India, Japan, Korea, and some European countries. It has been used for centuries in traditional medicine for different purposes. The anticancer properties of this mushroom are especially attractive.

Several bioactive polysaccharide fractions could be separated from Gf: D-fraction, MD-fraction, X-fraction, Grifolan, MZ-fraction, and MT-α-glucan, which possess different biological activities [409]. For medical usage, in most cases, the so-called “D-Fraction” is prepared via extraction from fruit bodies. In this way, D-fraction is a standardized form of protein-bound β-glucans (proteoglucans) extracted from the fruit bodies of maitake. It predominantly contains β-d-glucans with β-(1→6) main chains and β-(1→4) branches, as well as more common β-(1→3) main chains and β-(1→6) branches [410].

It was shown that in the MDA-MB-231 TNBC cell line, D-fraction favored apoptosis, decreased motility, increased E-cadherin protein levels and β-catenin membrane localization, and reduced activity of MMP-2 and MMP-9 [411,412]. In the corresponding xenograft mouse model, D-fraction also inhibited tumor growth and metastasis.

The inhibitory effect was associated with the cell cycle arrest, diminished motility, and induced apoptosis. D-fraction suppressed hepatoma cells both in vitro and in vivo, which was associated with PI3K/AKT attenuation and an autophagy increase [413]. The Konno group demonstrated the strong synergistic cytotoxicity of D-fraction combined with vitamin C on prostate and renal cancer cells [414,415].

However, numerous studies have shown that the key ability of maitake to affect tumors is hidden in the stimulation of the immune system. Both the innate and acquired immunities are affected by D-fraction. In BALB/C mice, D-fraction blocked more than 60% of breast cancer development and prevented oncogenesis in 26%, with regards to control animals [416]. The other group has shown a long-term immunity activation in MM46-bearing C3H/HeN mice which was associated with an increase in TNF-alpha, IFN-gamma, and macrophage-derived interleukin (IL)-12, as well as the activity of NK cells [417]. Furthermore, D-fraction combined with vitamin C increased the percentage of CD4 + CD8 + T-cells, B-cells, and Treg cells, and also elevated IL-2, IL-12p70, TNF-α, and IFN-γ levels in Heps-bearing mice [418].

In B16 melanoma and colon-26 carcinoma mice, maitake-derived α-glucan (a highly α-1,6-branched α-1,4 glucan, YM-2A) elevated the antitumor immune response through the up-regulation of INF-γ-expressing CD4+ and CD8+ T-cells in the spleen and INF-γ-expressing T-CD8+ cells in tumor-draining lymph nodes. Moreover, orally administered YM-2A increased the expression of the MHC class II and CD86 on dendritic cells and the MHC class II on macrophages in Peyer’s patches [419].

The meta-analysis of pre-clinical data revealed that Gf usage upon cancer treatment significantly inhibited tumor growth, and, on the contrary, improved remission rates, and also increased CD4+ and CD8+ T cell percentages, as well as IL-2, IL-12, and TNF-α [420].

Maitake-derived polysaccharide-based drugs were subjected to clinical trials. The Japan group reported cancer regression in about 58.3% of liver cancer patients, 68.8% of breast cancer patients, and 62.5% of lung cancer patients [421]. At the same time, there was only a 10–20% improvement for leukemia, stomach cancer, and brain cancer patients. In another investigation, D-fraction increased NK cell activity, attenuated metastatic progress, and improved the expression of tumor markers in all examined patients [422].

In China, a maitake-derived polysaccharide-based drug was approved by the State Food and Drug Administration (SFDA) in 2010 [423].

Strong antitumor properties and the safety of its use place maitake at the top of biological organisms which should be studied with respect of neoplasia.

***Cordyceps sinensis* (*CS*)** and ***Cordyceps militaris* (*CM*)** are important mushroom species for China and Korea. Both of them are entomopathogenic fungi which parasitize on the larvae of moth caterpillars. However, these mushrooms can be cultivated in a variety of media, including silkworm pupae, rice, and liquid nutrition. They have been used in Chinese medicine because of their anti-inflammatory, anti-microbial, immunostimulant, and antineoplastic properties [424]. The known bioactive compounds of these mushrooms are cordycepin, cordycepic acid, ergothioneine, lovastatin, and polysaccharides [425,426].

In the 4T1 orthotopic xenograft breast mouse model, an extract of Cs inhibited tumor growth and promoted macrophage polarization toward the M1 phenotype [427]. Cm extract was shown to suppress KRAS-driven colorectal cancer by attenuating the RAS/ERK pathway [428]. Another study reported that the Cm extract overcame cisplatin resistance in NSCLC cell lines when proteomic profile analysis revealed H-Ras down-regulation [429]. Other authors have demonstrated that its extract down-regulated hedgehog signaling in NSCLCs via TCTN3 inhibition and GLI1 nuclear translocation suppression [430].

The main pharmacologic activity of CS and CM is attributed to cordycepin. This is 3-deoxyadenosine, which has a similar structure to adenosine but lacks the 3′-hydroxyl group of the ribose moiety [431]. Adenosine receptors are in the family of G-protein-coupled receptors, which are found in almost all human body tissues and organs. Specific ligands, agonists, or antagonists activate these receptors which modulate tumor growth via a range of signaling pathways [432].

Cordycepin is suggested to act through ADORA2 and ADORA3 receptors. It has been shown that the cordycepin-mediated activation of ADORA3 inhibits growth and induces apoptosis in bladder cancer and murine B16 melanoma, which can be associated with glycogen synthase kinase-3β activation and cyclin D 1 suppression [433,434,435]. In the HCC model, cordycepin suppressed focal adhesion kinase (FAK) activation which plays an important role in angiogenesis [436,437]. Cordycepin down-regulates PI3/AKT, MAPK/ERK, β-catenin, bcl-2, and cdk2, and also induces JNK, caspase-3 and -9, and PARP cleavage in renal, colon, bladder, lung, breast, prostate, glioblastoma cancer, and leukemia. This compound inhibited cell cycle, motility, invasion, and vascularization, while inducing apoptosis (reviewed in [431,438]). One more mechanism has been proposed for cordycepin neoplastic activity. It activates death receptors (DRs) which induce extrinsic apoptotic pathways [439,440]. With respect to the testicular cancer mouse model, cordycepin suppressed FGFs/FGFRs pathways, ERK1/2, Rb/E2F1, cell cycle, and tumor growth [441].

Cordyceps acid diminished lung cancer development in nude mice which was associated with the inhibition of the Nrf-2/HO-1/NLRP3/NF-κB pathway in tumor tissue [442].

Like other mushrooms discussed, Cordyceps possess immunomodulatory effects. It is assumed that this effect is mainly attributed to polysaccharides. The mushroom is able to increase the production of interleukin (IL)-1β, IL-2, IL-6, IL-8, IL-10, and IL-12, as well as the tumor necrosis factor (TNF)-α, and also induce the phagocytosis of macrophages and mononuclear cells [443,444,445]. Thus, cordyceps are able to strengthen the immune system, which is an additional bonus for cancer therapy.

A *cordyceps sinensis*-derived polysaccharide provoked apoptosis and autophagy in human colon HCT1166 cells, which were associated with Akt, mTOR inhibition, and AMPK and ULK1 activation [446].

Ergothioneine is a diet-derived amino acid which exhibits antioxidant, cytoprotective, and other activities beneficial to human health [447]. It likely enters the cells by binding the solute carrier family 22, member 4 (SLC22A4), which is an organic cation carrier. Although there is not enough information about the role of ergothioneine in human physiology, there are strong evidences about its protective properties in our organism [447]. Ergothioneine mitigated oxaliplatin-induced peripheral neuropathy in rats (Nishida 2018), provoked necroptosis in colorectal cancer cells [448], and favored adjuvant vaccine cancer immunotherapy by suppressing the function of tumor-associated macrophages [449]. The blood level of ergothioneine was negatively associated with the risk of cardiometabolic disease and mortality [450], as well as chronic peripheral neuropathy upon colorectal cancer chemotherapeutic treatment [451].

Like reishi, shiitake, chaga, and maitake, the natural Cordyceps-derived products are manufactured and commonly sold as healthy food products.

***Chaga*** (*Inonotus obliquus*, the *Hymenochaetaceae* family) is a plant parasitic fungus, predominantly widespread in Russia and in the countries of Northern Europe. Chaga penetrates into the trunks of various tree species through wounds in the bark, but its main host is birch. Chaga has been used in folk medicine, especially in Russia, Baltic countries, Korea, China, and Japan. As a medical plant, it was first mentioned by Hippocrates [359].

Different types of Chaga extracts have demonstrated their antineoplastic properties in both in vitro and in vivo models (reviewed in [452]).

Chaga contains biologically active polysaccharides, hispidin analogues, melanins, ergosterol, sesquiterpenes, triterpenoids, and benzoic acid derivates. Eighty-six of them are listed with the examples of their antineoplastic properties in [452].

In the Lewis lung mice carcinoma model, the extract of chaga decreased the size of tumors by 60%, and, in parallel, reduced the number of metastatic nodules [453].

In the orthotopic 4T1 mouse mammary cancer model, chaga extract induced autophagy, as well as LCIII and AMPK phosphorylation [454]. Authors have also shown that both inotodiol- and trametenolic-acid-enriched fractions displayed cytotoxicity. Tramentolic acid was shown to decrease the expression and activity of P-gp, which reverted multidrug resistance in breast cancer cells [455].

*Inonotus obliquus* polysaccharides (IOPSs) are considered to be very important biologically active compounds derived from this mushroom. Their hypoglycemic, antioxidant, anti-inflammatory, and neuroprotective properties, among others, have been identified [456].

The intraperitoneal administration of IOPSs at a dose of 30 mg/kg/day led to 4.07-fold increase in the survival rate of B16F10-implanted mice. Moreover, the authors reported that approximately 67% of the initial number of mice survived with no tumor incidence after 60 days of feeding. At the same time, no cytotoxic IOPS activity was observed for both normal and cancer cells in vitro. Thus, the authors suggested that the anti-cancer effects of endo-polysaccharides are associated with immunostimulation [457]. However, another study has shown that Inonotus polysaccharides directly activate autophagy through LKB1/AMPK, which provoked MMP loss as well as the down-regulation of glycolysis and respiration, and subsequently elicited the death of lung cancer cells both in vitro and in allograft tumor models [458].

Other bioactive compounds from chaga are hispidin, hispolon, inotodiol, and syringic acid. They were shown to reduce proliferation, invasion, migration, and angiogenesis. On a molecular level, these bioactive compounds attenuated the expression of MMPs and antiapoptotic proteins that, in turn, were mediated by onco-associated signaling pathways: TNF-alpha signaling, Nox/ROS/NF-kB/STAT3, PI3K/AKT, and ERK1/2 [452,459,460].

Inotodiol is lanostane triterpenoid with anticancer properties. It down-regulated β-catenin, c-Myc, and cyclin D1 in breast cancer [461] and suppressed the migration and invasion of ovarian cancer cells through a p53-dependent mechanism [462].

Hispolon is a natural polyphenol compound with antidiabetic and anti-inflammatory activities which may also kill cancer cells through multiple mechanisms (reviewed in [463]). Hispolon attenuated STAT3 signaling, and also induced S-phase arrest and mitochondria-dependent apoptosis in prostate cancer cells [464]. In melanoma cells, it compromised the activities of mitochondrial respiration complexes I and IV, i.e., the level of Bcl-2, and also increased ROS, nitrite, and lipid peroxide levels [465]. Regarding breast cancer, hispolon, on the contrary, attenuated ROS levels, ERK activity, and the expression of Slug, therefore reversing EMT (Zhao 2016). In another study, hispolon degraded cathepsin S in an autophagy-dependent way which suppressed metastasis [466].

Polyketide hispidin exerts a variety of beneficial properties and may help to reduce cancer, metabolic syndrome, cardiovascular, neurodegenerative, and viral diseases (reviewed in [467]). Hispidin induced the microtubule and depolymerization induced lysosomal membrane permeabilization, which resulted in the death of cancer but not normal cell lines [468]. Moreover, it synergized with gemcitabine to inhibit pancreatic cancer stem cells [469].

Thus, like the well-known Asian medical mushrooms, chaga also has strong antineoplastic properties, both in vitro and in vivo.

Despite the fact that chaga is not as well known as reishi, shiitake, or maitake, and thus was not found associated with any clinical trials, the biodiversity of chaga-derived compounds with strong antineoplastic activities makes this mushroom noteworthy. Additionally, it should be kept in mind as a potential anticancer substance, and therefore warrants further studies.

A number of several mushroom-derived compounds are known today with antineoplastic properties and are of primary interest for cancer investigation. These include various mushroom polysaccharides such as lentinan, D-fraction of Grifola frondose, *Trametes versicolor*-derived PSK, gandoderic acid, grifolin, cordycepin, illudin-*S*, antroquinonol, hispidin, hispolon, inotodiol, theanine, phellinulin A, atractylenolide I, phellifuropyranone, meshimakobnol A, and meshimakobnol B (Table 7 and Table 8).

The antitumor activities of *Grifola frondosa* (Maitake) polysaccharide are reported in a meta-analysis based on preclinical evidence and quality assessment.

## 5. Why Should Medical Plants and Mushrooms Be Used Today?

At its core, modern western medicine has evolved from the folk medicine of different regions around the world over the past few centuries. As stated earlier, the most frequently used anti-neoplastic therapeutics came from live organisms (Tables S1 and S2, Supplementary Materials). Regarding pharmaceuticals, in the process of its evolution, modern medicine has created a certain set of drugs with a known efficacy, safety, side effects, and known molecular targets. However, it lost a wide profile of pharmacological activity of the plant extracts’ initial biological crude material.

Anticancer therapeutics from plants remain extremely important and are still in use to treat various types of neoplasia. They include mitotic poisons from Pacific yew Taxus brevifolia—paclitaxel (Taxol^®^) and its semi-synthetic dodetaxel (Taxotere^®^); vinca alkaloids from Madagascar periwinkle (*Catharanthus roseus* L.)—vinblastine (Velban^®^), vincristine (leurocristin, Oncovin^®^) and their semi-synthetic derivate vinorelbine (Navelbini^®^); topoisomerase I inhibitors, i.e., semi-synthetic analogs of camptothecin from Camptotheca acuminata—irinotecan (Camptosar^®^) and topotecan (Hycamtin^®^); topoisomerase II inhibitor—etoposide (VP-16, Toposar^®^), which is a semi-synthetic derivative of 4’-demethylepipodophyllotoxin from Podophyllum peltatum; and omacetaxine (Synribo^®^)—a semi-synthetic derivate of homoharringtonine from *Cephalotaxus harringtonia*. These drugs occupy the majority of existing chemotherapeutic schemes.

**Figure 2:**
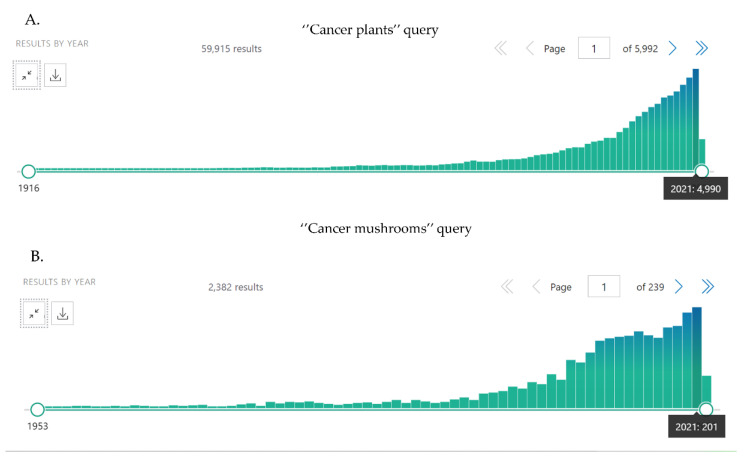
PubMed statistics by years on published articles on plants and fungi with antitumor properties (April 2022). Query on (**A**) “cancer plants” and (**B**) “cancer mushrooms”.

These examples illustrate the importance of plant-derived chemotherapeutics. However, even today, despite being seemingly irrelevant due to a wide assortment of synthetic anticancer drugs, interest in studies of natural compounds from plants and fungi is constantly increasing according to PubMed statistics (Figure 2). It is interesting to note that, although fungi or their active compounds are not clinically used in the western world today, their known safety and use in clinical practice in China and Japan can lead to an increase in studies on their antineoplastic capabilities. These mushrooms are represented by shiitake, maitake, reishi, and others, and act mainly through the stimulation of the anti-tumor immune system.

One of the actual strategies used to develop anticancer drugs is the search for agents which are capable of simultaneously inhibiting several signaling pathways. A large amount of clinical data highlights that the highly selective inhibition of only one of the signaling pathways in the tumor cell usually leads to a limited response. Another significant problem of targeted therapy is the rapid acquisition of resistance by tumor cells due to the proliferation of cell clones bearing mutations that abolish the effects of the targeted drug. Thus, multitargeted therapy is considered a promising approach.

Based on the examples of plants and mushrooms described above, the anticancer activity of their extracts is attributed to the plethora of biologically active compounds with a number of biological activities. Thus, different compounds may target simultaneously different cellular processes resulting in synergistic effects. In light of this, there may be benefits from sharing them with known strong antineoplastic therapeutics in adjuvant or neoadjuvant therapy. The published data of many in vitro and in vivo experiments described here point to the fact that plant and mushroom substances with anticancer properties often increase susceptibility to genotoxic drugs.

In terms of safety and predictability, the usage of individual compounds for therapy is much better than the plant extract, which is a complex mixture of primary and secondary metabolites. However, the well-known phenomenon states that the pharmacological activities of many bioactive constituents are much weaker than those of the corresponding herbal extracts. Upon separation and purification from herbal extracts, the pharmacological effects of many bioactive constituents diminish or even disappear [481,482]. In practice, the pharmacokinetics (AUC values) between some herbal extracts and their pure constituents may differ up to 130 times. This phenomenon depends a lot on the pharmacokinetic synergies during intestinal absorption. This means that additional constituents of plant and mushroom extracts increase solubility, reduce first-pass elimination mediated by drug-metabolizing enzymes and drug efflux transporters (ABC transporters), and enhance the membrane permeability of enterocytes (reviewed in [482]).

For instance, in the *Hypericum perforatum* (St. John’s) extract, the co-existing constituent hyperoside increased the water solubility of the active compound, hypericin, by 400-fold [483]. The antimalaria agent, artemisinin, which is one of the most important natural drugs, is a substrate of cytochrome P450 enzymes. Artemisia annua extract co-occurs with arteannuin B, which inhibits hepatic cytochromes P450 and doubles the peak serum concentration of artemisinin in vivo [484].

There are evidences that coexisting compounds may change the solubility and bioavailability of their active constituents via the formation of natural nanoparticles, greatly modifying their pharmacological activities [482,485,486].

Another important point is associated with a strong deterioration in the health of patients undergoing chemotherapy. In this case, all of the medical plants and mushrooms described here can significantly improve the physical and mental health of patients due to the anti-inflammatory, hepatoprotective, cardioprotective, immunomodulatory, anxiolytic, and metabolism-normalizing properties. The simultaneous use of plant- and mushroom-derived medical substances along with chemotherapy may ameliorate its toxic impact on normal tissues.

Finally, standardized herbal medicine can be more cost-efficient than most other synthetic compounds.

Based on the information discussed in this review, we divided medical plants, mushrooms, and their active compounds into two priority groups for research and potential of use in antitumor therapy. This priority is suggested based on the available literature on their anti-neoplastic efficacy and safety in preclinical and clinical studies (Table 8). The chemical structures of active compounds with their sources are demonstrated in Figure S1.

## 6. Limitations of Using Plants and Mushrooms as Medicine

### 6.1. Bioavailability

Despite the promising antineoplastic activity of several natural herbs and mushrooms, their translation to human studies is limited due to their low bioavailability.

First of all, this is based on poor water solubility. This is a problem limiting the efficiency and application of compounds with significant antineoplastic properties in both animal and human studies. Curcumin, resveratrol, quercetin, hypericin, ursolic acid, silybin, pterostilbene, berberine, betulinic acid, and other valuable compounds are among them [487].

Besides solubility, other reasons affecting bioavailability include an increased intestine metabolism (by both microbiota and enterocytes), absorption and intestinal efflux (the activity of P-gp and other ABC transporters), and the activity of liver drug-metabolizing enzymes. The drug-metabolizing system consists of phase I and phase II drug-metabolizing enzymes which are cytochromes (CYPs), especially CYP3A4, and UDP glucuronosyltransferases (UGTs), primarily UGT1A1 and 2B1 [482]. These enzymes are active in both hepatocytes and enterocytes.

In preclinical and clinical investigations, curcumin, quercetin, resveratrol, and other promising natural compounds with anticancer properties have displayed problems with the dissolvement into gastrointestinal fluids, permeability across the intestinal epithelium, and “first-pass” metabolism due to the aforementioned molecular limitations which greatly reduce oral bioavailability [488]. To challenge this, chemical modifications of natural molecules can be carried out to improve them. However, after chemical modifications, this molecule will not be natural anymore, but rather will become a new compound, which will require new exhaustive preclinical studies.

As an alternative, several approaches have been used including nanoparticle formulations, phytosomes, and the use of bioenhancers [488].

To increase bioavailability, self-microemulsifying drug delivery systems (SMEDDSs) are frequently formulated [489]. SMEDDS are isotropic mixtures of oils, surfactants, or (alternatively) co-surfactants and co-solvents [488,490]. To avoid drug precipitation, SMEDDS are supplied with hydrophilic polymers, such as polyvinylpyrrolidone and hydroxypropyl methylcellulose. The use of SMEDDSs significantly improved the stability, effectiveness, and Cmax and AUC values of curcumin, quercetin, and resveratrol [490].

Another way to improve the bioavailability of natural compounds is the application of phosphatidylcholine complexes, called “phytosomes” [487,488,491]. Their effectiveness has been demonstrated regarding silibinin. In prostate cancer patients, phytosomes were able to increase the Cmax of silibinin by up to 100 uM, with an average concentration of 1.2 uM at the end of the trial [492,493].

Phytosomes loaded with quercetin and scorpion venom peptides were able to target breast cancer cells [494]. Thymoquinone-loaded phytosomes exhibited cytotoxic effects in the lung cancer cell line [495].

Taken together, the application of a nanoparticle delivery system is considered as one of the most important ways to improve the bioavailability of herbal therapeutics (reviewed in [487,496,497]).

Piperine is a commonly used “bioenhancer” for many herbal products marketed in the USA [488,498]. This compound inhibits both CYP3A4 and P-glycoprotein. As reported, other inhibitors of CYP and UGT isoforms are α-mangostin, magnobol, peppermint oil, grapefruit juice (naringin), lysergol, chrysin, ginger extract, pterostilbene, silybin, gallic acid ester, genestein, and others (reviewed in [499]).

However, as stated in the previous subsection, one more option to address the challenge with bioavailability is to use herbal and mushroom extracts where a mixture of naturally co-occurring constituents promote the bioavailability and strong pharmacological properties of active compounds.

### 6.2. Safety

Undoubtedly, two key advantages of modern western medicine are the known profiles of efficacy and safety. International agencies including the Food and Drug Administration (FDA) and the Europe Medicine Agency (EMA) require at least one trail with control phase III significant results to launch a substance into clinics [500]. However, in some cases, drugs which are not approved by the FDA and EMS can be registered in certain countries.

There is a widespread belief that herbal medicine is safe and non-toxic. Despite the fact that herbal medicines are widely considered to be of a lower risk compared to synthetic drugs, they are not completely free from the possibility of toxicity or adverse effects. Thus, herbal and mushroom pharmacological products should be accurately and exhaustively managed.

Several reasons for the unsafety of herbal and mushroom medicine can be recognized: “intrinsic” and “external” toxicities, wrong indication, and herb–drug interactions [501].

“Intrinsic” toxicity is determined by the toxicity of some plants and mushrooms at a normal therapeutic dosage or in overdose. Herbal extracts represent a mixture of dozens of constituents with multiple pharmacological properties. Moreover, active compounds in the form of natural extracts frequently display synergistic effects. As stated by Paracelsus, ‘’Everything is poison, everything is medicine; either effect is determined by the dose”. Even medical plants well-known for being safe for centuries may have serious adverse effects. For instance, it was recently reported that aloe–emodin and aloin—two principle active components of *Aloe vera*—may have hepato- and nephrotoxicity [109] and may even induce the Wnt/β-catenin pathway which may be associated with potential carcinogenesis [121]. Moreover, there are media stories (e.g., https://www.bbc.com/news/stories-45971416; accessed on 20 February 2022) and scientific reports [502,503,504] about serious hepatotoxicity in people who consumed excessive amounts of green tea or used its extract as a food supplement. The green-tea-induced hepatotoxicity occurs due to the excessive consumption of (−)-epigallocatechin-3-gallate (EGGG). Its consumption safety level was determined by the European Commission [503,505].

“External toxicity” is associated with the possible environmental pollution of herbal sources with heavy metals, pesticides, and poisons.

As herbal and mushroom extracts are composed of a complex mixture of biologically active constituents, their intake in parallel with the usage of conventional drugs may result in herbal–drug interactions. Herbal–drug interactions display the synergistic or additive actions of herbal products with conventional medications as a result of overlapping affinities for common receptor sites. They may affect different physiological processes (the induction and inhibition of drug-metabolizing enzymes and ABC transporters, the alteration of gastrointestinal functions, and the modulation of the effects of antipsychotic therapeutics) which needs to be taken into account (reviewed in [506]).

All of these issues are addressed by complex investigations and through the procedure of standardization of manufacturing, ranging from pharmacological studies on human physiology to the precise monitoring of the herbal source quality, as well as the quantification of active and marker compounds.

### 6.3. Standardization

Besides safety, there is another closely related problem. The chemical composition of plants and mushrooms may vary depending on the genetic background and growth conditions. A major source of distrust towards the use of plants in modern medicine is the impossibility of the full standardization of plant material.

Standardization refers to all the information and activities aimed at developing and establishing requirements and control to ensure minimum quantitative and qualitative variations of active biochemicals in a herbal product. This is archived through assurance practices applied to agricultural and manufacturing processes [507]. Thus, standardization guarantees the content of one or more active constituents and marker compounds. This is closely associated with both efficiency and safety. It includes the evaluation of chemical constituents present in a herbal drug. This may involve the quantification of individual compounds of interest or chemical groups (total phenolics, total triterpenic acids, total alkaloids, and tannins). Standardization may use multiple marker-based fingerprint profiles [508]. The step-by-step standardization procedure, from primary culturing to the finished herbal product, is described in another review [509].

Whether the substance is synthetic or natural, the standardized procedure of its preclinical studies should be followed. Recently, the FDA adopted an ICH guideline on the nonclinical evaluation of anticancer drugs, including 41 questions and answers aimed at providing additional clarity about oncology drug development [369].

## 7. Overcoming Limitations to Integrate Folk and Modern Medicine

To integrate folk and modern medicine, standardization is required to be highly developed. Although this is by far a difficult obstacle, there are well-known examples of successful standardization approaches.

China is an upper–middle-income country with the second largest world economy (https://www.worldbank.org/en/country/china/; accessed on 26 March 2022). However, in China, both western modern medicine and TCM are officially used today, alone or in combination. One of the reasons is that TCM has proven its effectiveness for 2000 years. Now, China’s government strongly supports TCM (in the form of CPM), exporting its products to different countries for trials and therapy, and setting up a research partnership with the big international pharmaceutical companies such as Novartis or Astrazeneca, displaying global ambitions [7] (http://www.news.cn/english/2021-10/03/c_1310224791.htm; accessed on 26 March 2022). The fears of western medicine are related both to the concern about the safety profile, and a possible reduction in the monopoly currently held by large pharmaceutical corporations. Various aspects such as economic and political components, fears, and real examples of insecurity (both related to efficacy and a lack of evidence in various clinical trials) intertwine and both contribute to and hinder TCM’s application in developed countries [8]. Nevertheless, TCM actively continues to develop its niche in the modern world’s pharmacology.

In China, the standardization of TCM was set as one priority area to become the standard specification of international traditional medicine, with a lot of TCM standards established [510]. Thus, as demonstrated by China, it is possible to improve traditional medicine like this.

Thus, the standardization of plants and for anticancer clinical trials is also possible. Standardized medical substances derived from herbal sources are applied in different regions of the world. For instance, there are drops, syrups, and tablets used against coughs, which are derived from various companies including Kodelak™ (Moscow, Russia), Herbion™ (Burlington, ON, Canada), Dabur Honitus™ (New Delhi, India), Dr. Müller Syrups™ (Hradec Králové, Czech Rebublic), and Naturactive™ (Boe, France), etc.

Moreover, there are standardized dietary supplements which are manufactured by large world-class companies such as Solgar™ (Leonia, NJ, USA), Himalaya™ (Bangalore, India), NOW™ (Bloomingdale, IL, USA), and others, in accordance with developed standardized protocols. Some of these supplements are consumed worldwide and are derived from plants and mushrooms with strong antineoplastic properties described in this review: *Silybum marianum* (thistle), *Withania somnifera* (Ashwagandha), *Plumbago zeylanica* (Chitrak), *Boswellia serrata* (Boswellia extract), *Curcuma longa* (turmeric-based supplements), *Panax gingseng* (different supplements), *Glyzzhiriza glabra* (licorice), *Hypericum perforatum* (St. John’s wort), *Zingiber officinale* (ginger), *Agaricus blazei* (Andosan™, Oslo, Norway), etc. A plausible experimental approach to test the antineoplastic therapeutic properties of these plants and mushrooms can be exerted by using the corresponding supplements in preclinical experiments on animal tumor models. The quality control, standardized constitution, and orally available form can create good opportunities to credibly evaluate their anticancer potential, safety, and other possible beneficial effects on health. It is important to analyze the potential synergy between such supplements and conventional anticancer therapeutics. Taking into account that the bioavailability of active compounds is usually several-fold higher in the form of a herbal or mushroom extract (due to the co-existing constituents), the usage of dietary supplements derived from standardized extracts is very promising.

As was reported earlier, a mixture of naturally co-occurring constituents promotes the bioavailability and strong pharmacological properties of active compounds. Thus, the use of plant and mushroom medical products derived from their standardized extracts may also significantly increase the bioavailability of active compounds without additional manipulations.

One more interesting approach to bring herbs and mushrooms into modern medicine is the concept of “medical food based on certain herbs and mushrooms” [511,512]. In theory, food supplementation with anticancer herbs and mushrooms (e.g., shiitake, reishi, etc.) may help to prevent and reduce tumor growth. Chen and colleagues fed mice with gastric cancer with six medical edible plants used in TCM and observed the suppression of neoplastic growth through several molecular mechanisms [512]. All of the plants used possess well-known anticancer properties.

This new approach seems to be promising but requires more experimental data to confirm its efficiency.

## 8. Conclusions and Future Perspectives

Summarizing the information discussed above, we would like to highlight several points that should help implement traditional herbal medicine in current medicine:

− To date, a lot of information about a number of plants and mushrooms, and their individual bioactive compounds with well-documented antitumor properties, has been accumulated. Their respective full-scale multi-level studies should be top priorities.
− Despite there being a lot of investigations on the anticancer properties of a certain plant using tumor cell models, only a limited number of studies have been carried out with implication of control non-tumor cell models and subsequent animal studies. As the next step, comprehensive studies on their effectiveness, toxicity to non-cancer cells, and animal tissues in various doses are required to authorize natural-derived extracts and individual compounds into the next pre-clinical or clinical investigation.
− Progress in standardization is highly required to transform anecdotal folk herbal medicine into modern molecular pharmacology with clear mechanisms of action. This process includes investments into big programs regarding investigations, monitoring, and certifications of manufacturing the final product.
− On the examples of etoposide, irino- and topotecan, vinorelbine, docetaxel, and omacetaxine, the development of semi-synthetic derivates of newly identified natural compounds with significant anticancer properties may improve their characteristics and lead to new antineoplastic drugs.
− The study of a synergistic interaction of isolated natural compounds and crude plant- and mushroom-derived extracts with widely used anticancer therapeutics should help define the right dosage and compatibility between the natural and synthetic therapeutics.
− Natural compounds may sensitize tumors for modern therapeutics and be effective in adjuvant and neoadjuvant therapy.
− There is a variety of standardized dietary supplements from plants and mushrooms with presumable antineoplastic properties produced by large world-class companies. The important approach is to test their antitumor potential using animal models, especially in combination with relevant modern therapeutics.
− The pharmacological effects of active compounds are much higher in herbal extracts than in pure compounds due to co-existing constituents which may provide the pharmacokinetic synergy during intestinal absorption and the ‘’first-path’’ metabolism.
− Folk medicine may point to certain plants or mushrooms with highly potent anti-cancer properties and bioactive compounds. Herewith, the cooperation between cancer researchers and ethnobotanists or ethnomedicine specialists can benefit the development of new therapeutics.

To conclude, a systematic approach in studying the traditional herbal medicine is required to successfully integrate this unique knowledge into modern molecular medicine. This combined knowledge that encompasses both the empirical and theoretical approaches may provide a window of opportunities to facilitate the development of new chemotherapeutic strategies to treat malignancies.

## Figures and Tables

**Table 1:** Some African plants and their compounds with anti-neoplastic activity.

Plant	Active Compounds	Effects	References
*Aristolochia ringens*	Triterpenes	Cytotoxic effects of root extract against several cancer cell lines, solid tumors, and leukemia models in vivo.	[74,75]
*Beilschmiedia acuta*	Flavonoids, saponins, alkaloids	Bark-derived extract inhibited proliferation and induced apoptosis in human leukemia CCRF-CEM cells, but was less toxic to human normal hepatocytes AML12 cells.	[76]
*Dorstenia psilurus*	Alkaloids, phenolic compounds, flavonoids	Cytotoxic and anti-proliferative effects in HeLa cancer cells; caspase 3/7 up-regulation and induction of apoptosis in HL-60 cells.	[77,78]
*Echinops giganteus*	Brominated oleanolide	Root extract inhibited proliferation and induced apoptosis in leukemia CCRF-CEM cells.	[79,80]
*Imperata cylindrica*	Saponins, flavonoids Antineoplastic activity: arundoin, daucosterol	Cytotoxicity against the panel of cancer cells. Root extracts induced apoptosis, increased caspase 3/7 activity, and significant down-regulated MMPs.	[77,81]
*Piper capense*	Alkaloids, polyphenols, saponins	Seeds extracts were cytotoxic for a number of cancer cell lines. Fruits extracts induced the shrinkage of tumor size in animal models by inhibiting the development of VM tubes and microvessel density.	[79,82]
*Polyscia fulva*	Anthocyanins, flavonoids, triterpenes, saponins	Roots extracted inhibited proliferation and induced apoptosis in leukemia CCRF-CEM cells via the enhanced production of ROS. It was significantly less toxic for human normal hepatocytes AML12 cells.	[76]

**Table 2:** Some South American plants and their compounds with anti-neoplastic activity.

Plant	Active Compounds	Effects	References
*Achyrocline satureioides*	Achyrobichalcone, 3-*O*-methylquercetin, and other flavonoids	In vitro cytotoxicity and apoptosis in human breast cancer cells; inhibition of c-MYC and ERK/JNK in glioma cell lines.	[82,131]
*Aloysia polystachya*	Flavonoids	In vitro apoptosis in human colorectal cancer cells, and a decrease in the percentage of cancer stem cells (CSCs). In vivo inhibition of tumor growth in non-toxic doses.	[132]
*Azorella glabra*	Mulinic acid, azorellane terpenoids	G0/G1 cell cycle arrest and apoptosis in AML cell lines. A slight decrease in the survival of non-tumor cells	[133]
*Ephedra chilensis*	Terpens and fatty acids	IC50 of non-polar extracts for one breast cancer and three colon cell lines was at the level of doxorubicin; in vitro cytotoxicity for normal colon epithelium cells was less than doxorubicin.	[134]
*Croton lechleri*	Taspine	Leaf extracts exhibited cytotoxic antiproliferative effects on HeLa and SK23 cells in vitro, and antitumor effects in mice in vivo; moderate toxicity to mice.	[135,136]
*Laetia corymbulosa*	Corymbulosins B, C, D, E, F, G	Bark extract exhibited cytotoxicity to panel of cancer cells.	[137]
*Lepidium meyenii*	macamide and macaene fractions	Macadamine displayed anticancer activities against multiple cancer cell lines.	[138]
*Leptocarpha rivularis*	Leptocarpin	Cytotoxic effects against several cancer cell lines.	[139]
*Passiflora alata*	Flavonoids and saponins	In vitro cytotoxicity against the set of cancer cell lines, and in vivo antitumor activity against sarcoma S180-bearing mice with low general toxicity.	[140]
*Thevetia peruviana*	Thevetiaflavone, and individual cardiac glycosides	Fruit extract exhibited cytostatic and cytotoxic effects in cancer cell lines with moderate toxicity to non-tumor cells.	[141]

**Table 3:** Some frequently used traditional Chine’s formulations for a cancer treatment.

Clinical Formulation	Composition	Type of Cancer	Effects	References
*Aidi Injection*	*Mylabris Phalerata, Astragalus Membranaceus, Panax Ginseng, Acanthopanax Senticosus*	Different solid tumors, gynecologic tumors	Suppression of proliferation, migration, invasion, angiogenesis, and metastasis. Decreased p-PI3K and Bcl-xL in liver cancer cells. Induction of apoptosis. *In Clinic*: improved overall survival, the quality of life, and the effectiveness of chemotherapy.	[182,183,184]
*Fufangkushen Injection*	*Sophora flavescens, Heterosmilacis Japonica*	Different solid tumors	Reduced proliferation, tumor growth, and TRPV1-ERK phosphorylation; decreased IFN-γ, IL-6, and KC levels in S-180 sarcoma. Induced apoptosis via up-regulation of caspase-3 and Fas in esophageal carcinoma. *In Clinic*: improved the quality of life and the effectiveness of chemotherapy.	[185,186]
*Kanglaite injection*	*Coix lacryma-jobi*	Non-small cell lung cancer, colorectal cancer, pancreatic cancer	In vitro suppressed cell growth and induced apoptosis via up-regulation of p53, Fas, and caspase-3. In vivo down-regulation of the PI3K/Akt/mTOR signaling pathway, and tumor growth suppression.	[187,188]
*Kushen injection*	*Sophorae Flavescentis, Radix, Smilacis Glabrae Rhixoma*	Different solid tumors	Immunomodulatory activity via regulation of macrophages and CD8+ T cells, TNFR1, NF-κB p65, and MAPK p38.	[189]
*Qing-Dai*	*Baphicacanthus cusia, Polygonum tinctorium, Isatis indigotica*	Acute promyelocytic leukemia	Down-regulation of NF-κB, Iap1, Iap2, Bcl-2, BCL-xL, cyclin D1, and c-Myc; inhibition of angiogenesis by reducing JAK/STAT3, VEGFR2, ERK 1/2, Ang-1, PDGFB, and MMP2. Immunomodulatory activity through impact on CD4+ CD25+ Treg cells.	[190]
*Tien-Hsien*	*Cordyceps sinensis, Oldenlandia diffusa, Indigo Pulverata Levis, Polyporus umbellatus, Radix Astragali, Panax ginseng, Solanum nigrum, Pogostemon cablin, Atractylodis Macrocephalae Rhizoma, Trichosanthes Radix, Clematis Radix, Margarite, Ligustrum lucidum, Glycyrrhizae Radix*	Acute promyelocytic leukemia, breast cancer	Down-regulation of DNMT1, cyclin A, cyclin B1, p-AKT, Bcl-2, Akt/mTOR, Stat3, and ERK; induction of p21, p15, and apoptosis. Suppression of angiogenesis, metastasis, and tumor growth. Radiosensitization and immunomodulatory activity.	[191,192,193]
*Zeng-Sheng-Ping*	*Sophora tonkinensis, Polygonum bistorta, Prunella vulgaris, Sonchus brachyotus, Dictamnus dasycarpus, Dioscorea bulbifera*	Alimentary tract cancer; oral, lung, and bladder cancer	Inhibition of EGFR and Notch signaling. *In Clinic*: slowed down tumor growth and increased overall survival.	[194,195,196]

**Table 4:** Some European plants and their compounds with anti-neoplastic activity.

Plant	Biologically Active Compounds	Effects	References
*Allium sativum*	Allicin, alliin, diallyl disulfide, diallyl trisulfide, *Z*-ajoene, *S*-allyl cysteine, *S*-propargyl-l-cysteine, *S*-allyl cysteine	Multiple anticancer effects and known molecular mechanisms of both crude extracts and individual compounds. Suppression of angiogenesis and migration in vivo.	[314]
*Arctium lappa*	Arctigenin, lappaol F, stigmasterol ß-sitosterol	Suppression of growth, invasion, and migration of cancer cell lines. Inhibition of hippo-signaling pathway. Reduction in tumor growth in vivo.	[315,316,317]
*Centaurea solstitialis*	Solstitialin A	Cytotoxic and cytostatic effects in a panel of cancer cell models	[276,318]
*Ebenus boissieri*		Antiproliferative and cytotoxic effects in human breast, cervical, and lung cancer cell lines. Induction of TNF-α expression.	[319,320,321]
*Rosmarinus officinalis*	Carnosol, carnosic acid, sageone, rosmarinic acid	Multiple antineoplastic effects in vitro and in vivo with known molecular mechanisms, including epigenetic regulation.	[322]
*Menyanthes trifoliata*	Betulinic acid, syringic acid, ellagic acid, rutin, chlorogenic acid	Cell cycle G2/M arrest and apoptosis in grade IV glioma. No toxicity to normal human astrocytes.	[323]
*Vitis vinifera*	Viniferin, resveratrol	Multiple anticancirogenic and antineoplastic effects with known molecular mechanisms.	[324,325]
*Viscum album*	Iscodor, helixor A, lectins (ML-I, ML-II, and ML-III), viscotoxins, polysaccharides, phenolic compounds	Cytostatic and cytotoxic effects in vitro and in vivo. Immunomodulatory activity and reduction in cancer-related fatigue in clinical studies. Helixor A and Iscador are used in Europe as adjuvants in cancer therapy.	[326,327]

**Table 5:** Some others North American plants and their compounds with anti-neoplastic activity.

Plant	Biologically Active Compounds	Effects	References
*Aristolochia foetida*	β-sitosterol, stigmasterol, and other compounds	Apoptosis in MCF-7 cancer cells with less toxicity to non-tumor cells.	[336]
*Asimina triloba*	Acetogenins	Extracts from different parts of plant suppressed proliferation; induced apoptosis in AGS and HeLa cells; inhibited inflammatory makers NO, TNF-α, IL-6, and iNOS (inducible nitric oxide synthase).	[337]
*Capraria biflora*	Biflorin	Different anticancer effects in a number of tumor cell lines. Inhibition of c-MYC expression.	[338,339]
*Echinacea purpurea*	Echinacoside, alkylamides	Reduced proliferation, increased level of ROS, caspase 3 activity, and apoptosis in human lung cells. Inhibition of Wnt/β-catenin pathway. Immunomodulatory activity in vivo.	[340,341]
*Sanguinaria canadensis*	Sanguinarine, chelerythrine, berberine	Cytotoxic and antiproliferative effects in melanoma and child ALL cell lines. Induction of apoptosis by cIAP1, cIAP2, and XIAP suppression in pre-ALL cell lines. Sanguinarine and berberine binds G-quadruplex in oncogenes and telomeres.	[342,343,344]

**Table 6:** Some other Australian plants and their compounds with anti-neoplastic activity.

Plant	Active Compounds	Effects	References
*Terminalia ferdinandiana*	Tannins, flavonoids: gallic acid, ellagic acid, lutein, hesperitin, kaempferol, luteolin, and quercetin	Antiproliferative and proapoptotic activity in cancer cell lines. No toxicity to human dermal fibroblasts and shrimp *Artemia franciscana* bioassay.	[348,349]
*Tasmannia lanceolata*	Phenolic acids: coumaric acid, chlorogenic acid. Flavonoids: quercetin, quercetin 3-rutinoside, and anthocyanin (cyanidin 3-rutinoside)	Cytotoxicity to different cancer cell models with non-significant effects on normal colon, stomach, and intestine cells.	[350]
*Davidsonia pruriens*	Anthocyanin compounds, flavanoids	Significant cytotoxicity to a panel of cancer cell models and low toxicity in the *Artemia nauplii* bioassay.	[351,352]
*Elaeocarpus angustifolius*	Not identified	Significant cytotoxicity to Hela and Caco-2 cell models and low toxicity in the *Artemia nauplii* bioassay.	[352]
*Pittosporum angustifolium*	Alcaloids, saponins	Antiproliferative effects of 7 saponins with IC50 values in a range of 1.74–34.1 µM for MCF7, HaCaT, LN18, and 5637 cancer cells.	[353]

**Table 7:** Others mushrooms and their compounds with strong anti-neoplastic activity.

Mushroom	Active Compounds	Effects	References
*Agaricus subrufescens* (“mushroom of the sun”)	β-glucans (β-(1–3) linked backbone with (1–6) linked side branches); ergosterol	Pre-clinic: various immune stimulatory response. Clinic: increased activity of natural killer (NK) cells, and improved quality of life; increased number of plasmacytoid dendritic cells (DCs), Tregs, IL-5, and IL-7 in the blood.	[470,471,472]
*Phellinus linteus*	Polysaccharides, hispolon, phellinulin A, atractylenolide I, phellifuropyranone, meshimakobnol A, and meshimakobnol B	Pre-clinic: down-regulation of PI3K/AKT, ERK1/2, NF-kB Snail and Twist, cyclin D1 and -E, MMP-2 and -9, TGF-α; increased p53, p21, p27, and Bax; suppression of pancreatic CSCs. In clinic: Disease-free and overall survival of pancreatic cancer patients after tumor resection.	[469,473,474]
*Hericium erinaceus* (Lion’s mane)	4-chloro-3,5-dimethoxybenzoic methyl ester, erinacine A, herierin III, herierin IV, and erinacerin G	The epigenetic regulation of FasL and TRAIL; sustained phosphorylation of FAK/AKT/p70S6K and the PAK1 pathways; generation of ROS; apoptosis via activation of JNK, p300, and NFκB p50; increased expression of TNFR, Fas, and FasL.	[475,476,477,478]
*Trametes versicolor* (Turkey tail)	Protein polysaccharide beta-glucan β-1,4 main chain with β-1,3 and β-1,6 side chains (PSKs)	In clinic: PSK, including adjuvant immunochemotherapy, significantly prolonged 5-year survival and disease-free rate for patients with gastric and colorectal cancer; slows progression of advanced non-small cell lung cancer.	[479,480]

**Table 8:** Selected plants, mushrooms, and their active compounds in the order of their priority to study anticancer capabilities. The priority was suggested based on the available information about the anti-neoplastic efficacy and safety in preclinical and clinical studies.

**High Priority**		
**Plants**	**Mushrooms**	**Individual Compounds**
*Cephalotaxus harringtonia, Oldenlandia diffusa, Scutellaria barbata, Curcuma longa, Xanthium* ssp., *Zingiber officinalis, Hypericum perforatum, Glycyrrhiza glabra, Silybum marianum, Panax americanus and P. ginseng, Aloe vera and A. arborescence, Tabebuia impetiginosa, Viscum album, Allium sativum, Vitis vinifera, Rosmarinus officinalis, Echinacea purpurea, Sanguinaria canadensis*	*Lentinula edodes (Shiitake), Ganoderma lucidum (Reishi), Grifola frondosa (Maitake),* *Cordyceps sinensis and C. militaris, Agaricus blazei, Trametes versicolor, Phellinus linteus*	Quercetin, kaempferol, ginsenosides (especially Rg3), silibinin, isoliquiritigenin, (−)- epicatechin, oleanolic acid, ursolic acid, hyperforin, hypericin, xanthatin, curcumin, withaferin A, withanone, scutellarein, scutellarin, homoharringtonine and its semi-synthetic derivates, chlorogenic acid, caffeic acid, carnosol, rosmarinic acid, resveratrol, iscodor, helixor A, shogaol, boswellic acids, hispolon, lentinan, cordycepin, echinacoside, and myricetin
**Secondary priority**		
*Betula pubescens, Eremophila galeata, Combretum caffrum, Acacia nilotica, Guera senegalis, Tasmannia lanceolata, Davidsonia pruriens,* *Elaeocarpus angustifolius, Pittosporum angustifolium, Terminalia ferdinandiana, Aristolochia ringens, Beilschmiedia acuta, Dorstenia psilurus, Aristolochia ringens, Beilschmiedia acuta, Dorstenia psilurus, Echinops giganteus, Imperata cylindrica, Piper capense, Polyscia fulva, Achyrocline satureioides, Aloysia polystachya, Azorella glabra, Ephedra chilensis, Croton lechleri, Laetia corymbulosa, Lepidium meyenii, Leptocarpha rivularis, Passiflora alata, Thevetia peruviana, Menyanthes trifoliata, Ebenus boissieri, Centaurea solstitialis, Arctium lappa, Capraria biflora, Asimina triloba, Aristolochia foetida*	*Hericium erinaceus* (*Lion’s mane), Inonotus obliquus* (*Chaga)*	Gallic acid, combrestastatins, pyrogallol, betulinic acid, guieranone B, harringtonine, isoharringtonine, and doxyharringtonine, aloe-emodin, aloins, leptocarpin, macamide and macaene, corymbulosins, taspine, mulinic acid, achyrobichalcone, 3-O-methylquercetin, arctigenin, lappaol F, solstitialin A, sageone, biflorin, acetogenins, β-sitosterol, stigmasterol, sanguinarine, gandoderic acid, grifolin, illudin-S, lapachol and β-lapachone, carthamidin, carnosic acid, hispidin, inotodiol, syringic acid, p-coumaric acid, caffeoyl quinic acids, viniferin, lectins (ML-I, ML-II, and ML-III) from Viscum album

## Data Availability

Data sharing not applicable.

## Supplementary Materials

The following supporting information can be downloaded at: https://www.mdpi.com/article/10.3390/ph15070868/s1, Figure S1: chemical structures of compounds with anti-neoplastic properties and their sources; Table S1: the actual list of anticancer drugs derived from live organisms (https://www.cancer.gov/about-cancer/treatment/drugs; accessed on 1 March 2022); Table S2: the actual list of chemotherapeutic regimes including drugs derived from live organisms (https://www.cancer.gov/about-cancer/treatment/drugs; accessed on 1 March 2022).

## References

[1] WHO (2021). CureAll Framework: WHO Global Initiative for Childhood Cancer: Increasing Access, Advancing Quality, Saving Lives.

[2] Garodia P., Ichikawa H., Malani N., Sethi G., Aggarwal B.B. (2007). From ancient medicine to modern medicine: Ayurvedic concepts of health and their role in inflammation and cancer. J. Soc. Integr. Oncol..

[3] Mann M., Pathak S.R. (2018). Ayurveda: A new dimension in the era of modern medicine. Synthesis of Medicinal Agents from Plants.

[4] Vaghora B., Shukla V. (2016). Impact of different phytochemical classes and Ayurvedic plants in battle against cancer. Skin.

[5] Wu M., Lu P., Shi L., Li S. (2015). Traditional Chinese patent medicines for cancer treatment in China: A nationwide medical insurance data analysis. Oncotarget.

[6] Zhang N., Shi N., Li S., Liu G., Han Y., Liu L., Zhang X., Kong X., Zhang B., Yuan W. (2020). A Retrospective Study on the Use of Chinese Patent Medicine in 24 Medical Institutions for COVID-19 in China. Front. Pharmacol..

[7] Ovais M., Khalil A.T., Jan S.A., Ayaz M., Ullah I., Shinwari W., Shinwari Z.K. (2019). Traditional Chinese Medicine Going Global: Opportunities for Belt and Road Countries: TCM Importance in the Context of Belt Road Initiative. Proc. Pak. Acad. Sci. B Life Environ. Sci..

[8] Cyranoski D. (2018). Why Chinese medicine is heading for clinics around the world. Nature.

[9] Katayama K., Yoshino T., Munakata K., Yamaguchi R., Imoto S., Miyano S., Watanabe K. (2013). Prescription of kampo drugs in the Japanese health care insurance program. Evid. Based Complement. Altern. Med..

[10] Motoo Y., Cameron S. (2022). Kampo medicines for supportive care of patients with cancer: A brief review. Integr. Med. Res..

[11] Shimizu M., Takayama S., Kikuchi A., Arita R., Ono R., Ishizawa K., Ishii T. (2021). Kampo medicine treatment for advanced pancreatic cancer: A case series. Front. Nutr..

[12] Takayama S., Tomita N., Arita R., Ono R., Kikuchi A., Ishii T. (2020). Kampo Medicine for various aging-related symptoms: A review of geriatric syndrome. Front. Nutr..

[13] Yamakawa J.-i., Motoo Y., Moriya J., Ogawa M., Uenishi H., Akazawa S., Sasagawa T., Nishio M., Kobayashi J. (2013). Role of Kampo medicine in integrative cancer therapy. Evid. Based Complement. Altern. Med..

[14] Watanabe K., Matsuura K., Gao P., Hottenbacher L., Tokunaga H., Nishimura K., Imazu Y., Reissenweber H., Witt C.M. (2011). Traditional Japanese Kampo medicine: Clinical research between modernity and traditional medicine—The state of research and methodological suggestions for the future. Evid. Based Complement. Altern. Med..

[15] Ito A., Munakata K., Imazu Y., Watanabe K. (2012). First nationwide attitude survey of Japanese physicians on the use of traditional Japanese medicine (Kampo) in cancer treatment. Evid. Based Complement. Altern. Med..

[16] Aoyama T., Tamagawa H. (2020). The clinical effect of Kampo medicine in multimodal treatment for Gastrointestinal Cancer in Japan. J. Cancer.

[17] Rondilla N.A., Rocha I.C.N., Roque S.J., Lu R.M., Apolinar N.L.B., Solaiman-Balt A.A., Abion T.J., Banatin P.B., Javier C.V. (2021). Folk Medicine in the Philippines: A Phenomenological Study of Health-Seeking Individuals. Int. J. Med. Stud..

[18] Arnason J., Cal V., Pesek T., Awad R., Bourbonnais-Spear N., Collins S., Otarola-Rojas M., Walshe-Roussel B., Audet P., Ta C.A. (2022). A review of ethnobotany and ethnopharmacology of traditional medicines used by Q’eqchi’Maya Healers of Xna’ajeb’aj Ralch’o’och’, Belize. Botany.

[19] Jorim R.Y., Korape S., Legu W., Koch M., Barrows L.R., Matainaho T.K., Rai P.P. (2012). An ethnobotanical survey of medicinal plants used in the eastern highlands of Papua New Guinea. J. Ethnobiol. Ethnomed..

[20] Neergheen V.S., Kam A.H., Pem Y., Ramsaha S., Bahorun T. (2020). Regulation of cancer cell signaling pathways as key events for therapeutic relevance of edible and medicinal mushrooms. Proc. Semin. Cancer Biol..

[21] Shoaib A., Tabish M., Ali S., Arafah A., Wahab S., Almarshad F.M., Rashid S., Rehman M.U. (2021). Dietary phytochemicals in cancer signalling pathways: Role of miRNA targeting. Curr. Med. Chem..

[22] Oyenihi O.R., Oyenihi A.B., Erhabor J.O., Matsabisa M.G., Oguntibeju O.O. (2021). Unravelling the anticancer mechanisms of traditional herbal medicines with metabolomics. Molecules.

[23] Hanahan D., Weinberg R.A. (2011). Hallmarks of cancer: The next generation. Cell.

[24] Kontomanolis E.N., Koutras A., Syllaios A., Schizas D., Mastoraki A., Garmpis N., Diakosavvas M., Angelou K., Tsatsaris G., Pagkalos A. (2020). Role of oncogenes and tumor-suppressor genes in carcinogenesis: A review. Anticancer Res..

[25] Sanchez-Vega F., Mina M., Armenia J., Chatila W.K., Luna A., La K.C., Dimitriadoy S., Liu D.L., Kantheti H.S., Saghafinia S. (2018). Oncogenic signaling pathways in the cancer genome atlas. Cell.

[26] Witsch E., Sela M., Yarden Y. (2010). Roles for growth factors in cancer progression. Physiology.

[27] Patranabis S. (2021). Recent Advances in the Therapeutic Development of Receptor Tyrosine Kinases (RTK) against Different Types of Cancer. Protein Kinases—Promising Targets for Anticancer Drug Research.

[28] Zou Z., Tao T., Li H., Zhu X. (2020). mTOR signaling pathway and mTOR inhibitors in cancer: Progress and challenges. Cell Biosci..

[29] Yun C.W., Jeon J., Go G., Lee J.H., Lee S.H. (2020). The dual role of autophagy in cancer development and a therapeutic strategy for cancer by targeting autophagy. Int. J. Mol. Sci..

[30] Al-Masri M., Paliotti K., Tran R., Halaoui R., Lelarge V., Chatterjee S., Wang L.-T., Moraes C., McCaffrey L. (2021). Architectural control of metabolic plasticity in epithelial cancer cells. Commun. Biol..

[31] Shuvalov O., Petukhov A., Daks A., Fedorova O., Vasileva E., Barlev N.A. (2017). One-carbon metabolism and nucleotide biosynthesis as attractive targets for anticancer therapy. Oncotarget.

[32] Shuvalov O., Daks A., Fedorova O., Petukhov A., Barlev N. (2021). Linking metabolic reprogramming, plasticity and tumor progression. Cancers.

[33] Daks A., Shuvalov O., Fedorova O., Petukhov A., Lezina L., Zharova A., Baidyuk E., Khudiakov A., Barlev N.A. (2021). p53-Independent Effects of Set7/9 Lysine Methyltransferase on Metabolism of Non-Small Cell Lung Cancer Cells. Front. Oncol..

[34] Munshi P.N., Lubin M., Bertino J.R. (2014). 6-thioguanine: A drug with unrealized potential for cancer therapy. Oncologist.

[35] Morgunkova A., Barlev N.A. (2006). Lysine methylation goes global. Cell Cycle.

[36] Cao J., Yan Q. (2020). Cancer epigenetics, tumor immunity, and immunotherapy. Trends Cancer.

[37] Cheng Y., He C., Wang M., Ma X., Mo F., Yang S., Han J., Wei X. (2019). Targeting epigenetic regulators for cancer therapy: Mechanisms and advances in clinical trials. Signal Transduct. Target. Ther..

[38] Daks A., Vasileva E., Fedorova O., Shuvalov O., Barlev N.A. (2022). The Role of Lysine Methyltransferase SET7/9 in Proliferation and Cell Stress Response. Life.

[39] Vasileva E., Shuvalov O., Petukhov A., Fedorova O., Daks A., Nader R., Barlev N. (2020). KMT Set7/9 is a new regulator of Sam68 STAR-protein. Biochem. Biophys. Res. Commun..

[40] Pottier C., Fresnais M., Gilon M., Jérusalem G., Longuespée R., Sounni N.E. (2020). Tyrosine kinase inhibitors in cancer: Breakthrough and challenges of targeted therapy. Cancers.

[41] Zhang M., Zhang L., Hei R., Li X., Cai H., Wu X., Zheng Q., Cai C. (2021). CDK inhibitors in cancer therapy, an overview of recent development. Am. J. Cancer Res..

[42] Mukherjee N., Amato C.M., Skees J., Todd K.J., Lambert K.A., Robinson W.A., Van Gulick R., Weight R.M., Dart C.R., Tobin R.P. (2020). Simultaneously inhibiting BCL2 and MCL1 is a therapeutic option for patients with advanced melanoma. Cancers.

[43] He P., Qiu K., Jia Y. (2018). Modeling of mesenchymal hybrid epithelial state and phenotypic transitions in EMT and MET processes of cancer cells. Sci. Rep..

[44] Bhatia S., Wang P., Toh A., Thompson E.W. (2020). New insights into the role of phenotypic plasticity and EMT in driving cancer progression. Front. Mol. Biosci..

[45] Yu Z., Pestell T.G., Lisanti M.P., Pestell R.G. (2012). Cancer stem cells. Int. J. Biochem. Cell Biol..

[46] Peitzsch C., Tyutyunnykova A., Pantel K., Dubrovska A. (2017). Cancer stem cells: The root of tumor recurrence and metastases. Proc. Semin. Cancer Biol..

[47] Ermakov A., Daks A., Fedorova O., Shuvalov O., Barlev N.A. (2018). Ca2^+^-depended signaling pathways regulate self-renewal and pluripotency of stem cells. Cell Biol. Int..

[48] Waldman A.D., Fritz J.M., Lenardo M.J. (2020). A guide to cancer immunotherapy: From T cell basic science to clinical practice. Nature Rev. Immunol..

[49] Smirnov S., Petukhov A., Levchuk K., Kulemzin S., Staliarova A., Lepik K., Shuvalov O., Zaritskey A., Daks A., Fedorova O. (2021). Strategies to Circumvent the Side-Effects of Immunotherapy Using Allogeneic CAR-T Cells and Boost Its Efficacy: Results of Recent Clinical Trials. Front. Immunol..

[50] Alves-Silva J.M., Romane A., Efferth T., Salgueiro L. (2017). North African medicinal plants traditionally used in cancer therapy. Front. Pharmacol..

[51] Mbaveng A.T., Kuete V., Efferth T. (2017). Potential of Central, Eastern and Western Africa medicinal plants for cancer therapy: Spotlight on resistant cells and molecular targets. Front. Pharmacol..

[52] Klopper R.R., Gautier L., Chatelain C., Smith G.F., Spichiger R. (2007). Floristics of the angiosperm flora of Sub-Saharan Africa: An analysis of the African Plant Checklist and Database. Taxon.

[53] Linder H.P. (2014). The evolution of African plant diversity. Front. Ecol. Evolut..

[54] Rather L.J., Mohammad F. (2015). *Acacia nilotica* (L.): A review of its traditional uses, phytochemistry, and pharmacology. Sustain. Chem. Pharm..

[55] Malami I., Jagaba N.M., Abubakar I.B., Muhammad A., Alhassan A.M., Waziri P.M., Yahaya I.Z.Y., Mshelia H.E., Mathias S.N. (2020). Integration of medicinal plants into the traditional system of medicine for the treatment of cancer in Sokoto State, Nigeria. Heliyon.

[56] Barapatre A., Meena A.S., Mekala S., Das A., Jha H. (2016). In vitro evaluation of antioxidant and cytotoxic activities of lignin fractions extracted from *Acacia nilotica*. Int. J. Biol. Macromol..

[57] Zheleva-Dimitrova D., Sinan K.I., Etienne O.K., Ak G., Sharmeen J.B., Dervisoglu G., Ozdemir F.A., Mahomoodally M.F., Zengin G. (2021). Comprehensive chemical characterization and biological evaluation of two Acacia species: *A. nilotica* and *A. ataxacantha*. Food Chem. Toxicol..

[58] Revathi S., Hakkim F.L., Kumar N.R., Bakshi H.A., Rashan L., Al-Buloshi M., Hasson S.S., Krishnan M., Javid F., Nagarajan K. (2018). Induction of HT-29 colon cancer cells apoptosis by Pyrogallol with growth inhibiting efficacy against drug-resistant *Helicobacter pylori*. Anticancer Agents Med. Chem..

[59] Sakthive K., Kannan N., Angeline A., Guruvayoorappan C. (2012). Anticancer activity of *Acacia nilotica* (L.) Wild. Ex. Delile subsp. indica against Dalton’s ascitic lymphoma induced solid and ascitic tumor model. Asian Pac. J. Cancer Prev..

[60] Revathi S., Hakkim F.L., Kumar N.R., Bakshi H.A., Sangilimuthu A.Y., Tambuwala M.M., Changez M., Nasef M.M., Krishnan M., Kayalvizhi N. (2019). In vivo anti cancer potential of pyrogallol in murine model of colon cancer. Asian Pac. J. Cancer Prev..

[61] Al-Nour M.Y., Ibrahim M.M., Elsaman T. (2019). Ellagic acid, Kaempferol, and Quercetin from *Acacia nilotica*: Promising combined drug with multiple mechanisms of action. Curr. Pharmacol. Rep..

[62] Thiagarajan K., Mohan S., Roy T.K., Chandrasekaran R. (2020). Antiproliferative effect of *Acacia nilotica* (L.) leaf extract rich in ethyl gallate against human carcinoma cell line KB. Indian J. Pharmacol..

[63] Dirar A.I., Devkota H.P. (2021). Ethnopharmacological uses, phytochemistry and pharmacological activities of *Guiera senegalensis* JF Gmel. (*Combretaceae*). J. Ethnopharmacol..

[64] Adebayo I.A., Gagman H.A., Balogun W.G., Adam M.A.A., Abas R., Hakeem K.R., Nik Him N.A.I.I.B., Samian M.R.B., Arsad H. (2019). *Detarium microcarpum*, *Guiera senegalensis*, and *Cassia siamea* induce apoptosis and cell cycle arrest and inhibit metastasis on MCF7 breast cancer cells. Evid. Based Complement. Altern. Med..

[65] Kuete V., Eichhorn T., Wiench B., Krusche B., Efferth T. (2012). Cytotoxicity, anti-angiogenic, apoptotic effects and transcript profiling of a naturally occurring naphthyl butenone, guieranone A. Cell Div..

[66] Bello B.A., Khan S.A., Khan J.A., Syed F.Q., Anwar Y., Khan S.B. (2017). Antiproliferation and antibacterial effect of biosynthesized AgNps from leaves extract of *Guiera senegalensis* and its catalytic reduction on some persistent organic pollutants. J. Photochem. Photobiol. B Biol..

[67] Singh R., Kaur H. (2009). Advances in synthetic approaches for the preparation of combretastatin-based anti-cancer agents. Synthesis.

[68] Karatoprak G.Ş., Küpeli Akkol E., Genç Y., Bardakcı H., Yücel Ç., Sobarzo-Sánchez E. (2020). Combretastatins: An overview of structure, probable mechanisms of action and potential applications. Molecules.

[69] Nwankwo J. (2017). Anticancer potentials of phytochemicals from some indigenous food and medicinal plants of West Africa. Adv. Cancer Prev..

[70] Twilley D., Rademan S., Lall N. (2020). A review on traditionally used South African medicinal plants, their secondary metabolites and their potential development into anticancer agents. J. Ethnopharmacol..

[71] Sagbo I.J., Otang-Mbeng W. (2021). Plants used for the traditional management of cancer in the eastern cape province of south africa: A review of ethnobotanical surveys, ethnopharmacological studies and active phytochemicals. Molecules.

[72] Khorombi T., Fouché G., Kolesnikova N., Maharaj V., Nthambeleni R., Van der Merwe M.R. (2006). Investigation of South African plants for anti cancer properties. Pharmacology.

[73] Matowa P.R., Gundidza M., Gwanzura L., Nhachi C.F. (2020). A survey of ethnomedicinal plants used to treat cancer by traditional medicine practitioners in Zimbabwe. BMC Complement. Med. Ther..

[74] Akindele A.J., Wani Z., Mahajan G., Sharma S., Aigbe F.R., Satti N., Adeyemi O.O., Mondhe D.M. (2015). Anticancer activity of *Aristolochia ringens* Vahl. (Aristolochiaceae). J. Tradit. Complement. Med..

[75] Ahmad J., Ajani E., Sabiu S. (2021). Chemical group profiling, in vitro and in silico evaluation of *Aristolochia ringens* on α-amylase and α-glucosidase activity. Evid. Based Complement. Altern. Med..

[76] Kuete V., Tankeo S.B., Saeed M.E., Wiench B., Tane P., Efferth T. (2014). Cytotoxicity and modes of action of five Cameroonian medicinal plants against multi-factorial drug resistance of tumor cells. J. Ethnopharmacol..

[77] Somaida A., Tariq I., Ambreen G., Abdelsalam A.M., Ayoub A.M., Wojcik M., Dzoyem J.P., Bakowsky U. (2020). Potent cytotoxicity of four cameroonian plant extracts on different cancer cell lines. Pharmaceuticals.

[78] Pieme C.A., Guru S.K., Ambassa P., Kumar S., Ngameni B., Ngogang J.Y., Bhushan S., Saxena A.K. (2013). Induction of mitochondrial dependent apoptosis and cell cycle arrest in human promyelocytic leukemia HL-60 cells by an extract from *Dorstenia psilurus*: A spice from Cameroon. BMC Complement. Altern. Med..

[79] Kuete V., Sandjo L.P., Wiench B., Efferth T. (2013). Cytotoxicity and modes of action of four Cameroonian dietary spices ethno-medically used to treat cancers: *Echinops giganteus*, *Xylopia aethiopica*, *Imperata cylindrica* and *Piper capense*. J. EthnoPharmacol..

[80] Sandjo L.P., Kuete V., Siwe X.N., Poumale H.M., Efferth T. (2016). Cytotoxicity of an unprecedented brominated oleanolide and a new furoceramide from the *Cameroonian spice*, *Echinops giganteus*. Nat. Product Res..

[81] Jung Y.-K., Shin D. (2021). *Imperata cylindrica*: A review of phytochemistry, pharmacology, and industrial applications. Molecules.

[82] Wamba B.E., Ghosh P., Mbaveng A.T., Bhattacharya S., Debarpan M., Depanwita S., Saunak M.M., Kuete V., Murmu N. (2021). Botanical from *Piper capense* Fruit Can Help to Combat the Melanoma as Demonstrated by In Vitro and In Vivo Studies. Evid. Based Complement. Altern. Med..

[83] Ulloa Ulloa C., Acevedo-Rodríguez P., Beck S., Belgrano M.J., Bernal R., Berry P.E., Brako L., Celis M., Davidse G., Forzza R.C. (2017). An integrated assessment of the vascular plant species of the Americas. Science.

[84] Zhang J., Hunto S.T., Yang Y., Lee J., Cho J.Y. (2020). *Tabebuia impetiginosa*: A comprehensive review on Tradit. uses, phytochemistry, and immunopharmacological properties. Molecules.

[85] Warashina T., Nagatani Y., Noro T. (2004). Constituents from the bark of *Tabebuia impetiginosa*. PhytoChem..

[86] Mukherjee B., Telang N., Wong G. (2009). Growth inhibition of estrogen receptor positive human breast cancer cells by Taheebo from the inner bark of Tabebuia avellandae tree. Int. J. Mol. Med..

[87] Kee J.-Y., Han Y.-H., Park J., Kim D.-S., Mun J.-G., Ahn K.S., Kim H.-J., Um J.-Y., Hong S.-H. (2017). β-Lapachone inhibits lung metastasis of colorectal cancer by inducing apoptosis of CT26 cells. Integr. Cancer Ther..

[88] Woo H.J., Park K.-Y., Rhu C.-H., Lee W.H., Choi B.T., Kim G.Y., Park Y.-M., Choi Y.H. (2006). β-lapachone, a quinone isolated from Tabebuia avellanedae, induces apoptosis in HepG2 hepatoma cell line through induction of Bax and activation of caspase. J. Med. Food.

[89] Pires T.C., Dias M.I., Calhelha R.C., Carvalho A.M., Queiroz M.-J.R., Barros L., Ferreira I.C. (2015). Bioactive properties of *Tabebuia impetiginosa*-based phytopreparations and phytoformulations: A comparison between extracts and dietary supplements. Molecules.

[90] De Melo J.G., Santos A.G., de Amorim E.L.C., de Nascimento S.C., de Albuquerque U.P. (2011). Medicinal plants used as antitumor agents in Brazil: An ethnobotanical approach. Evid. Based Complement. Altern. Med..

[91] Shankar Babu M., Mahanta S., Lakhter A.J., Hato T., Paul S., Naidu S.R. (2018). Lapachol inhibits glycolysis in cancer cells by targeting pyruvate kinase M2. PLoS ONE.

[92] Zahra K., Dey T., Mishra S.P., Pandey U. (2020). Pyruvate kinase M2 and cancer: The role of PKM2 in promoting tumorigenesis. Front. Oncol..

[93] Shuvalov O., Kizenko A., Petukhov A., Fedorova O., Daks A., Bottrill A., Snezhkina A.V., Kudryavtseva A.V., Barlev N. (2020). SEMG1/2 augment energy metabolism of tumor cells. Cell Death Dis..

[94] Zhao W., Jiang L., Fang T., Fang F., Liu Y., Zhao Y., You Y., Zhou H., Su X., Wang J. (2021). β-Lapachone Selectively Kills Hepatocellular Carcinoma Cells by Targeting NQO1 to Induce Extensive DNA Damage and PARP1 Hyperactivation. Front. Oncol..

[95] Cheng M.-L., Lu Y.-F., Chen H., Shen Z.-Y., Liu J. (2015). Liver expression of Nrf2-related genes in different liver diseases. Hepatobiliary Pancreat. Dis. Int..

[96] Yang Y., Zheng J., Wang M., Zhang J., Tian T., Wang Z., Yuan S., Liu L., Zhu P., Gu F. (2021). NQO1 promotes an aggressive phenotype in hepatocellular carcinoma via amplifying ERK-NRF2 signaling. Cancer Sci..

[97] Hussain H., Green I.R. (2017). Lapachol and lapachone analogs: A journey of two decades of patent research (1997–2016). Exp. Opin. Ther. Pat..

[98] Rone A., Oliveira K.M., Guedes A.P., Dos Santos P.W., Aissa A.F., Batista A.A., Pavan F.R. (2021). A Novel Ruthenium (II) Complex with Lapachol Induces G2/M Phase Arrest through Aurora-B Kinase Down-Regulation and ROS-Mediated Apoptosis in Human Prostate Adenocarcinoma Cells. Front. Oncol..

[99] Chen Q., Bai L., Zhou X., Xu P., Li X., Xu H., Zheng Y., Zhao Y., Lu S., Xue M. (2020). Development of long-circulating lapachol nanoparticles: Formation, characterization, pharmacokinetics, distribution and cytotoxicity. RSC Adv..

[100] Beg M., Boothman D., Khosama L., Arriaga Y., Verma U., Sanjeeviaiah A., Kazmi S., Fattah F., Pilarski S., Rodriguez M. (2019). A phase I/Ib, multi-center trial of ARQ-761 (Beta-Lapachone) with gemcitabine/nab-paclitaxel in patients with advanced pancreatic cancer. Ann. Oncol..

[101] Tahara T., Watanabe A., Yutani M., Yamano Y., Sagara M., Nagai S., Saito K., Yamashita M., Ihara M., Iida A. (2020). STAT3 inhibitory activity of naphthoquinones isolated from Tabebuia avellanedae. Bioorg. Med. Chem..

[102] Rauwald H.W., Maucher R., Dannhardt G., Kuchta K. (2021). Dihydroisocoumarins, Naphthalenes, and Further Polyketides from *Aloe vera* and *A. plicatilis*: Isolation, Identification and Their 5-LOX/COX-1 Inhibiting Potency. Molecules.

[103] Huang P.-H., Huang C.-Y., Chen M.-C., Lee Y.-T., Yue C.-H., Wang H.-Y., Lin H. (2013). Emodin and aloe-emodin suppress breast cancer cell proliferation through ERα inhibition. Evid. Based Complement. Altern. Med..

[104] Wang Z., Tang T., Wang S., Cai T., Tao H., Zhang Q., Qi S., Qi Z. (2020). Aloin inhibits the proliferation and migration of gastric cancer cells by regulating NOX2–ROS-mediated pro-survival signal pathways. Drug Des. Dev. Ther..

[105] Sun R., Zhai R., Ma C., Miao W. (2020). Combination of aloin and metformin enhances the antitumor effect by inhibiting the growth and invasion and inducing apoptosis and autophagy in hepatocellular carcinoma through PI3K/AKT/mTOR pathway. Cancer Med..

[106] Pan Q., Pan H., Lou H., Xu Y., Tian L. (2013). Inhibition of the angiogenesis and growth of Aloin in human colorectal cancer in vitro and in vivo. Cancer Cell Int..

[107] Sanders B., Ray A.M., Goldberg S., Clark T., McDaniel H.R., Atlas S.E., Farooqi A., Konefal J., Lages L.C., Lopez J. (2018). Anti-cancer effects of aloe-emodin: A systematic review. J. Clin. Transl. Res..

[108] Manirakiza A., Irakoze L., Manirakiza S. (2021). Aloe and its Effects on Cancer: A Narrative Literature Review. East Afr. Health Res. J..

[109] Dong X., Zeng Y., Liu Y., You L., Yin X., Fu J., Ni J. (2020). Aloe-emodin: A review of its pharmacology, toxicity, and pharmacokinetics. Phytother. Res..

[110] Cheng C., Dong W. (2018). Aloe-emodin induces endoplasmic reticulum stress-dependent apoptosis in colorectal cancer cells. Med. Sci. Monit. Int. Med. J. Exp. Clin. Res..

[111] Shen F., Ge C., Yuan P. (2020). Aloe-emodin induces autophagy and apoptotic cell death in non-small cell lung cancer cells via Akt/mTOR and MAPK signaling. Eur. J. Pharmacol..

[112] Wang S., Yan W.-W., He M., Wei D., Long Z.-J., Tao Y.-M. (2020). Aloe emodin inhibits telomerase activity in breast cancer cells: Transcriptional and enzymological mechanism. Pharmacol. Rep..

[113] Kosiol N., Juranek S., Brossart P., Heine A., Paeschke K. (2021). G-quadruplexes: A promising target for cancer therapy. Mol. Cancer.

[114] Das A., Dutta S. (2021). Binding Studies of Aloe-Active Compounds with G-Quadruplex Sequences. ACS Omega.

[115] Li P., Ren K., Liang Y., Liu J., Liang Z., Zhang Y. (2020). Aloin promotes cell apoptosis by targeting HMGB1-TLR4-ERK axis in human melanoma cells. EXCLI J..

[116] Tao H., Tang T., Wang S., Wang Z., Ma Y., Cai T., Cheng X., Qi S., Zhang Y., Qi Z. (2019). The molecular mechanisms of Aloin induce gastric cancer cells apoptosis by targeting High Mobility Group Box 1. Drug Des. Dev. Ther..

[117] Birari L., Wagh S., Patil K.R., Mahajan U.B., Unger B., Belemkar S., Goyal S.N., Ojha S., Patil C.R. (2020). Aloin alleviates doxorubicin-induced cardiotoxicity in rats by abrogating oxidative stress and pro-inflammatory cytokines. Cancer Chemother. Pharmacol..

[118] Lissoni P., Rovelli F., Brivio F., Zago R., Colciago M., Messina G., Mora A., Porro G. (2009). A randomized study of chemotherapy versus biochemotherapy with chemotherapy plus Aloe arborescens in patients with metastatic cancer. In Vivo.

[119] Lissoni P., Giani L., Zerbini S., Trabattoni P., Rovelli F. (1998). Biotherapy with the pineal immunomodulating hormone melatonin versus melatonin plus *Aloe vera* in untreatable advanced solid neoplasms. Nat. Immun..

[120] Damani M.R., Shah A.R., Karp C.L., Orlin S.E. (2015). Treatment of ocular surface squamous neoplasia with topical *Aloe vera* drops. Cornea.

[121] Peng C., Zhang W., Dai C., Li W., Shen X., Yuan Y., Yan L., Zhang W., Yao M. (2019). Study of the aqueous extract of *Aloe vera* and its two active components on the Wnt/β-catenin and Notch signaling pathways in colorectal cancer cells. J. Ethnopharmacol..

[122] Arul B., Kothai R. (2020). Anticancer effect of capsaicin and its analogues. Capsicum.

[123] Chapa-Oliver A.M., Mejía-Teniente L. (2016). Capsaicin: From plants to a cancer-suppressing agent. Molecules.

[124] Chakraborty S., Adhikary A., Mazumdar M., Mukherjee S., Bhattacharjee P., Guha D., Choudhuri T., Chattopadhyay S., Sa G., Sen A. (2014). Capsaicin-induced activation of p53-SMAR1 auto-regulatory loop down-regulates VEGF in non-small cell lung cancer to restrain angiogenesis. PLoS ONE.

[125] Dai N., Ye R., He Q., Guo P., Chen H., Zhang Q. (2018). Capsaicin and sorafenib combination treatment exerts synergistic anti-hepatocellular carcinoma activity by suppressing EGFR and PI3K/Akt/mTOR signaling. Oncol. Rep..

[126] Lin Y.-T., Wang H.-C., Hsu Y.-C., Cho C.-L., Yang M.-Y., Chien C.-Y. (2017). Capsaicin induces autophagy and apoptosis in human nasopharyngeal carcinoma cells by downregulating the PI3K/AKT/mTOR pathway. Int. J. Mol. Sci..

[127] Chang C.-F., Islam A., Liu P.-F., Zhan J.-H., Chueh P.J. (2020). Capsaicin acts through tNOX (ENOX2) to induce autophagic apoptosis in p53-mutated HSC-3 cells but autophagy in p53-functional SAS oral cancer cells. Am. J. Cancer Res..

[128] Friedman J.R., Richbart S.D., Merritt J.C., Brown K.C., Denning K.L., Tirona M.T., Valentovic M.A., Miles S.L., Dasgupta P. (2019). Capsaicinoids: Multiple effects on angiogenesis, invasion and metastasis in human cancers. Biomed. Pharmacother..

[129] Amantini C., Morelli M.B., Nabissi M., Cardinali C., Santoni M., Gismondi A., Santoni G. (2016). Capsaicin triggers autophagic cell survival which drives epithelial mesenchymal transition and chemoresistance in bladder cancer cells in an Hedgehog-dependent manner. Oncotarget.

[130] Fedorova O., Daks A., Parfenyev S., Shuvalov O., Netsvetay S., Vasileva J., Gudovich A., Golotin V., Semenov O., Petukhov A. (2022). Zeb1-mediated autophagy enhances resistance of breast cancer cells to genotoxic drugs. Biochem. Biophys. Res. Commun..

[131] de Souza P.O., Bianchi S.E., Figueiró F., Heimfarth L., Moresco K.S., Gonçalves R.M., Hoppe J.B., Klein C.P., Salbego C.G., Gelain D.P. (2018). Anticancer activity of flavonoids isolated from *Achyrocline satureioides* in gliomas cell lines. Toxicol. In Vitro.

[132] Soares Machado M., Palma A., Panelo L.C., Paz L.A., Rosa F., Lira M.C., Azurmendi P., Rubio M.F., Lenz G., Urtreger A.J. (2020). Extract from *Aloysia polystachya* induces the cell death of colorectal cancer stem cells. Nutr. Cancer.

[133] Lamorte D., Faraone I., Laurenzana I., Trino S., Russo D., Rai D.K., Armentano M.F., Musto P., Sgambato A., De Luca L. (2020). Advances in *Azorella glabra* Wedd. Extract research: In vitro antioxidant activity, antiproliferative effects on acute myeloid leukemia cells and bioactive compound characterization. Molecules.

[134] Mellado M., Soto M., Madrid A., Montenegro I., Jara-Gutiérrez C., Villena J., Werner E., Godoy P., Aguilar L.F. (2019). In vitro antioxidant and antiproliferative effect of the extracts of *Ephedra chilensis* K Presl aerial parts. BMC Complement. Altern. Med..

[135] Alonso-Castro A.J., Ortiz-Sánchez E., Domínguez F., López-Toledo G., Chávez M., de Jesús Ortiz-Tello A., García-Carrancá A. (2012). Antitumor effect of *Croton lechleri* mull. arg.(euphorbiaceae). J. Ethnopharmacol..

[136] Montopoli M., Bertin R., Chen Z., Bolcato J., Caparrotta L., Froldi G. (2012). *Croton lechleri* sap. and isolated alkaloid taspine exhibit inhibition against human melanoma SK23 and colon cancer HT29 cell lines. J. Ethnopharmacol..

[137] Suzuki A., Saito Y., Fukuyoshi S., Goto M., Miyake K., Newman D.J., O’Keefe B.R., Lee K.-H., Nakagawa-Goto K. (2017). Corymbulosins D–H, 2-Hydroxy-and 2-Oxo-clerodane Diterpenes from the Bark of *Laetia corymbulosa*. J. Nat. Prod..

[138] Fu L., Wei J., Gao Y., Chen R. (2021). Antioxidant and antitumoral activities of isolated macamide and macaene fractions from *Lepidium meyenii* (Maca). Talanta.

[139] Bosio C., Tomasoni G., Martínez R., Olea A.F., Carrasco H., Villena J. (2015). Cytotoxic and apoptotic effects of leptocarpin, a plant-derived sesquiterpene lactone, on human cancer cell lines. Chem. Biol. Interact..

[140] Amaral R.G., Gomes S.V., Andrade L.N., Dos Santos S.A., Severino P., de Albuquerque Júnior R.L., Souto E.B., Brandão G.C., Santos S.L., David J.M. (2020). Cytotoxic, antitumor and toxicological profile of *Passiflora alata* leaf extract. Molecules.

[141] Ramos-Silva A., Tavares-Carreón F., Figueroa M., la Torre-Zavala D., Gastelum-Arellanez A., Rodríguez-García A., Galán-Wong L.J., Avilés-Arnaut H. (2017). Anticancer potential of *Thevetia peruviana* fruit methanolic extract. BMC Complement. Altern. Med..

[142] Kantarjian H.M., Talpaz M., Santini V., Murgo A., Cheson B., O’Brien S.M. (2001). Homoharringtonine: History, current research, and future directions. Cancer.

[143] Kantarjian H.M., O’Brien S., Cortes J. (2013). Homoharringtonine/omacetaxine mepesuccinate: The long and winding road to food and drug administration approval. Clin. Lymphoma Myeloma Leuk..

[144] Xie M., Jiang Q., Li L., Zhu J., Zhu L., Zhou D., Zheng Y., Yang X., Zhu M., Sun J. (2016). HAG (homoharringtonine, cytarabine, G-CSF) regimen for the treatment of acute myeloid leukemia and myelodysplastic syndrome: A meta-analysis with 2,314 participants. PLoS ONE.

[145] Gu L.-F., Zhang W.-G., Wang F.-X., Cao X.-M., Chen Y.-X., He A.-L., Liu J., Ma X.-R. (2011). Low dose of homoharringtonine and cytarabine combined with granulocyte colony-stimulating factor priming on the outcome of relapsed or refractory acute myeloid leukemia. J. Cancer Res. Clin. Oncol..

[146] Winer E.S., DeAngelo D.J. (2018). A review of omacetaxine: A chronic myeloid leukemia treatment resurrected. Oncol. Ther..

[147] Chen X.-J., Zhang W.-N., Chen B., Xi W.-D., Lu Y., Huang J.-Y., Wang Y.-Y., Long J., Wu S.-F., Zhang Y.-X. (2019). Homoharringtonine deregulates MYC transcriptional expression by directly binding NF-κB repressing factor. Proc. Nat. Acad. Sci. USA.

[148] Tong H., Ren Y., Zhang F., Jin J. (2008). Homoharringtonine affects the JAK2-STAT5 signal pathway through alteration of protein tyrosine kinase phosphorylation in acute myeloid leukemia cells. Eur. J. Haematol..

[149] Han X., Zhang X., Wang Q., Wang L., Yu S. (2020). Antitumor potential of *Hedyotis diffusa* Willd: A systematic review of bioactive constituents and underlying molecular mechanisms. Biomed. Pharmacother..

[150] Lee Y.K., Bae K., Yoo H.-S., Cho S.-H. (2018). Benefit of adjuvant Tradit. herbal medicine with chemotherapy for resectable gastric cancer. Integr. Cancer Ther..

[151] Yeh Y.-C., Chen H.-Y., Yang S.-H., Lin Y.-H., Chiu J.-H., Lin Y.-H., Chen J.-L. (2014). *Hedyotis diffusa* combined with *Scutellaria barbata* are the core treatment of Chinese herbal medicine used for breast cancer patients: A population-based study. Evid. Based Complement. Altern. Med..

[152] Yang B., Wang N., Wang S., Li X., Zheng Y., Li M., Song J., Zhang F., Mei W., Lin Y. (2019). Network-pharmacology-based identification of caveolin-1 as a key target of *Oldenlandia diffusa* to suppress breast cancer metastasis. Biomed. Pharmacother..

[153] Chung T.-W., Choi H., Lee J.-M., Ha S.-H., Kwak C.-H., Abekura F., Park J.-Y., Chang Y.-C., Ha K.-T., Cho S.-H. (2017). *Oldenlandia diffusa* suppresses metastatic potential through inhibiting matrix metalloproteinase-9 and intercellular adhesion molecule-1 expression via p38 and ERK1/2 MAPK pathways and induces apoptosis in human breast cancer MCF-7 cells. J. Ethnopharmacol..

[154] Lu P.-H., Chen M.-B., Ji C., Li W.-T., Wei M.-X., Wu M.-H. (2016). Aqueous *Oldenlandia diffusa* extracts inhibits colorectal cancer cells via activating AMP-activated protein kinase signalings. Oncotarget.

[155] Chen Q., Rahman K., Wang S.-J., Zhou S., Zhang H. (2020). *Scutellaria barbata*: A review on chemical constituents, pharmacological activities and clinical applications. Curr. Pharm. Des..

[156] Feng X.M., Su X.L. (2019). Anticancer effect of ursolic acid via mitochondria-dependent pathways. Oncol. Lett..

[157] Guo W., Xu B., Wang X., Zheng B., Du J., Liu S. (2020). The analysis of the anti-tumor mechanism of ursolic acid using connectively map approach in breast cancer cells line MCF-7. Cancer Manag. Res..

[158] Liu T., Ma H., Shi W., Duan J., Wang Y., Zhang C., Li C., Lin J., Li S., Lv J. (2017). Inhibition of STAT3 signaling pathway by ursolic acid suppresses growth of hepatocellular carcinoma. Int. J. Oncol..

[159] Wang S., Chang X., Zhang J., Li J., Wang N., Yang B., Pan B., Zheng Y., Wang X., Ou H. (2021). Ursolic Acid Inhibits Breast Cancer Metastasis by Suppressing Glycolytic Metabolism via Activating SP1/Caveolin-1 Signaling. Front. Oncol..

[160] Lewinska A., Adamczyk-Grochala J., Kwasniewicz E., Deregowska A., Wnuk M. (2017). Ursolic acid-mediated changes in glycolytic pathway promote cytotoxic autophagy and apoptosis in phenotypically different breast cancer cells. Apoptosis.

[161] Wang J., Jiang Z., Xiang L., Li Y., Ou M., Yang X., Shao J., Lu Y., Lin L., Chen J. (2014). Synergism of ursolic acid derivative US597 with 2-deoxy-D-glucose to preferentially induce tumor cell death by dual-targeting of apoptosis and glycolysis. Sci. Rep..

[162] Tang Z.-Y., Li Y., Tang Y.-T., Ma X.-D., Tang Z.-Y. (2022). Anticancer activity of oleanolic acid and its derivatives: Recent advances in evidence, target profiling and mechanisms of action. Biomed. Pharmacother..

[163] Li Y., Xu Q., Yang W., Wu T., Lu X. (2019). Oleanolic acid reduces aerobic glycolysis-associated proliferation by inhibiting yes-associated protein in gastric cancer cells. Gene.

[164] Lee J.-H., Yoo E.-S., Han S.-H., Jung G.-H., Han E.-J., Jung S.-H., Kim B.S., Cho S.-D., Nam J.-S., Choi C. (2021). Oleanolic acid induces apoptosis and autophagy via the PI3K/AKT/mTOR pathway in AGS human gastric cancer cells. J. Funct. Foods.

[165] Nie H., Wang Y., Qin Y., Gong X.G. (2016). Oleanolic acid induces autophagic death in human gastric cancer cells in vitro and in vivo. Cell Biol. Int..

[166] Xiaofei J., Mingqing S., Miao S., Yizhen Y., Shuang Z., Qinhua X., Kai Z. (2021). Oleanolic acid inhibits cervical cancer Hela cell proliferation through modulation of the ACSL4 ferroptosis signaling pathway. Biochem. Biophys. Res. Commun..

[167] Li F., Wang S., Niu M. (2021). Scutellarin inhibits the growth and EMT of gastric cancer cells through regulating PTEN/PI3K pathway. Biol. Pharm. Bull..

[168] Niu G., Sun L., Pei Y., Wang D. (2018). Oleanolic acid inhibits colorectal cancer angiogenesis by blocking the VEGFR2 signaling pathway. Anti Cancer Agents Med. Chem. Formerly Curr. Med. Chem. Anti-Cancer Agents.

[169] Ma T.-T., Zhang G.-L., Dai C.-F., Zhang B.-R., Cao K.-X., Wang C.-G., Yang G.-W., Wang X.-M. (2020). *Scutellaria barbata* and *Hedyotis diffusa* herb pair for breast cancer treatment: Potential mechanism based on network pharmacology. J. Ethnopharmacol..

[170] Gao J., Yin W., Corcoran O. (2019). From *Scutellaria barbata* to BZL101 in cancer patients: Phytochemistry, pharmacology, and clinical evidence. Nat. Prod. Commun..

[171] Chen V., Staub R.E., Fong S., Tagliaferri M., Cohen I., Shtivelman E. (2012). Bezielle selectively targets mitochondria of cancer cells to inhibit glycolysis and OXPHOS. PLoS ONE.

[172] Lang X., Chen Z., Yang X., Yan Q., Xu M., Liu W., He Q., Zhang Y., Cheng W., Zhao W. (2021). Scutellarein induces apoptosis and inhibits proliferation, migration, and invasion in ovarian cancer via inhibition of EZH2/FOXO1 signaling. J. Biochem. Mol. Toxicol..

[173] Ha S.E., Kim S.M., Vetrivel P., Kim H.H., Bhosale P.B., Heo J.D., Lee H.J., Kim G.S. (2021). Inhibition of cell proliferation and metastasis by scutellarein regulating PI3K/Akt/NF-κB signaling through PTEN activation in hepatocellular carcinoma. Int. J. Mol. Sci..

[174] Zeng S., Chen L., Sun Q., Zhao H., Yang H., Ren S., Liu M., Meng X., Xu H. (2021). Scutellarin ameliorates colitis-associated colorectal cancer by suppressing Wnt/β-catenin signaling cascade. Eur. J. Pharmacol..

[175] Jiang Q., Li Q., Chen H., Shen A., Cai Q., Lin J., Peng J. (2015). *Scutellaria barbata* D. Don inhibits growth and induces apoptosis by suppressing IL-6-inducible STAT3 pathway activation in human colorectal cancer cells. Exp. Ther. Med..

[176] Wei L.-H., Lin J.-M., Chu J.-F., Chen H.-W., Li Q.-Y., Peng J. (2017). *Scutellaria barbata* D. Don inhibits colorectal cancer growth via suppression of Wnt/β-catenin signaling pathway. Chin. J. Integr. Med..

[177] Lin J., Feng J., Yang H., Yan Z., Li Q., Wei L., Lai Z., Jin Y., Peng J. (2017). *Scutellaria barbata* D. Don inhibits 5-fluorouracil resistance in colorectal cancer by regulating PI3K/AKT pathway. Oncol. Rep..

[178] Chen F., Zhong Z., Tan H.Y., Guo W., Zhang C., Tan C.-W., Li S., Wang N., Feng Y. (2020). Uncovering the anticancer mechanisms of Chin. herbal medicine formulas: Ther. alternatives for liver cancer. Front. Pharmacol..

[179] Fan Y., Ma Z., Zhao L., Wang W., Gao M., Jia X., Ouyang H., He J. (2020). Anti-tumor activities and mechanisms of Traditional Chinese medicines formulas: A review. Biomed. Pharmacother..

[180] Wang K., Chen Q., Shao Y., Yin S., Liu C., Liu Y., Wang R., Wang T., Qiu Y., Yu H. (2021). Anticancer activities of TCM and their active components against tumor metastasis. Biomed. Pharmacother..

[181] Liu S.-H., Chen P.-S., Huang C.-C., Hung Y.-T., Lee M.-Y., Lin W.-H., Lin Y.-C., Lee A.Y.-L. (2021). Unlocking the Mystery of the Therapeutic Effects of Chinese Medicine on Cancer. Front. Pharmacol..

[182] Lan H.-Y., An P., Liu Q.-P., Chen Y.-Y., Yu Y.-Y., Luan X., Tang J.-Y., Zhang H. (2021). *Aidi injection* induces apoptosis of hepatocellular carcinoma cells through the mitochondrial pathway. J. Ethnopharmacol..

[183] Shi Q., Diao Y., Jin F., Ding Z. (2018). Anti-metastatic effects of Aidi on human esophageal squamous cell carcinoma by inhibiting epithelial-mesenchymal transition and angiogenesis. Mol. Med. Rep..

[184] Yang M., Shen C., Zhu S.-j., Zhang Y., Jiang H.-l., Bao Y.-d., Yang G.-y., Liu J.-p. (2022). Chin. patent medicine *Aidi injection* for cancer care: An overview of systematic reviews and meta-analyses. J. Ethnopharmacol..

[185] Zhao Z., Fan H., Higgins T., Qi J., Haines D., Trivett A., Oppenheim J.J., Wei H., Li J., Lin H. (2014). Fufang *Kushen injection* inhibits sarcoma growth and tumor-induced hyperalgesia via TRPV1 signaling pathways. Cancer Lett..

[186] Zhu A., Wang F., Fan Q., He W., Wang L., Zhao P. (2011). Apoptosis and growth arrest of human esophageal squamous cell carcinoma cell EC9706 induced by *Fufangkushen injection*. Zhonghua Yi Xue Za Zhi.

[187] Lu Y., Li C.-S., Dong Q. (2008). Chin. herb related molecules of cancer-cell-apoptosis: A minireview of progress between *Kanglaite injection* and related genes. J. Exp. Clin. Cancer Res..

[188] Liu Y., Zhang W., Wang X.-J., Liu S. (2014). Antitumor effect of Kanglaite® injection in human pancreatic cancer xenografts. BMC Complement. Altern. Med..

[189] Yang Y., Sun M., Yao W., Wang F., Li X., Wang W., Li J., Gao Z., Qiu L., You R. (2020). Compound *Kushen injection* relieves tumor-associated macrophage-mediated immunosuppression through TNFR1 and sensitizes hepatocellular carcinoma to sorafenib. J. Immunother. Cancer.

[190] Qi-Yue Y., Ting Z., Ya-Nan H., Sheng-Jie H., Xuan D., Li H., Chun-Guang X. (2020). From natural dye to herbal medicine: A systematic review of chemical constituents, pharmacological effects and clinical applications of indigo naturalis. Chin. Med..

[191] Yao C.-J., Chow J.-M., Yang C.-M., Kuo H.-C., Chang C.-L., Lee H.-L., Lai I., Chuang S.-E., Lai G.-M. (2016). Chinese herbal mixture, Tien-Hsien liquid, induces G2/M cycle arrest and radiosensitivity in MCF-7 human breast cancer cells through mechanisms involving DNMT1 and Rad51 downregulation. Evid. Based Complement. Altern. Med..

[192] Yao C.-J., Yang C.-M., Chuang S.-E., Yan J.-L., Liu C.-Y., Chen S.-W., Yan K.-H., Lai T.-Y., Lai G.-M. (2011). Targeting PML-RARα and oncogenic signaling pathways by Chin. herbal mixture Tien-Hsien liquid in acute promyelocytic leukemia NB4 cells. Evid. Based Complement. Altern. Med..

[193] Yang P.-M., Du J.-L., Wang G.N.-K., Chia J.-S., Hsu W.-B., Pu P.-C., Sun A., Chiang C.-P., Wang W.-B. (2017). The Chin. Herbal Mixture Tien-Hsien Liquid Augments the Anticancer Immunity in Tumor Cell–Vaccinated Mice. Integr. Cancer Ther..

[194] Yin T., Yang G., Ma Y., Xu B., Hu M., You M., Gao S. (2015). Developing an activity and absorption-based quality control platform for Chin. Tradit. medicine: Application to Zeng-Sheng-Ping (Antitumor B). J. Ethnopharmacol..

[195] Wang Y., Yao R., Gao S., Wen W., Du Y., Szabo E., Hu M., Lubet R.A., You M. (2013). Chemopreventive effect of a mixture of Chin. Herbs (antitumor B) on chemically induced oral carcinogenesis. Mol. Carcinogen..

[196] Lim K.J., Rajan K., Eberhart C.G. (2012). Effects of Zeng Sheng Ping/ACAPHA on malignant brain tumor growth and notch signaling. Anticancer Res..

[197] Kuruppu A.I., Paranagama P., Goonasekara C.L. (2019). Medicinal plants commonly used against cancer in Tradit. medicine formulae in Sri Lanka. Saudi Pharm. J..

[198] Sujatha V. (2020). The Universal and the Global: Contextualising European Ayurvedic Practices. Soc. Cult. South Asia.

[199] Rosenberg M. (2012). The European academy of Ayurveda: 20 years of Ayurvedic education in Germany. Anc. Sci. Life.

[200] Bhandari N. (2015). Is ayurveda the key to universal healthcare in India?. BMJ.

[201] Kumar S., Jawaid T., Dubey S.D. (2011). Ther. plants of Ayurveda; a review on anticancer. Pharmacogn. J..

[202] Palliyaguru D.L., Singh S.V., Kensler T.W. (2016). *Withania somnifera*: From Prev. to treatment of cancer. Mol. Nutr. Food Res..

[203] Singh N., Bhalla M., de Jager P., Gilca M. (2011). An overview on ashwagandha: A Rasayana (rejuvenator) of Ayurveda. Afr. J. Tradit. Complement. Altern. Med..

[204] Widodo N., Kaur K., Shrestha B.G., Takagi Y., Ishii T., Wadhwa R., Kaul S.C. (2007). Selective killing of cancer cells by leaf extract of Ashwagandha: Identification of a tumor-inhibitory factor and the first molecular insights to its effect. Clin. Cancer Res..

[205] Wadhwa R., Singh R., Gao R., Shah N., Widodo N., Nakamoto T., Ishida Y., Terao K., Kaul S.C. (2013). Water extract of Ashwagandha leaves has anticancer activity: Identification of an active component and its mechanism of action. PLoS ONE.

[206] Mehta V., Chander H., Munshi A. (2021). Mechanisms of anti-tumor activity of *Withania somnifera* (Ashwagandha). Nutr. Cancer.

[207] Kataria H., Kumar S., Chaudhary H., Kaur G. (2016). *Withania somnifera* suppresses tumor growth of intracranial allograft of glioma cells. Mol. Neurobiol..

[208] Moselhy J., Suman S., Alghamdi M., Chandarasekharan B., Das T.P., Houda A., Ankem M., Damodaran C. (2017). Withaferin A inhibits prostate carcinogenesis in a PTEN-deficient mouse model of prostate cancer. Neoplasia.

[209] Lee I.-C., Choi B.Y. (2016). Withaferin-A—A natural anticancer agent with pleitropic mechanisms of action. Int. J. Mol. Sci..

[210] Sultana T., Okla M.K., Ahmed M., Akhtar N., Al-Hashimi A., Abdelgawad H., Haq I.-U. (2021). Withaferin A: From Ancient Remedy to Potential Drug Candidate. Molecules.

[211] Kyakulaga A.H., Aqil F., Munagala R., Gupta R.C. (2018). Withaferin a inhibits epithelial to mesenchymal transition in non-small cell lung cancer cells. Sci. Rep..

[212] Hahm E.-R., Lee J., Kim S.-H., Sehrawat A., Arlotti J.A., Shiva S.S., Bhargava R., Singh S.V. (2013). Metabolic alterations in mammary cancer Prev. by withaferin A in a clinically relevant mouse model. J. Nat. Cancer Inst..

[213] Kakar S.S., Parte S., Kelsey Carter I.G.J., Worth C., Rameshwar P., Ratajczak M.Z. (2017). Withaferin A (WFA) inhibits tumor growth and metastasis by targeting ovarian cancer stem cells. Oncotarget.

[214] Kim S.-H., Singh S.V. (2014). Mammary cancer chemoPrev. by withaferin A is accompanied by in vivo suppression of self-renewal of cancer stem cells. Cancer Prev. Res..

[215] Nelson K.M., Dahlin J.L., Bisson J., Graham J., Pauli G.F., Walters M.A. (2017). The essential medicinal chemistry of curcumin: Miniperspective. J. Med. Chem..

[216] Dosoky N.S., Setzer W.N. (2018). Chemical composition and biological activities of essential oils of Curcuma species. Nutrients.

[217] Sandur S.K., Pandey M.K., Sung B., Ahn K.S., Murakami A., Sethi G., Limtrakul P., Badmaev V., Aggarwal B.B. (2007). Curcumin, demethoxycurcumin, bisdemethoxycurcumin, tetrahydrocurcumin and turmerones differentially regulate anti-inflammatory and anti-proliferative responses through a ROS-independent mechanism. Carcinogenesis.

[218] Giordano A., Tommonaro G. (2019). Curcumin and cancer. Nutrients.

[219] Sultana S., Munir N., Mahmood Z., Riaz M., Akram M., Rebezov M., Kuderinova N., Moldabayeva Z., Shariati M.A., Rauf A. (2021). Molecular targets for the Manag. of cancer using *Curcuma longa* Linn. phytoconstituents: A Review. Biomed. Pharmacother..

[220] Lev-Ari S., Starr A., Vexler A., Karaush V., Loew V., Greif J., Fenig E., Aderka D., Ben-Yosef R. (2006). Inhibition of pancreatic and lung adenocarcinoma cell survival by curcumin is associated with increased apoptosis, down-regulation of COX-2 and EGFR and inhibition of Erk1/2 activity. Anticancer Res..

[221] Zhao G., Han X., Zheng S., Li Z., Sha Y., Ni J., Sun Z., Qiao S., Song Z. (2016). Curcumin induces autophagy, inhibits proliferation and invasion by downregulating AKT/mTOR signaling pathway in human melanoma cells. Oncol. Rep..

[222] Li W., Zhou Y., Yang J., Li H., Zhang H., Zheng P. (2017). Curcumin induces apoptotic cell death and protective autophagy in human gastric cancer cells. Oncol. Rep..

[223] Zhuang W., Long L., Zheng B., Ji W., Yang N., Zhang Q., Liang Z. (2012). Curcumin promotes differentiation of glioma-initiating cells by inducing autophagy. Cancer Sci..

[224] Almanaa T.N., Geusz M.E., Jamasbi R.J. (2012). Effects of curcumin on stem-like cells in human esophageal squamous carcinoma cell lines. BMC Complement. Altern. Med..

[225] Wang J., Wang C., Bu G. (2018). Curcumin inhibits the growth of liver cancer stem cells through the phosphatidylinositol 3-kinase/protein kinase B/mammalian target of rapamycin signaling pathway. Exp. Ther. Med..

[226] Yallapu M.M., Maher D.M., Sundram V., Bell M.C., Jaggi M., Chauhan S.C. (2010). Curcumin induces chemo/radio-sensitization in ovarian cancer cells and curcumin nanoparticles inhibit ovarian cancer cell growth. J. Ovar. Res..

[227] Zhang P., Lai Z.-L., Chen H.-F., Zhang M., Wang A., Jia T., Sun W.-Q., Zhu X.-M., Chen X.-F., Zhao Z. (2017). Curcumin synergizes with 5-fluorouracil by impairing AMPK/ULK1-dependent autophagy, AKT activity and enhancing apoptosis in colon cancer cells with tumor growth inhibition in xenograft mice. J. Exp. Clin. Cancer Res..

[228] Kong W.-Y., Ngai S.C., Goh B.-H., Lee L.-H., Htar T.-T., Chuah L.-H. (2021). Is curcumin the answer to future chemotherapy cocktail?. Molecules.

[229] Farghadani R., Naidu R. (2022). Curcumin as an Enhancer of Ther. Efficiency of Chemotherapy Drugs in Breast Cancer. Int. J. Mol. Sci..

[230] Hsu F.-T., Liu Y.-C., Liu T.-T., Hwang J.-J. (2015). Curcumin sensitizes hepatocellular carcinoma cells to radiation via suppression of radiation-induced NF-κB activity. Biomed Res. Int..

[231] Zoi V., Galani V., Tsekeris P., Kyritsis A.P., Alexiou G.A. (2022). Radiosensitization and Radioprotection by Curcumin in Glioblastoma and Other Cancers. Biomedicines.

[232] Prasad S., Tyagi A.K. (2015). Ginger and its constituents: Role in Prev. and treatment of gastrointestinal cancer. Gastroenterol. Res. Pract..

[233] Mao Q.-Q., Xu X.-Y., Cao S.-Y., Gan R.-Y., Corke H., Beta T., Li H.-B. (2019). Bioactive compounds and bioactivities of ginger (*Zingiber officinale* Roscoe). Foods.

[234] de Lima R.M.T., Dos Reis A.C., de Menezes A.A.P.M., Santos J.V.d.O., Filho J.W.G.d.O., Ferreira J.R.d.O., de Alencar M.V.O.B., da Mata A.M.O.F., Khan I.N., Islam A. (2018). Protective and therapeutic potential of ginger (*Zingiber officinale*) extract and [6]-gingerol in cancer: A comprehensive review. Phytother. Res..

[235] Habib S.H.M., Makpol S., Hamid N.A.A., Das S., Ngah W.Z.W., Yusof Y.A.M. (2008). Ginger extract (*Zingiber officinale*) has anti-cancer and anti-inflammatory effects on ethionine-induced hepatoma rats. Clinics.

[236] Kim S.M., Kim C., Bae H., Lee J.H., Baek S.H., Nam D., Chung W.S., Shim B.S., Lee S.G., Kim S.H. (2015). 6-Shogaol exerts anti-proliferative and pro-apoptotic effects through the modulation of STAT3 and MAPKs signaling pathways. Mol. Carcinog..

[237] Saha A., Blando J., Silver E., Beltran L., Sessler J., DiGiovanni J. (2014). 6-Shogaol from dried ginger inhibits growth of prostate cancer cells both in vitro and in vivo through inhibition of STAT3 and NF-κB signaling. Cancer Prev. Res..

[238] Kim Y.-J., Jeon Y., Kim T., Lim W.-C., Ham J., Park Y.N., Kim T.-J., Ko H. (2017). Combined treatment with zingerone and its novel derivative synergistically inhibits TGF-β1 induced epithelial-mesenchymal transition, migration and invasion of human hepatocellular carcinoma cells. Bioorg. Med. Chem. Lett..

[239] Ray A., Vasudevan S., Sengupta S. (2015). 6-Shogaol inhibits breast cancer cells and stem cell-like spheroids by modulation of Notch signaling pathway and induction of autophagic cell death. PLoS ONE.

[240] El-Ashmawy N.E., Khedr N.F., El-Bahrawy H.A., Abo Mansour H.E. (2018). Ginger extract adjuvant to doxorubicin in mammary carcinoma: Study of some molecular mechanisms. Eur. J. Nutr..

[241] Rahimi Babasheikhali S., Rahgozar S., Mohammadi M. (2019). Ginger extract has anti-leukemia and anti-drug resistant effects on malignant cells. J. Cancer Res. Clin. Oncol..

[242] Liu C.-M., Kao C.-L., Tseng Y.-T., Lo Y.-C., Chen C.-Y. (2017). Ginger phytochemicals inhibit cell growth and modulate drug resistance factors in docetaxel resistant prostate cancer cell. Molecules.

[243] Almatroudi A., Alsahli M.A., Alrumaihi F., Allemailem K.S., Rahmani A.H. (2019). Ginger: A novel strategy to battle cancer through modulating cell signalling pathways: A review. Curr. Pharm. Biotechnol..

[244] Zadorozhna M., Mangieri D. (2021). Mechanisms of chemopreventive and Ther. proprieties of ginger extracts in cancer. Int. J. Mol. Sci..

[245] Iram F., Khan S.A., Husain A. (2017). Phytochemistry and potential Ther. actions of Boswellic acids: A mini-review. Asian Pac. J. Trop. Biomed..

[246] Roy N.K., Parama D., Banik K., Bordoloi D., Devi A.K., Thakur K.K., Padmavathi G., Shakibaei M., Fan L., Sethi G. (2019). An update on pharmacological potential of boswellic acids against chronic diseases. Int. J. Mol. Sci..

[247] Gupta M., Rout P., Misra L., Gupta P., Singh N., Darokar M., Saikia D., Singh S., Bhakuni R. (2017). Chemical composition and bioactivity of *Boswellia serrata* Roxb. essential oil in relation to geographical variation. Plant Biosyst. Int. J. All Aspects Plant Biol..

[248] Ayub M.A., Hanif M.A., Blanchfield J., Zubair M., Abid M.A., Saleh M.T. (2022). Chemical composition and antimicrobial activity of *Boswellia serrata* oleo-gum-resin essential oil extracted by superheated steam. Nat. Prod. Res..

[249] Khajehdehi M., Khalaj-Kondori M., Baradaran B. (2022). Molecular evidences on anti-inflammatory, anticancer, and memory-boosting effects of frankincense. Phytother. Res..

[250] Ahmed H.H., Abd-Rabou A.A., Hassan A.Z., Kotob S.E. (2015). Phytochemical analysis and anti-cancer investigation of *Boswellia serrata* bioactive constituents in vitro. Asian Pac. J. Cancer Prev..

[251] Hakkim F.L., Bakshi H.A., Khan S., Nasef M., Farzand R., Sam S., Rashan L., Al-Baloshi M.S., Hasson S.S.A.A., Al Jabri A. (2019). Frankincense essential oil suppresses melanoma cancer through down regulation of Bcl-2/Bax cascade signaling and ameliorates heptotoxicity via phase I and II drug metabolizing enzymes. Oncotarget.

[252] Takada Y., Ichikawa H., Badmaev V., Aggarwal B.B. (2006). Acetyl-11-keto-β-boswellic acid potentiates apoptosis, inhibits invasion, and abolishes osteoclastogenesis by suppressing NF-κB and NF-κB-regulated gene expression. J. Immunol..

[253] Kunnumakkara A.B., Nair A.S., Sung B., Pandey M.K., Aggarwal B.B. (2009). Boswellic acid blocks STAT3 Signaling, proliferation, and survival of multiple myeloma via the protein tyrosine phosphatase SHP-1. Mol. Cancer Res. MCR.

[254] Shen Y., Takahashi M., Byun H.-M., Link A., Sharma N., Balaguer F., Leung H.-C., Boland C.R., Goel A. (2012). Boswellic acid induces epigenetic alterations by modulating DNA methylation in colorectal cancer cells. Cancer Biol. Ther..

[255] Mazzio E.A., Lewis C.A., Soliman K.F. (2017). Transcriptomic Profiling of MDA-MB-231 cells exposed to *Boswellia Serrata* and 3-O-Acetyl-B-Boswellic Acid; ER/UPR mediated programmed cell death. Cancer Genom. Proteom..

[256] Hussain H., Ali I., Wang D., Hakkim F.L., Westermann B., Rashan L., Ahmed I., Green I.R. (2021). Boswellic acids: Privileged structures to develop lead compounds for anticancer drug discovery. Expert Opin. Drug Discov..

[257] Hussain H., Al-Harrasi A., Csuk R., Shamraiz U., Green I.R., Ahmed I., Khan I.A., Ali Z. (2017). Therapeutic potential of boswellic acids: A patent review (1990–2015). Expert Opin. Ther. Pat..

[258] Takeda S., Noguchi M., Matsuo K., Yamaguchi Y., Kudo T., Nishimura H., Okamoto Y., Amamoto T., Shindo M., Omiecinski C.J. (2013). (−)-Xanthatin up-regulation of the GADD45γ tumor suppressor gene in MDA-MB-231 breast cancer cells: Role of topoisomerase IIα inhibition and reactive oxygen species. Toxicology.

[259] Yu Y., Yu J., Pei C.G., Li Y.Y., Tu P., Gao G.P., Shao Y. (2015). Xanthatin, a novel potent inhibitor of VEGFR2 signaling, inhibits angiogenesis and tumor growth in breast cancer cells. Int. J. Clin. Exp. Pathol..

[260] Zhang L., Ruan J., Yan L., Li W., Wu Y., Tao L., Zhang F., Zheng S., Wang A., Lu Y. (2012). Xanthatin induces cell cycle arrest at G2/M checkpoint and apoptosis via disrupting NF-κB pathway in A549 non-small-cell lung cancer cells. Molecules.

[261] Tao L., Fan F., Liu Y., Li W., Zhang L., Ruan J., Shen C., Sheng X., Zhu Z., Wang A. (2013). Concerted suppression of STAT3 and GSK3β is involved in growth inhibition of non-small cell lung cancer by xanthatin. PLoS ONE.

[262] Tao L., Sheng X., Zhang L., Li W., Wei Z., Zhu P., Zhang F., Wang A., Woodgett J.R., Lu Y. (2016). Xanthatin anti-tumor cytotoxicity is mediated via glycogen synthase kinase-3β and β-catenin. Biochem. Pharmacol..

[263] Yang J., Li Y., Zong C., Zhang Q., Ge S., Ma L., Fan J., Zhang J., Jia R. (2021). Xanthatin Selectively Targets Retinoblastoma by Inhibiting the PLK1-Mediated Cell Cycle. Investig. Ophthalmol. Visual Sci..

[264] Geng Y.-d., Zhang L., Wang G.-Y., Feng X.-J., Chen Z.-L., Jiang L., Shen A.-Z. (2020). Xanthatin mediates G2/M cell cycle arrest, autophagy and apoptosis via ROS/XIAP signaling in human colon cancer cells. Nat. Prod. Res..

[265] Li L., Liu P., Xie Y., Liu Y., Chen Z., Geng Y., Zhang L. (2022). Xanthatin inhibits human colon cancer cells progression via mTOR signaling mediated energy metabolism alteration. Drug Dev. Res..

[266] Ma Y.-Y., Di Z.-M., Cao Q., Xu W.-S., Bi S.-X., Yu J.-S., Shen Y.-J., Yu Y.-Q., Shen Y.-X., Feng L.-J. (2020). Xanthatin induces glioma cell apoptosis and inhibits tumor growth via activating endoplasmic reticulum stress-dependent CHOP pathway. Acta Pharmacol. Sin..

[267] Hashim Y., Latimer C., Ternan N., Abbas P. (2016). Studies of Malaysian plants in prevention and treatment of colorectal cancer. Colorectal Cancer.

[268] Mazumder K., Biswas B., Raja I.M., Fukase K. (2020). A review of cytotoxic plants of the Indian subcontinent and a broad-spectrum analysis of their bioactive compounds. Molecules.

[269] Meiyanto E., Larasati Y.A. (2019). The chemopreventive activity of Indonesia medicinal plants targeting on hallmarks of cancer. Adv. Pharm. Bull..

[270] Sithisarn P., Rojsanga P. (2017). Anticancer Effects of Some Medicinal Thai Plants. Natural Products and Cancer Drug Discovery.

[271] Nguyen N.H., Ta Q.T.H., Pham Q.T., Luong T.N.H., Phung V.T., Duong T.-H., Vo V.G. (2020). Anticancer activity of novel plant extracts and compounds from Adenosma bracteosum (Bonati) in human lung and liver cancer cells. Molecules.

[272] Shuvalov O., Fedorova O., Tananykina E., Gnennaya Y., Daks A., Petukhov A., Barlev N. (2020). An arthropod hormone, ecdysterone, inhibits the growth of breast cancer cells via different mechanisms. Front. Pharmacol..

[273] Zirak N., Shafiee M., Soltani G., Mirzaei M., Sahebkar A. (2019). *Hypericum perforatum* in the treatment of psychiatric and neurodegenerative disorders: Current evidence and potential mechanisms of action. J. Cell. Physiol..

[274] Menegazzi M., Masiello P., Novelli M. (2020). Anti-tumor activity of *Hypericum perforatum* L. and hyperforin through modulation of inflammatory signaling, ROS generation and proton dynamics. Antioxidants.

[275] Napoli E., Siracusa L., Ruberto G., Carrubba A., Lazzara S., Speciale A., Cimino F., Saija A., Cristani M. (2018). Phytochemical profiles, phototoxic and antioxidant properties of eleven Hypericum species–A comparative study. Phytochem..

[276] Alper M., Güneş H. (2019). The anticancer and anti-inflammatory effects of *Centaurea solstitialis* extract on human cancer cell lines. Turk. J. Pharm. Sci..

[277] Koeberle A., Rossi A., Bauer J., Dehm F., Verotta L., Northoff H., Sautebin L., Werz O. (2011). Hyperforin, an anti-inflammatory constituent from St. John’s wort, inhibits microsomal prostaglandin E2 synthase-1 and suppresses prostaglandin E2 formation in vivo. Front. Pharmacol..

[278] Benedí J., Arroyo R., Romero C., Martín-Aragón S., Villar A.M. (2004). Antioxidant properties and protective effects of a standardized extract of *Hypericum perforatum* on hydrogen peroxide-induced oxidative damage in PC12 cells. Life Sci..

[279] Imreova P., Feruszova J., Kyzek S., Bodnarova K., Zduriencikova M., Kozics K., Mucaji P., Galova E., Sevcovicova A., Miadokova E. (2017). Hyperforin exhibits antigenotoxic activity on human and bacterial cells. Molecules.

[280] Hsu F.T., Chen W.T., Wu C.T., Chung J.G. (2020). Hyperforin induces apoptosis through extrinsic/intrinsic pathways and inhibits EGFR/ERK/NF-κB-mediated anti-apoptotic potential in glioblastoma. Environ. Toxicol..

[281] Merhi F., Tang R., Piedfer M., Mathieu J., Bombarda I., Zaher M., Kolb J.-P., Billard C., Bauvois B. (2011). Hyperforin inhibits Akt1 kinase activity and promotes caspase-mediated apoptosis involving Bad and Noxa activation in human myeloid tumor cells. PLoS ONE.

[282] Quiney C., Billard C., Faussat A., Salanoubat C., Ensaf A., Nait-Si Y., Fourneron J., Kolb J. (2006). Pro-apoptotic properties of hyperforin in leukemic cells from patients with B-cell chronic lymphocytic leukemia. Leukemia.

[283] Lorusso G., Vannini N., Sogno I., Generoso L., Garbisa S., Noonan D.M., Albini A. (2009). Mechanisms of Hyperforin as an anti-angiogenic angioPrev. agent. Eur. J. Cancer.

[284] Donà M., Dell’Aica I., Pezzato E., Sartor L., Calabrese F., Della Barbera M., Donella-Deana A., Appendino G., Borsarini A., Caniato R. (2004). Hyperforin inhibits cancer invasion and metastasis. Cancer Res..

[285] Sell T.S., Belkacemi T., Flockerzi V., Beck A. (2014). Protonophore properties of hyperforin are essential for its pharmacological activity. Sci. Rep..

[286] Scotti F., Löbel K., Booker A., Heinrich M. (2019). St. John’s Wort (*Hypericum perforatum*) products–How variable is the primary material?. Front. Plant Sci..

[287] Fulda S. (2008). Betulinic acid for cancer treatment and prevention. Int. J. Mol. Sci..

[288] Zhang X., Hu J., Chen Y. (2016). Betulinic acid and the pharmacological effects of tumor suppression. Mol. Med. Rep..

[289] Tan Y., Yu R., Pezzuto J.M. (2003). Betulinic acid-induced programmed cell death in human melanoma cells involves mitogen-activated protein kinase activation. Clin. Cancer Res..

[290] Guo Y., Zhu H., Weng M., Wang C., Sun L. (2020). Chemopreventive effect of Betulinic acid via mTOR-Caspases/Bcl2/Bax apoptotic signaling in pancreatic cancer. BMC Complement. Med. Ther..

[291] Wang S., Wang K., Zhang C., Zhang W., Xu Q., Wang Y., Zhang Y., Li Y., Zhang Y., Zhu H. (2017). Overaccumulation of p53-mediated autophagy protects against betulinic acid-induced apoptotic cell death in colorectal cancer cells. Cell Death Dis..

[292] Potze L., Mullauer F., Colak S., Kessler J., Medema J. (2014). Betulinic acid-induced mitochondria-dependent cell death is counterbalanced by an autophagic salvage response. Cell Death Dis..

[293] Zheng Y., Liu P., Wang N., Wang S., Yang B., Li M., Chen J., Situ H., Xie M., Lin Y. (2019). Betulinic acid suppresses breast cancer metastasis by targeting GRP78-mediated glycolysis and ER stress apoptotic pathway. Oxid. Med. Cell. Longev..

[294] Saeed M.E., Mahmoud N., Sugimoto Y., Efferth T., Abdel-Aziz H. (2018). Betulinic acid exerts cytotoxic activity against multidrug-resistant tumor cells via targeting autocrine motility factor receptor (AMFR). Front. Pharmacol..

[295] Nedopekina D.A., Gubaidullin R.R., Odinokov V.N., Maximchik P.V., Zhivotovsky B., Bel’skii Y.P., Khazanov V.A., Manuylova A.V., Gogvadze V., Spivak A.Y. (2017). Mitochondria-targeted betulinic and ursolic acid derivatives: Synthesis and anticancer activity. MedChemComm.

[296] Sharma V., Katiyar A., Agrawal R. (2018). *Glycyrrhiza glabra*: Chemistry and pharmacological activity. Sweeteners.

[297] Wang K.-L., Yu Y.-C., Hsia S.-M. (2021). Perspectives on the Role of Isoliquiritigenin in Cancer. Cancers.

[298] Tian T., Sun J., Wang J., Liu Y., Liu H. (2018). Isoliquiritigenin inhibits cell proliferation and migration through the PI3K/AKT signaling pathway in A549 lung cancer cells. Oncol. Lett..

[299] Chen C., Shenoy A.K., Padia R., Fang D., Jing Q., Yang P., Su S.-B., Huang S. (2018). Suppression of lung cancer progression by isoliquiritigenin through its metabolite 2, 4, 2’, 4’-Tetrahydroxychalcone. J. Exp. Clin. Cancer Res..

[300] Bolós V., Gasent J.M., López-Tarruella S., Grande E. (2010). The dual kinase complex FAK-Src as a promising therapeutic target in cancer. Onco Targets Ther..

[301] Nag S., Qin J., Srivenugopal K.S., Wang M., Zhang R. (2013). The MDM2-p53 pathway revisited. J. Biomed. Res..

[302] Bohlman S., Manfredi J.J. (2014). p53-independent effects of Mdm2. Mutant p53 and MDM2 in Cancer. Subcell Biochem..

[303] Shuvalov O., Kizenko A., Shakirova A., Fedorova O., Petukhov A., Aksenov N., Vasileva E., Daks A., Barlev N. (2018). Nutlin sensitizes lung carcinoma cells to interferon-alpha treatment in MDM2-dependent but p53-independent manner. Biochem. Biophys. Res. Commun..

[304] Xiang S., Chen H., Luo X., An B., Wu W., Cao S., Ruan S., Wang Z., Weng L., Zhu H. (2018). Isoliquiritigenin suppresses human melanoma growth by targeting miR-301b/LRIG1 signaling. J. Exp. Clin. Cancer Res..

[305] Kroll D.J., Shaw H.S., Oberlies N.H. (2007). Milk thistle nomenclature: Why it matters in cancer research and pharmacokinetic studies. Integr. Cancer Ther..

[306] Karimi G., Vahabzadeh M., Lari P., Rashedinia M., Moshiri M. (2011). “Silymarin”, a promising pharmacological agent for treatment of diseases. Iran. J. Basic Med. Sci..

[307] Won D.-H., Kim L.-H., Jang B., Yang I.-H., Kwon H.-J., Jin B., Oh S.H., Kang J.-H., Hong S.-D., Shin J.-A. (2018). In vitro and in vivo anti-cancer activity of silymarin on oral cancer. Tumor Biol..

[308] Eo H.J., Park G.H., Jeong J.B. (2016). Inhibition of Wnt signaling by silymarin in human colorectal cancer cells. Biomol. Ther..

[309] Delmas D., Xiao J., Vejux A., Aires V. (2020). Silymarin and Cancer: A Dual Strategy in Both in Chemoprevention and Chemosensitivity. Molecules.

[310] Ramasamy K., Agarwal R. (2008). Multitargeted therapy of cancer by silymarin. Cancer Lett..

[311] Kim S.H., Choo G.S., Yoo E.S., Woo J.S., Han S.H., Lee J.H., Jung J.Y. (2019). Silymarin induces inhibition of growth and apoptosis through modulation of the MAPK signaling pathway in AGS human gastric cancer cells. Oncol. Rep..

[312] Mao J., Yang H., Cui T., Pan P., Kabir N., Chen D., Ma J., Chen X., Chen Y., Yang Y. (2018). Combined treatment with sorafenib and silibinin synergistically targets both HCC cells and cancer stem cells by enhanced inhibition of the phosphorylation of STAT3/ERK/AKT. Eur. J. Pharmacol..

[313] Soleimani V., Delghandi P.S., Moallem S.A., Karimi G. (2019). Safety and toxicity of silymarin, the major constituent of milk thistle extract: An updated review. Phytother. Res..

[314] Shang A., Cao S.-Y., Xu X.-Y., Gan R.-Y., Tang G.-Y., Corke H., Mavumengwana V., Li H.-B. (2019). Bioactive compounds and biological functions of garlic (*Allium sativum* L.). Foods.

[315] Taleb Agha M., Baharetha H.M., Al-Mansoub M.A., Tabana Y.M., Kaz Abdul Aziz N.H., Yam M.F., Abdul Majid A.M.S. (2020). Proapoptotic and antiangiogenic activities of *Arctium lappa* L. on breast cancer cell lines. Scientifica.

[316] Li X., Lin Y.-Y., Tan J.-Y., Liu K.-L., Shen X.-L., Hu Y.-J., Yang R.-Y. (2021). Lappaol F, an anticancer agent, inhibits YAP via transcriptional and post-translational regulation. Pharm. Biol..

[317] He Y., Fan Q., Cai T., Huang W., Xie X., Wen Y., Shi Z. (2018). Molecular mechanisms of the action of Arctigenin in cancer. Biomed. Pharmacother..

[318] Erenler R., Sen O., Sahin Yaglioglu A., Demirtas I. (2016). Bioactivity-guided isolation of antiproliferative sesquiterpene lactones from *Centaurea solstitialis* L. ssp. solstitialis. Comb. Chem. High Throughput Screen..

[319] Simsek E., Imir N., Aydemir E.A., Gokturk R.S., Yesilada E., Fiskin K. (2017). Caspase-mediated apoptotic effects of *Ebenus boissieri* barbey extracts on human cervical cancer cell line hela. Pharmacogn. Mag..

[320] Aydemir E.A., Simsek E., Imir N., Göktürk R.S., Yesilada E., Fiskin K. (2015). Cytotoxic and apoptotic effects of *Ebenus boissieri* Barbey on human lung cancer cell line A549. Pharmacogn. Mag..

[321] Imir N., Aydemir E., Şimşek E., Göktürk R., Yesilada E., Fişkin K. (2016). Cytotoxic and immunomodulatory effects of *Ebenus boissieri* Barbey on breast cancer cells. Genet. Mol. Res..

[322] Allegra A., Tonacci A., Pioggia G., Musolino C., Gangemi S. (2020). Anticancer activity of *Rosmarinus officinalis* L.: Mechanisms of Action and Therapeutic Potentials. Nutrients.

[323] Kowalczyk T., Sitarek P., Skała E., Toma M., Wielanek M., Pytel D., Wieczfińska J., Szemraj J., Śliwiński T. (2019). Induction of apoptosis by in vitro and in vivo plant extracts derived from *Menyanthes trifoliata* L. in human cancer cells. Cytotechnology.

[324] Vervandier-Fasseur D., Latruffe N. (2019). The potential use of resveratrol for cancer prevention. Molecules.

[325] Aja I., Ruiz-Larrea M.B., Courtois A., Krisa S., Richard T., Ruiz-Sanz J.-I. (2020). Screening of natural stilbene oligomers from *Vitis vinifera* for anticancer activity on human hepatocellular carcinoma cells. Antioxidants.

[326] Marvibaigi M., Supriyanto E., Amini N., Abdul Majid F.A., Jaganathan S.K. (2014). Preclinical and clinical effects of mistletoe against breast cancer. Biomed Res. Int..

[327] de Oliveira Melo M.N., Oliveira A.P., Wiecikowski A.F., Carvalho R.S., de Lima Castro J., de Oliveira F.A.G., Pereira H.M.G., da Veiga V.F., Capella M.M.A., Rocha L. (2018). Phenolic compounds from *Viscum album* tinctures enhanced antitumor activity in melanoma murine cancer cells. Saudi Pharm. J..

[328] Chen T., Li B., Qiu Y., Qiu Z., Qu P. (2018). Funct. mechanism of Ginsenosides on tumor growth and metastasis. Saudi J. Biol. Sci..

[329] Jia L., Zhao Y. (2009). Current evaluation of the millennium phytomedicine-ginseng (I): Etymology, pharmacognosy, phytochemistry, market and regulations. Curr. Med. Chem..

[330] Hong H., Baatar D., Hwang S.G. (2021). Anticancer activities of ginsenosides, the main active components of ginseng. Evid. Based Complement. Altern. Med..

[331] Gao J.-L., Lv G.-Y., He B.-C., Zhang B.-Q., Zhang H., Wang N., Wang C.-Z., Du W., Yuan C.-S., He T.-C. (2013). Ginseng saponin metabolite 20 (S)-protopanaxadiol inhibits tumor growth by targeting multiple cancer signaling pathways. Oncol. Rep..

[332] Zhang X., Han L., Li P., Zhang S., Zhang M., Li X., Chu J., Wang L., Tu P., Zhang Y. (2021). Region-Specific Biomarkers and Their Mechanisms in the Treatment of Lung Adenocarcinoma: A Study of *Panax quinquefolius* from Wendeng, China. Molecules.

[333] Ham S.W., Kim J.-K., Jeon H.-Y., Kim E.-J., Jin X., Eun K., Park C.G., Lee S.Y., Seo S., Kim J.Y. (2019). Korean Red ginseng extract inhibits glioblastoma propagation by blocking the Wnt signaling pathway. J. Ethnopharmacol..

[334] Guo J.-Q., Zheng Q.-H., Chen H., Chen L., Xu J.-B., Chen M.-Y., Lu D., Wang Z.-H., Tong H.-F., Lin S. (2014). Ginsenoside Rg3 inhibition of vasculogenic mimicry in pancreatic cancer through downregulation of VE-cadherin/EphA2/MMP9/MMP2 expression. Int. J. Oncol..

[335] Nag S.A., Qin J., Wang W., Wang M.-H., Wang H., Zhang R. (2012). Ginsenosides as anticancer agents: In vitro and in vivo activities, structure-activity relationships, and molecular mechanisms of action. Front. Pharmacol..

[336] Lerma-Herrera M.A., Beiza-Granados L., Ochoa-Zarzosa A., López-Meza J.E., Hernández-Hernández J.D., Aviña-Verduzco J., García-Gutiérrez H.A. (2021). In vitro cytotoxic potential of extracts from *Aristolochia foetida* Kunth against MCF-7 and bMECs cell lines. Saudi J. Biol. Sci..

[337] Nam J.-S., Park S.-Y., Lee S.-O., Lee H.-J., Jang H.-L., Rhee Y.H. (2021). The growth-inhibitory effects of pawpaw (*Asimina triloba* [L.] Dunal) roots, twigs, leaves, and fruit against human gastric (AGS) and cervical (HeLa) cancer cells and their anti-inflammatory activities. Mol. Biology Rep..

[338] Wisintainer G., Scola G., Moura S., Lemos T., Pessoa C., de Moraes M., Souza L., Roesch-Ely M., Henriques J. (2015). O-naphthoquinone isolated from *Capraria biflora* L. induces selective cytotoxicity in tumor cell lines. Genet. Mol. Res..

[339] Barbosa-Jobim G.S., Costa-Lira E., Ralph A.C.L., Gregorio L., Lemos T.L., Burbano R.R., Calcagno D.Q., Smith M.A., Montenegro R.C., Vasconcellos M.C. (2020). Biflorin inhibits the proliferation of gastric cancer cells by decreasing MYC expression. Toxicol. In Vitro.

[340] Tang C., Gong L., Qiu K., Zhang Z., Wan L. (2020). Echinacoside inhibits breast cancer cells by suppressing the Wnt/β-catenin signaling pathway. Biochem. Biophys. Res. Commun..

[341] Hosami F., Manayi A., Salimi V., Khodakhah F., Nourbakhsh M., Nakstad B., Tavakoli-Yaraki M. (2021). The pro-apoptosis effects of *Echinacea purpurea* and Cannabis sativa extracts in human lung cancer cells through caspase-dependent pathway. BMC Complement. Med. Ther..

[342] Kuttikrishnan S., Siveen K.S., Prabhu K.S., Khan A.Q., Akhtar S., Mateo J.M., Merhi M., Taha R., Omri H.E., Mraiche F. (2019). Sanguinarine suppresses growth and induces apoptosis in childhood acute lymphoblastic leukemia. Leuk. Lymphoma.

[343] Tuzimski T., Petruczynik A., Plech T., Kaproń B., Makuch-Kocka A., Szultka-Młyńska M., Misiurek J., Buszewski B. (2021). Determination of Cytotoxic Activity of *Sanguinaria canadensis* Extracts against Human Melanoma Cells and Comparison of Their Cytotoxicity with Cytotoxicity of Some Anticancer Drugs. Molecules.

[344] Croaker A., King G.J., Pyne J.H., Anoopkumar-Dukie S., Liu L. (2016). *Sanguinaria canadensis*: Traditional medicine, phytochemical composition, biological activities and current uses. Int. J. Mol. Sci..

[345] Singab A.N., Youssef F.S., Ashour M.L., Wink M. (2013). The genus Eremophila (Scrophulariaceae): An ethnobotanical, biological and phytochemical review. J. Pharm. Pharmacol..

[346] Petersen M.J., Lund X.L., Semple S.J., Buirchell B., Franzyk H., Gajhede M., Kongstad K.T., Stenvang J., Staerk D. (2021). Reversal of ABCG2/BCRP-Mediated Multidrug Resistance by 5, 3′, 5′-Trihydroxy-3, 6, 7, 4′-Tetramethoxyflavone Isolated from the Australian Desert Plant *Eremophila galeata* Chinnock. Biomolecules.

[347] An G., Morris M.E. (2020). Efflux transporters in cancer resistance: Molecular and Funct. characterization of breast cancer resistance protein. Drug Efflux Pumps in Cancer Resistance Pathways: From Molecular Recognition and Characterization to Possible Inhibition Strategies in Chemotherapy.

[348] Shalom J., Cock I.E. (2018). *Terminalia ferdinandiana* Exell. fruit and leaf extracts inhibit proliferation and induce apoptosis in selected human cancer cell lines. Nutr. Cancer.

[349] Mohanty S., Cock I.E. (2012). The chemoTher. potential of *Terminalia ferdinandiana*: Phytochemistry and bioactivity. Pharmacogn. Rev..

[350] Sakulnarmrat K., Fenech M., Thomas P., Konczak I. (2013). Cytoprotective and pro-apoptotic activities of native Australian herbs polyphenolic-rich extracts. Food Chem..

[351] Chuen T.L., Vuong Q.V., Hirun S., Bowyer M.C., Predebon M.J., Goldsmith C.D., Sakoff J.A., Scarlett C.J. (2016). Antioxidant and anti-proliferative properties of Davidson’s plum (*Davidsonia pruriens* F. Muell) phenolic-enriched extracts as affected by different extraction solvents. J. Herbal Med..

[352] Jamieson N., Sirdaarta J., Cock I. (2014). The Anti-Proliferative Properties of Australian Plants with High Antioxidant Capacities Against Cancer Cell Lines. Pharmacogn. Commun..

[353] Bäcker C., Jenett-Siems K., Siems K., Wurster M., Bodtke A., Lindequist U. (2014). Cytotoxic saponins from the seeds of *Pittosporum angustifolium*. Z. Nat. C.

[354] Hawksworth D.L., Lücking R. (2017). Fungal diversity revisited: 2.2 to 3.8 million species. Microbiol. Spectr..

[355] Nowakowski P., Markiewicz-Żukowska R., Bielecka J., Mielcarek K., Grabia M., Socha K. (2021). Treasures from the forest: Evaluation of mushroom extracts as anti-cancer agents. Biomed. Pharmacother..

[356] Al-Obaidi J.R., Jambari N.N., Ahmad-Kamil E. (2021). Mycopharmaceuticals and Nutraceuticals: Promising Agents to Improve Human Well-Being and Life Quality. J. Fungi.

[357] Keller N.P. (2019). Fungal secondary metabolism: Regulation, function and drug discovery. Nat. Rev. Microbiol..

[358] Avalos J., Limón M.C. (2021). Fungal Secondary Metabolism. Encyclopedia.

[359] Szychowski K.A., Skóra B., Pomianek T., Gmiński J. (2021). *Inonotus obliquus*–from folk medicine to clinical use. J. Tradit. Complement. Med..

[360] Yukawa H., Ishikawa S., Kawanishi T., Tamesada M., Tomi H. (2012). Direct cytotoxicity of *Lentinula edodes* mycelia extract on human hepatocellular carcinoma cell line. Biol. Pharm. Bull..

[361] Israilides C., Kletsas D., Arapoglou D., Philippoussis A., Pratsinis H., Ebringerová A., Hříbalová V., Harding S. (2008). In vitro cytostatic and immunomodulatory properties of the medicinal mushroom *Lentinula edodes*. Phytomedicine.

[362] Liu W., Gu J., Qi J., Zeng X.N., Ji J., Chen Z.Z., Sun X.L. (2015). Lentinan exerts synergistic apoptotic effects with paclitaxel in A549 cells via activating ROS-TXNIP-NLRP 3 inflammasome. J. Cell. Mol. Med..

[363] Sun M., Zhao W., Xie Q., Zhan Y., Wu B. (2015). Lentinan reduces tumor progression by enhancing gemcitabine chemotherapy in urothelial bladder cancer. Surg. Oncol..

[364] Zhao L., Xiao Y., Xiao N. (2013). Effect of lentinan combined with docetaxel and cisplatin on the proliferation and apoptosis of BGC823 cells. Tumor Biol..

[365] Zhang Y., Li Q., Wang J., Cheng F., Huang X., Cheng Y., Wang K. (2016). Polysaccharide from Lentinus edodes combined with oxaliplatin possesses the synergy and attenuation effect in hepatocellular carcinoma. Cancer Lett..

[366] Wang Y., Chen J., Han Q., Luo Q., Zhang H., Wang Y. (2019). Construction of doxorubicin-conjugated lentinan nanoparticles for enhancing the cytotoxocity effects against breast cancer cells. Coll. Surf. A Physicochem. Eng. Asp..

[367] Wang J., Li W., Huang X., Liu Y., Li Q., Zheng Z., Wang K. (2017). A polysaccharide from Lentinus edodes inhibits human colon cancer cell proliferation and suppresses tumor growth in athymic nude mice. Oncotarget.

[368] Ina K., Kataoka T., Ando T. (2013). The use of lentinan for treating gastric cancer. Anticancer Agents Med. Chem..

[369] Ina H., Yoneda M., Kanda M., Kodera Y., Kabeya M., Yuasa S. (2016). Lentinan, a shiitake mushroom beta-glucan, stimulates tumor-specific adaptive immunity through PD-L1 down-regulation in gastric cancer cells. Med. Chem.

[370] Antonelli M., Donelli D., Firenzuoli F. (2020). Lentinan for Integrative Cancer Treatment: An Umbrella Review. Multidiscip. Digit. Publ. Inst. Proc..

[371] Zhang M., Zhang Y., Zhang L., Tian Q. (2019). Mushroom polysaccharide lentinan for treating different types of cancers: A review of 12 years clinical studies in China. Progress Mol. Biol. Translat. Sci..

[372] Zhao H., Wu L., Yan G., Chen Y., Zhou M., Wu Y., Li Y. (2021). Inflammation and tumor progression: Signaling pathways and targeted intervention. Signal Transduct. Target. Ther..

[373] Basu A., Ramamoorthi G., Albert G., Gallen C., Beyer A., Snyder C., Koski G., Disis M.L., Czerniecki B.J., Kodumudi K. (2021). Differentiation and regulation of TH cells: A balancing act for cancer immunotherapy. Front. Immunol..

[374] Ina K., Furuta R., Kataoka T., Kayukawa S., Yoshida T., Miwa T., Yamamura Y., Takeuchi Y. (2011). Lentinan prolonged survival in patients with gastric cancer receiving S-1-based chemotherapy. World J. Clin. Oncol..

[375] Howard R., Kanetsky P.A., Egan K.M. (2019). Exploring the prognostic value of the neutrophil-to-lymphocyte ratio in cancer. Sci. Rep..

[376] Szor D.J., Dias A.R., Pereira M.A., Ramos M.F.K.P., Zilberstein B., Cecconello I., Ribeiro-Júnior U. (2018). Prognostic role of neutrophil/lymphocyte ratio in resected gastric cancer: A systematic review and meta-analysis. Clinics.

[377] Tavakkoli M., Wilkins C.R., Mones J.V., Mauro M.J. (2019). A novel paradigm between leukocytosis, G-CSF secretion, neutrophil-to-lymphocyte ratio, myeloid-derived suppressor cells, and prognosis in non-small cell lung cancer. Front. Oncol..

[378] Matsuoka H., Seo Y., Wakasugi H., Saito T., Tomoda H. (1997). Lentinan potentiates immunity and prolongs the survival time of some patients. Anticancer Res..

[379] Wang X.-E., Wang Y.-H., Zhou Q., Peng M., Zhang J., Chen M., Ma L.-J., Xie G.-M. (2020). Immunomodulatory effect of lentinan on aberrant T subsets and cytokines profile in non-small cell lung cancer patients. Pathol. Oncol. Res..

[380] Oba K., Kobayashi M., Matsui T., Kodera Y., Sakamoto J. (2009). Individual patient based meta-analysis of lentinan for unresectable/recurrent gastric cancer. Anticancer Res..

[381] Isoda N., Eguchi Y., Nukaya H., Hosho K., Suga Y., Suga T., Nakazawa S., Sugano K. (2009). Clinical efficacy of superfine dispersed lentinan (beta-1, 3-glucan) in patients with hepatocellular carcinoma. Hepato Gastroenterol..

[382] Shimizu K., Watanabe S., Matsuda K., Suga T., Nakazawa S., Shiratori K. (2009). Efficacy of oral administered superfine dispersed lentinan for advanced pancreatic cancer. Hepato Gastroenterol..

[383] Wang H., Cai Y., Zheng Y., Bai Q., Xie D., Yu J. (2017). Efficacy of biological response modifier lentinan with chemotherapy for advanced cancer: A meta-analysis. Cancer Med..

[384] Babu P.D., Subhasree R. (2008). The sacred mushroom “Reishi”—A review. Am. Eurasian J. Bot..

[385] Suarez-Arroyo I.J., Rosario-Acevedo R., Aguilar-Perez A., Clemente P.L., Cubano L.A., Serrano J., Schneider R.J., Martínez-Montemayor M.M. (2013). Anti-tumor effects of *Ganoderma lucidum* (reishi) in inflammatory breast cancer in in vivo and in vitro models. PLoS ONE.

[386] Martínez-Montemayor M.M., Acevedo R.R., Otero-Franqui E., Cubano L.A., Dharmawardhane S.F. (2011). *Ganoderma lucidum* (Reishi) inhibits cancer cell growth and expression of key molecules in inflammatory breast cancer. Nutr. Cancer.

[387] Acevedo-Díaz A., Ortiz-Soto G., Suárez-Arroyo I.J., Zayas-Santiago A., Martínez Montemayor M.M. (2019). *Ganoderma lucidum* extract reduces the motility of breast cancer cells mediated by the RAC–lamellipodin Axis. Nutrients.

[388] Liang C., Tian D., Liu Y., Li H., Zhu J., Li M., Xin M., Xia J. (2019). Review of the molecular mechanisms of *Ganoderma lucidum* triterpenoids: Ganoderic acids A, C2, D, F, DM, X and Y. Eur. J. Med. Chem..

[389] Sohretoglu D., Huang S. (2018). *Ganoderma lucidum* polysaccharides as an anti-cancer agent. Anti-Cancer Agents Med. Chem..

[390] Radwan F.F., Perez J.M., Haque A. (2011). Apoptotic and immune restoration effects of ganoderic acids define a new prospective for complementary treatment of cancer. J. Clin. Cell. Immunol..

[391] Gill B.S., Kumar S. (2016). Ganoderic acid targeting multiple receptors in cancer: In silico and in vitro study. Tumor Biol..

[392] Xia J., Dai L., Wang L., Zhu J. (2020). Ganoderic acid DM induces autophagic apoptosis in non-small cell lung cancer cells by inhibiting the PI3K/Akt/mTOR activity. Chem. Biol. Interact..

[393] Wu G.-S., Lu J.-J., Guo J.-J., Li Y.-B., Tan W., Dang Y.-Y., Zhong Z.-F., Xu Z.-T., Chen X.-P., Wang Y.-T. (2012). Ganoderic acid DM, a natural triterpenoid, induces DNA damage, G1 cell cycle arrest and apoptosis in human breast cancer cells. Fitoterapia.

[394] Chen N.-H., Liu J.-W., Zhong J.-J. (2008). Ganoderic acid Me inhibits tumor invasion through down-regulating matrix metalloproteinases 2/9 gene expression. J. Pharmacol. Sci..

[395] Xu J., Chen F., Wang G., Liu B., Song H., Ma T. (2021). The Versatile Functions of G. Lucidum Polysaccharides and G. Lucidum Triterpenes in Cancer Radiotherapy and Chemotherapy. Cancer Manag. Res..

[396] Pan H., Wang Y., Wang Y., Li M., Li Z., Xu J., Wang X. (2019). *Ganoderma lucidum* polysaccharides induce cytotoxicity in colorectal cancer cells through inducing autophagosome accumulation and inhibiting autophagic flux. FASEB J..

[397] Zhong J., Fang L., Chen R., Xu J., Guo D., Guo C., Guo C., Chen J., Chen C., Wang X. (2021). Polysaccharides from sporoderm-removed spores of *Ganoderma lucidum* induce apoptosis in human gastric cancer cells via disruption of autophagic flux. Oncol. Lett..

[398] Su J., Li D., Chen Q., Li M., Su L., Luo T., Liang D., Lai G., Shuai O., Jiao C. (2018). Anti-breast cancer enhancement of a polysaccharide from spore of *Ganoderma lucidum* with paclitaxel: Suppression on tumor metabolism with gut microbiota reshaping. Front. Microbiol..

[399] Jin H., Song C., Zhao Z., Zhou G. (2020). *Ganoderma Lucidum* Polysaccharide, an Extract from *Ganoderma Lucidum*, Exerts Suppressive Effect on Cervical Cancer Cell Malignancy through Mitigating Epithelial-Mesenchymal and JAK/STAT5 Signaling Pathway. Pharmacology.

[400] Hsu W.-H., Hua W.-J., Qiu W.-L., Tseng A.-J., Cheng H.-C., Lin T.-Y. (2021). WSG, a glucose-enriched polysaccharide from *Ganoderma lucidum*, suppresses tongue cancer cells via inhibition of EGFR-mediated signaling and potentiates cisplatin-induced apoptosis. Int. J. Biol. Macromol..

[401] Cao Y., Xu X., Liu S., Huang L., Gu J. (2018). Ganoderma: A cancer immunotherapy review. Front. Pharmacol..

[402] Zhao R., Chen Q., He Y.-m. (2018). The effect of *Ganoderma lucidum* extract on immunological function and identify its anti-tumor immunostimulatory activity based on the biological network. Sci. Rep..

[403] Chien C.M., Cheng J.-L., Chang W.-T., Tien M.-H., Tsao C.-M., Chang Y.-H., Chang H.-Y., Hsieh J.-F., Wong C.-H., Chen S.-T. (2004). Polysaccharides of *Ganoderma lucidum* alter cell immunophenotypic expression and enhance CD56+ NK-cell cytotoxicity in cord blood. Bioorg. Med. Chem..

[404] Chen H.-S., Tsai Y.-F., Lin S., Lin C.-C., Khoo K.-H., Lin C.-H., Wong C.-H. (2004). Studies on the immuno-modulating and anti-tumor activities of *Ganoderma lucidum* (Reishi) polysaccharides. Bioorg. Med. Chem..

[405] Zhang S., Pang G., Chen C., Qin J., Yu H., Liu Y., Zhang X., Song Z., Zhao J., Wang F. (2019). Effective cancer Immunother. by *Ganoderma lucidum* polysaccharide-gold nanocomposites through dendritic cell activation and memory T cell response. Carbohydr. Polym..

[406] Guo C., Guo D., Fang L., Sang T., Wu J., Guo C., Wang Y., Wang Y., Chen C., Chen J. (2021). *Ganoderma lucidum* polysaccharide modulates gut microbiota and immune cell function to inhibit inflammation and tumorigenesis in colon. Carbohydr. Polym..

[407] Chiu H.-F., Fu H.-Y., Lu Y.-Y., Han Y.-C., Shen Y.-C., Venkatakrishnan K., Golovinskaia O., Wang C.-K. (2017). Triterpenoids and polysaccharide peptides-enriched *Ganoderma lucidum*: A randomized, double-blind placebo-controlled crossover study of its antioxidation and hepatoprotective efficacy in healthy volunteers. Pharm. Biol..

[408] Deng Y., Ma J., Tang D., Zhang Q. (2021). Dynamic biomarkers indicate the immunological benefits provided by Ganoderma spore powder in post-operative breast and lung cancer patients. Clin. Translat. Oncol..

[409] Wu J.-Y., Siu K.-C., Geng P. (2021). Bioactive ingredients and medicinal values of *Grifola frondosa* (Maitake). Foods.

[410] De Silva D.D., Rapior S., Fons F., Bahkali A.H., Hyde K.D. (2012). Medicinal mushrooms in supportive cancer therapies: An approach to anti-cancer effects and putative mechanisms of action. Fungal Divers..

[411] Alonso E.N., Orozco M., Nieto A.E., Balogh G.A. (2013). Genes related to suppression of malignant phenotype induced by Maitake D-Fraction in breast cancer cells. J. Med. Food.

[412] Alonso E.N., Ferronato M.J., Fermento M.E., Gandini N.A., Romero A.L., Guevara J.A., Facchinetti M.M., Curino A.C. (2018). Antitumoral and antimetastatic activity of Maitake D-Fraction in triple-negative breast cancer cells. Oncotarget.

[413] Lin C.-H., Chang C.-Y., Lee K.-R., Lin H.-J., Lin W.-C., Chen T.-H., Wan L. (2016). Cold-water extracts of *Grifola frondosa* and its purified active fraction inhibit hepatocellular carcinoma in vitro and in vivo. Exp. Biol. Med..

[414] Fullerton S., Samadi A., Tortorelis D., Choudhury M., Mallouh C., Tazaki H., Konno S. (2000). Induction of apoptosis in human prostatic cancer cells with beta-glucan (Maitake mushroom polysaccharide). Mol. Urology.

[415] Alexander B., Fishman A.I., Eshghi M., Choudhury M., Konno S. (2013). Induction of cell death in renal cell carcinoma with combination of D-fraction and vitamin C. Integr. Cancer Ther..

[416] Roldan-Deamicis A., Alonso E., Brie B., Braico D.A., Balogh G.A. (2016). Maitake Pro4X has anti-cancer activity and prevents oncogenesis in BALB c mice. Cancer Med..

[417] Kodama N., Komuta K., Sakai N., Nanba H. (2002). Effects of D-Fraction, a polysaccharide from *Grifola frondosa* on tumor growth involve activation of NK cells. Biol. Pharm. Bull..

[418] Zhao F., Guo Z., Zhang Y., Song L., Ma L., Zhao J. (2021). Anti-tumor and immunomodulatory effects of *Grifola frondosa* polysaccharide combined with vitamin C on Heps-bearing mice: Based on inducing apoptosis and autophagy. J. Funct. Foods.

[419] Masuda Y., Nakayama Y., Tanaka A., Naito K., Konishi M. (2017). Antitumor activity of orally administered maitake α-glucan by stimulating antitumor immune response in murine tumor. PLoS ONE.

[420] Zhao F., Guo Z., Ma Z.-R., Ma L.-L., Zhao J. (2021). Antitumor activities of *Grifola frondosa* (Maitake) polysaccharide: A meta-analysis based on preclinical evidence and quality assessment. J. Ethnopharmacol..

[421] Kodama N., Komuta K., Nanba H. (2002). Can maitake MD-fraction aid cancer patients?. Altern. Med. Rev..

[422] Kodama N., Komuta K., Nanba H. (2003). Effect of Maitake (*Grifola frondosa*) D-Fraction on the activation of NK cells in cancer patients. J. Med. Food.

[423] He Y., Zhang L., Wang H. (2019). The biological activities of the antitumor drug *Grifola frondosa* polysaccharide. Prog. Mol. Biol. Translat. Sci..

[424] Zhou X., Gong Z., Su Y., Lin J., Tang K. (2009). Cordyceps fungi: Natural products, pharmacological functions and developmental products. J. Pharm. Pharmacol..

[425] Yue K., Ye M., Zhou Z., Sun W., Lin X. (2013). The genus Cordyceps: A chemical and pharmacological review. J. Pharm. Pharmacol..

[426] Jędrejko K.J., Lazur J., Muszyńska B. (2021). *Cordyceps militaris*: An Overview of Its Chemical Constituents in Relation to Biological Activity. Foods.

[427] Li J., Cai H., Sun H., Qu J., Zhao B., Hu X., Li W., Qian Z., Yu X., Kang F. (2020). Extracts of *Cordyceps sinensis* inhibit breast cancer growth through promoting M1 macrophage polarization via NF-κB pathway activation. J. Ethnopharmacol..

[428] Seo H., Song J., Kim M., Han D.-W., Park H.-J., Song M. (2018). *Cordyceps militaris* grown on germinated soybean suppresses KRAS-driven colorectal cancer by inhibiting the RAS/ERK pathway. Nutrients.

[429] Jeong M.-K., Yoo H.-S., Kang I.-C. (2019). The extract of *Cordyceps militaris* inhibited the proliferation of cisplatin-resistant a549 lung cancer cells by downregulation of H-Ras. J. Med. Food.

[430] Jo E., Jang H.-J., Shen L., Yang K.E., Jang M.S., Huh Y.H., Yoo H.-S., Park J., Jang I.S., Park S.J. (2020). *Cordyceps militaris* Exerts Anticancer Effect on Non–Small Cell Lung Cancer by Inhibiting Hedgehog Signaling via Suppression of TCTN3. Integr. Cancer Ther..

[431] Yoon S.Y., Park S.J., Park Y.J. (2018). The anticancer properties of cordycepin and their underlying mechanisms. Int. J. Mol. Sci..

[432] Fishman P., Bar-Yehuda S., Synowitz M., Powell J., Klotz K., Gessi S., Borea P. (2009). Adenosine receptors and cancer. Adenosine Recept. Health Dis..

[433] Cao H.-L., Liu Z.-J., Chang Z. (2017). Cordycepin induces apoptosis in human bladder cancer cells via activation of A3 adenosine receptors. Tumor Biol..

[434] Nakamura K., Yoshikawa N., Yamaguchi Y., Kagota S., Shinozuka K., Kunitomo M. (2006). Antitumor effect of cordycepin (3′-deoxyadenosine) on mouse melanoma and lung carcinoma cells involves adenosine A3 receptor stimulation. Anticancer Res..

[435] Yoshikawa N., Yamada S., Takeuchi C., Kagota S., Shinozuka K., Kunitomo M., Nakamura K. (2008). Cordycepin (3′-deoxyadenosine) inhibits the growth of B16-BL6 mouse melanoma cells through the stimulation of adenosine A3 receptor followed by glycogen synthase kinase-3β activation and cyclin D1 suppression. Naunyn Schmiedeberg’s Arch. Pharmacol..

[436] Zhao X., Guan J.-L. (2011). Focal adhesion kinase and its signaling pathways in cell migration and angiogenesis. Adv. Drug Deliv. Rev..

[437] Yao W.-L., Ko B.-S., Liu T.-A., Liang S.-M., Liu C.-C., Lu Y.-J., Tzean S.-S., Shen T.-L., Liou J.-Y. (2014). Cordycepin suppresses integrin/FAK signaling and epithelial-mesenchymal transition in hepatocellular carcinoma. Anti Cancer Agents Med. Chem..

[438] Jin Y., Meng X., Qiu Z., Su Y., Yu P., Qu P. (2018). Anti-tumor and anti-metastatic roles of cordycepin, one bioactive compound of *Cordyceps militaris*. Saudi J. Biol. Sci..

[439] Lee S.Y., Debnath T., Kim S.-K., Lim B.O. (2013). Anti-cancer effect and apoptosis induction of cordycepin through DR3 pathway in the human colonic cancer cell HT-29. Food Chem. Toxicol..

[440] Lee H.H., Kim S.O., Kim G.-Y., Moon S.-K., Kim W.-J., Jeong Y.K., Yoo Y.H., Choi Y.H. (2014). Involvement of autophagy in cordycepin-induced apoptosis in human prostate carcinoma LNCaP cells. Environ. Toxicol. Pharmacol..

[441] Chang M.-M., Hong S.-Y., Yang S.-H., Wu C.-C., Wang C.-Y., Huang B.-M. (2020). Anti-cancer effect of cordycepin on FGF9-induced testicular tumorigenesis. Int. J. Mol. Sci..

[442] Wang J., Chen H., Li W., Shan L. (2021). Cordyceps acid alleviates lung cancer in nude mice. J. Biochem. Mol. Toxicol..

[443] Ka Wai Lee S., Kwok Wong C., Kai Kong S., Nam Leung K., Wai Kei Lam C. (2006). Immunomodulatory activities of HERBSnSENSES™ Cordyceps—in vitro and in vivo studies. Immunopharmacol. Immunotoxicol..

[444] Wang M., Meng X.Y., Le Yang R., Qin T., Wang X.Y., Zhang K.Y., Fei C.Z., Li Y., liang Hu Y., Xue F.Q. (2012). *Cordyceps militaris* polysaccharides can enhance the immunity and antioxidation activity in immunosuppressed mice. Carbohydr. Polym..

[445] Das G., Shin H.-S., Leyva-Gómez G., Prado-Audelo M.L.D., Cortes H., Singh Y.D., Panda M.K., Mishra A.P., Nigam M., Saklani S. (2021). Cordyceps spp.: A review on its immune-stimulatory and other biological potentials. Front. Pharmacol..

[446] Qi W., Zhou X., Wang J., Zhang K., Zhou Y., Chen S., Nie S., Xie M. (2020). *Cordyceps sinensis* polysaccharide inhibits colon cancer cells growth by inducing apoptosis and autophagy flux blockage via mTOR signaling. Carbohydr. Polym..

[447] Borodina I., Kenny L.C., McCarthy C.M., Paramasivan K., Pretorius E., Roberts T.J., van der Hoek S.A., Kell D.B. (2020). The biology of ergothioneine, an antioxidant nutraceutical. Nutr. Res. Rev..

[448] D’Onofrio N., Martino E., Balestrieri A., Mele L., Cautela D., Castaldo D., Balestrieri M.L. (2022). Diet-derived ergothioneine induces necroptosis in colorectal cancer cells by activating the SIRT3/MLKL pathway. FEBS Lett..

[449] Yoshida S., Shime H., Matsumoto M., Kasahara M., Seya T. (2019). Anti-oxidative amino acid L-ergothioneine modulates the tumor microenvironment to facilitate adjuvant vaccine immunotherapy. Front. Immunol..

[450] Smith E., Ottosson F., Hellstrand S., Ericson U., Orho-Melander M., Fernandez C., Melander O. (2020). Ergothioneine is associated with reduced mortality and decreased risk of cardiovascular disease. Heart.

[451] Winkels R.M., Van Brakel L., Van Baar H., Beelman R.B., Van Duijnhoven F.J., Geijsen A., Van Halteren H.K., Hansson B.M., Richie J.P., Sun D. (2020). Are Ergothioneine Levels in Blood Associated with Chronic Peripheral Neuropathy in Colorectal Cancer Patients Who Underwent Chemotherapy?. Nutr. Cancer.

[452] Zhao Y., Zheng W. (2021). Deciphering the antitumoral potential of the bioactive metabolites from medicinal mushroom *Inonotus obliquus*. J. Ethnopharmacol..

[453] Arata S., Watanabe J., Maeda M., Yamamoto M., Matsuhashi H., Mochizuki M., Kagami N., Honda K., Inagaki M. (2016). Continuous intake of the Chaga mushroom (*Inonotus obliquus*) aqueous extract suppresses cancer progression and maintains body temperature in mice. Heliyon.

[454] Lee M.-G., Kwon Y.-S., Nam K.-S., Kim S.Y., Hwang I.H., Kim S., Jang H. (2021). Chaga mushroom extract induces autophagy via the AMPK-mTOR signaling pathway in breast cancer cells. J. Ethnopharmacol..

[455] Zhang Q., Wang J., He H., Liu H., Yan X., Zou K. (2014). Trametenolic Acid B Reverses Multidrug Resistance in Breast Cancer Cells Through Regulating the Expression Level of P-Glycoprotein. Phytother. Res..

[456] Lu Y., Jia Y., Xue Z., Li N., Liu J., Chen H. (2021). Recent developments in *Inonotus obliquus* (*Chaga mushroom*) polysaccharides: Isolation, structural characteristics, biological activities and application. Polymers.

[457] Kim Y.O., Park H.W., Kim J.H., Lee J.Y., Moon S.H., Shin C.S. (2006). Anti-cancer effect and structural characterization of endo-polysaccharide from cultivated mycelia of *Inonotus obliquus*. Life Sci..

[458] Jiang S., Shi F., Lin H., Ying Y., Luo L., Huang D., Luo Z. (2020). *Inonotus obliquus* polysaccharides induces apoptosis of lung cancer cells and alters energy metabolism via the LKB1/AMPK axis. Int. J. Biol. Macromol..

[459] Pei J., Velu P., Zareian M., Feng Z., Vijayalakshmi A. (2021). Effects of Syringic Acid on Apoptosis, Inflammation, and AKT/mTOR Signaling Pathway in Gastric Cancer Cells. Front. Nutr..

[460] Sung B., Pandey M.K., Nakajima Y., Nishida H., Konishi T., Chaturvedi M.M., Aggarwal B.B. (2008). Identification of a novel blocker of IκBα kinase activation that enhances apoptosis and inhibits proliferation and invasion by suppressing nuclear factor-κB. Mol. Cancer Ther..

[461] Zhang X., Bao C., Zhang J. (2018). Inotodiol suppresses proliferation of breast cancer in rat model of type 2 diabetes mellitus via downregulation of β-catenin signaling. Biomed. Pharmacother..

[462] Zhang S.-D., Yu L., Wang P., Kou P., Li J., Wang L.-T., Wang W., Yao L.-P., Zhao X.-H., Fu Y.-J. (2019). Inotodiol inhibits cells migration and invasion and induces apoptosis via p53-dependent pathway in HeLa cells. Phytomedicine.

[463] Sarfraz A., Rasul A., Sarfraz I., Shah M.A., Hussain G., Shafiq N., Masood M., Adem Ş., Sarker S.D., Li X. (2020). Hispolon: A natural polyphenol and emerging cancer killer by multiple cellular signaling pathways. Environ. Res..

[464] Masood M., Rasul A., Sarfraz I., Jabeen F., Liu S., Liu X., Wei W., Li J., Li X. (2019). Hispolon induces apoptosis against prostate DU145 cancer cells via modulation of mitochondrial and STAT3 pathways. Pak. J. Pharm. Sci.

[465] Al Saqr A., Majrashi M., Alrbyawi H., Govindarajulu M., Fujihashi A., Gottumukkala S., Poudel I., Arnold R.D., Babu R.J., Dhanasekaran M. (2020). Elucidating the anti-melanoma effect and mechanisms of Hispolon. Life Sci..

[466] Hsin M.-C., Hsieh Y.-H., Wang P.-H., Ko J.-L., Hsin I.-L., Yang S.-F. (2017). Hispolon suppresses metastasis via autophagic degradation of cathepsin S in cervical cancer cells. Cell Death Dis..

[467] Palkina K.A., Ipatova D.A., Shakhova E.S., Balakireva A.V., Markina N.M. (2021). Ther. Potential of Hispidin—Fungal and Plant Polyketide. J. Fungi.

[468] Lv L.-X., Zhou Z.-X., Zhou Z., Zhang L.-J., Yan R., Zhao Z., Yang L.-Y., Bian X.-Y., Jiang H.-Y., Li Y.-D. (2017). Hispidin induces autophagic and necrotic death in SGC-7901 gastric cancer cells through lysosomal membrane permeabilization by inhibiting tubulin polymerization. Oncotarget.

[469] Chandimali N., JIN W.Y., KWON T. (2018). Combination effects of hispidin and gemcitabine via inhibition of stemness in pancreatic cancer stem cells. Anticancer Res..

[470] Ahn W.-S., Kim D.-J., Chae G.-T., Lee J.-M., Bae S.-M., Sin J.-I., Kim Y.-W., Namkoong S.-E., Lee I. (2004). Natural killer cell activity and quality of life were improved by consumption of a mushroom extract, *Agaricus blazei* Murill Kyowa, in gynecological cancer patients undergoing chemotherapy. Int. J. Gynecol. Cancer.

[471] Hetland G., Tangen J.-M., Mahmood F., Mirlashari M.R., Nissen-Meyer L.S.H., Nentwich I., Therkelsen S.P., Tjønnfjord G.E., Johnson E. (2020). Antitumor, anti-inflammatory and antiallergic effects of *Agaricus blazei* mushroom extract and the related medicinal Basidiomycetes mushrooms, *Hericium erinaceus* and *Grifola frondosa*: A review of preclinical and clinical studies. Nutrients.

[472] Tangen J.-M., Tierens A., Caers J., Binsfeld M., Olstad O.K., Trøseid A.-M.S., Wang J., Tjønnfjord G.E., Hetland G. (2015). Immunomodulatory effects of the *Agaricus blazei* Murrill-based mushroom extract AndoSan in patients with multiple myeloma undergoing high dose chemotherapy and autologous stem cell transplantation: A randomized, double blinded clinical study. Biomed Res. Int..

[473] Lee S.H., Hwang H.K., Kang C.M., Lee W.J. (2019). Potential impact of *Phellinus linteus* on adherence to adjuvant treatment after curative resection of pancreatic ductal adenocarcinoma: Outcomes of a propensity score–matched analysis. Integr. Cancer Ther..

[474] Chen W., Tan H., Liu Q., Zheng X., Zhang H., Liu Y., Xu L. (2019). A review: The bioactivities and pharmacological applications of *Phellinus linteus*. Molecules.

[475] Zhang C.-C., Cao C.-Y., Kubo M., Harada K., Yan X.-T., Fukuyama Y., Gao J.-M. (2017). Chemical constituents from *Hericium erinaceus* promote neuronal survival and potentiate neurite outgrowth via the TrkA/Erk1/2 pathway. Int. J. Mol. Sci..

[476] Tung S.-Y., Lee K.-C., Lee K.-F., Yang Y.-L., Huang W.-S., Lee L.-Y., Chen W.-P., Chen C.-C., Teng C.-C., Shen C.-H. (2021). Apoptotic mechanisms of gastric cancer cells induced by isolated erinacine S through epigenetic histone H3 methylation of FasL and TRAIL. Food Funct..

[477] Kuo H.-C., Kuo Y.-R., Lee K.-F., Hsieh M.-C., Huang C.-Y., Hsieh Y.-Y., Lee K.-C., Kuo H.-L., Lee L.-Y., Chen W.-P. (2017). A comparative proteomic analysis of Erinacine A’s inhibition of gastric cancer cell viability and invasiveness. Cell. Physiol. Biochem..

[478] Lee K.-C., Lee K.-F., Tung S.-Y., Huang W.-S., Lee L.-Y., Chen W.-P., Chen C.-C., Teng C.-C., Shen C.-H., Hsieh M.-C. (2019). Induction apoptosis of erinacine a in human colorectal cancer cells involving the expression of TNFR, fas, and fas ligand via the JNK/p300/p50 signaling pathway with histone acetylation. Front. Pharmacol..

[479] TSANG K.W., Lam C., Yan C., Mak J., Ooi G., Ho J., Lam B., Man R., Sham J., Lam W. (2003). Coriolus versicolor polysaccharide peptide slows progression of advanced non-small cell lung cancer. Resp. Med..

[480] Ohwada S., Ikeya T., Yokomori T., Kusaba T., Roppongi T., Takahashi T., Nakamura S., Kakinuma S., Iwazaki S., Ishikawa H. (2004). Adjuvant immunochemotherapy with oral Tegafur/Uracil plus PSK in patients with stage II or III colorectal cancer: A randomised controlled study. Br. J. Cancer.

[481] Li J.W.-H., Vederas J.C. (2009). Drug discovery and natural products: End of an era or an endless frontier?. Science.

[482] Zhao Q., Luan X., Zheng M., Tian X.-H., Zhao J., Zhang W.-D., Ma B.-L. (2020). Synergistic mechanisms of constituents in herbal extracts during intestinal absorption: Focus on natural occurring nanoparticles. Pharmaceutics.

[483] Jürgenliemk G., Nahrstedt A. (2003). Dissolution, solubility and cooperativity of phenolic compounds from *Hypericum perforatum* L. in aqueous systems. Int. J. Pharm. Sci..

[484] Cai T.-Y., Zhang Y.-R., Ji J.-B., Xing J. (2017). Investigation of the component in Artemisia annua L. leading to enhanced antiplasmodial potency of artemisinin via regulation of its metabolism. J. Ethnopharmacol..

[485] Gröning R., Breitkreutz J., Müller R. (2003). Physico-chemical interactions between extracts of *Hypericum perforatum* L. and drugs. Eur. J. Pharm. Biopharm..

[486] Zhuang Y., Yan J., Zhu W., Chen L., Liang D., Xu X. (2008). Can the aggregation be a new approach for understanding the mechanism of Tradit. Chin. Medicine?. J. Ethnopharmacol..

[487] Mukherjee P.K., Harwansh R.K., Bhattacharyya S. (2015). Bioavailability of herbal products: Approach toward improved pharmacokinetics. Evidence-Based Validation of Herbal Medicine.

[488] Phansalkar P.S., Zhang Z., Verenich S., Gerk P.M. (2020). Pharmacokinetics and bioavailability enhancement of natural products. Natural Products for Cancer Chemoprevention.

[489] Khedekar K., Mittal S. (2013). Self emulsifying drug delivery system: A review. Int. J. Pharm. Sci. Res..

[490] Singh N., Rai S., Bhattacharya S. (2021). A Conceptual Analysis of solid Self-emulsifying drug Delivery System and its Associate Patents for the Treatment of Cancer. Recent Pat. Nanotechnol.

[491] Murugesan M.P., Ratnam M.V., Mengitsu Y., Kandasamy K. (2021). Evaluation of anti-cancer activity of phytosomes formulated from *Aloe vera* extract. Mat. Today Proc..

[492] Flaig T.W., Gustafson D.L., Su L.-J., Zirrolli J.A., Crighton F., Harrison G.S., Pierson A.S., Agarwal R., Glodé L.M. (2007). A phase I and pharmacokinetic study of silybin-phytosome in prostate cancer patients. Investig. New Drugs.

[493] Flaig T.W., Glodé M., Gustafson D., van Bokhoven A., Tao Y., Wilson S., Su L.J., Li Y., Harrison G., Agarwal R. (2010). A study of high-dose oral silybin-phytosome followed by prostatectomy in patients with localized prostate cancer. Prostate.

[494] Alhakamy N.A., Fahmy U.A., Eldin S.M.B., Ahmed O.A., Aldawsari H.M., Okbazghi S.Z., Alfaleh M.A., Abdulaal W.H., Alamoudi A.J., Mady F.M. (2021). Scorpion Venom-Functionalized Quercetin Phytosomes for Breast Cancer Management: In Vitro Response Surface Optimization and Anticancer Activity against MCF-7 Cells. Polymers.

[495] Alhakamy N.A., Badr-Eldin S.M., Fahmy U.A., Alruwaili N.K., Awan Z.A., Caruso G., Alfaleh M.A., Alaofi A.L., Arif F.O., Ahmed O.A. (2020). Thymoquinone-loaded soy-phospholipid-based phytosomes exhibit anticancer potential against human lung cancer cells. Pharmaceutics.

[496] Muhamad N., Plengsuriyakarn T., Na-Bangchang K. (2018). Application of active targeting nanoparticle delivery system for chemoTher. drugs and traditional/herbal medicines in cancer therapy: A systematic review. Int. J. Nanomed..

[497] Rehman M.U., Khan A., Imtiyaz Z., Ali S., Makeen H.A., Rashid S., Arafah A. (2022). Current Nano-Therapeutic Approaches Ameliorating Inflammation in Cancer Progression. Semin. Cancer Biol..

[498] Salunkhe R., Gadgoli C., Naik A., Patil N. (2021). Pharmacokinetic Profile and Oral Bioavailability of Diosgenin, Charantin, and Hydroxychalcone from a Polyherbal Formulation. Front. Pharmacol..

[499] Peterson B., Weyers M., Steenekamp J.H., Steyn J.D., Gouws C., Hamman J.H. (2019). Drug bioavailability enhancing agents of natural origin (bioenhancers) that modulate drug membrane permeation and pre-systemic metabolism. Pharmaceutics.

[500] Apolone G., Joppi R., Bertele V., Garattini S. (2005). Ten years of marketing approvals of anticancer drugs in Europe: Regulatory policy and guidance documents need to find a balance between different pressures. Br. J. Cancer.

[501] Zhang J., Onakpoya I.J., Posadzki P., Eddouks M. (2015). The safety of herbal medicine: From prejudice to evidence. Evid. Based Complement. Alternat. Med..

[502] Mazzanti G., Menniti-Ippolito F., Moro P.A., Cassetti F., Raschetti R., Santuccio C., Mastrangelo S. (2009). Hepatotoxicity from green tea: A review of the literature and two unpublished cases. Eur. J. Clin. Pharmacol..

[503] Hu J., Webster D., Cao J., Shao A. (2018). The safety of green tea and green tea extract consumption in adults–results of a systematic review. Regul. Toxicol. Pharmacol..

[504] Schmidt M., Schmitz H.-J., Baumgart A., Guedon D., Netsch M., Kreuter M.-H., Schmidlin C., Schrenk D. (2005). Toxicity of green tea extracts and their constituents in rat hepatocytes in primary culture. Food Chem. Toxicol..

[505] Additives E.P.o.F., Food N.S.a.t., Younes M., Aggett P., Aguilar F., Crebelli R., Dusemund B., Filipič M., Frutos M.J., Galtier P. (2018). Scientific opinion on the safety of green tea catechins. EFSA J..

[506] Fasinu P.S., Bouic P.J., Rosenkranz B. (2012). An overview of the evidence and mechanisms of herb–drug interactions. Front. Pharmacol..

[507] Yadav P., Mahour K., Kumar A. (2011). Standardization and evaluation of herbal drug formulations. J. Adv. Lab. Res. Biol..

[508] Bijauliya R.K., Alok S., Chanchal D.K., Kumar M. (2017). A comprehensive review on standardization of herbal drugs. Int. J. Pharm. Sci. Res..

[509] Govindaraghavan S., Sucher N.J. (2015). Quality assessment of medicinal herbs and their extracts: Criteria and prerequisites for consistent safety and efficacy of herbal medicines. Epilepsy Behav..

[510] Wang J., Guo Y., Li G.L. (2016). Current Status of Standardization of Traditional Chinese Medicine in China. Evid. Based Complement. Altern. Med..

[511] Downer S., Berkowitz S.A., Harlan T.S., Olstad D.L., Mozaffarian D. (2020). Food is medicine: Actions to integrate food and nutrition into healthcare. BMJ.

[512] Chen X., Yue W., Tian L., Li N., Chen Y., Zhang L., Chen J. (2021). A plant-based medicinal food inhibits the growth of human gastric carcinoma by reversing epithelial–mesenchymal transition via the canonical Wnt/β-catenin signaling pathway. BMC Complement. Med. Ther..

