# Supplementary material for: How Should the Worldwide Knowledge of Traditional Cancer Healing Be Integrated with Herbs and Mushrooms into Modern Molecular Pharmacology?
Source: Pharmaceuticals (Basel). 2022 Jul 14;15(7):868. doi: 10.3390/ph15070868 (PMC9320176; doi:10.3390/ph15070868)
Supplement: Supplementary file 1 [file pharmaceuticals-15-00868-s001.zip › Figure S1.pdf]

Hypericum perforatum

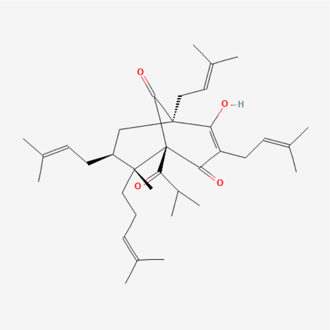

Hyperforin

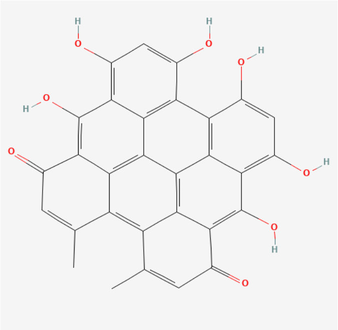

Hypericin

Betula pubescens

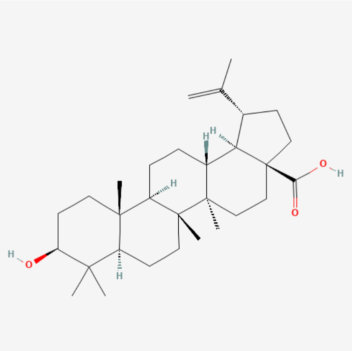

Betulinic acid

Glycyrrhiza glabra

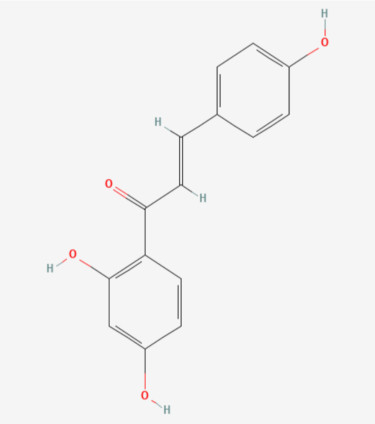

Isoliquiritigenin

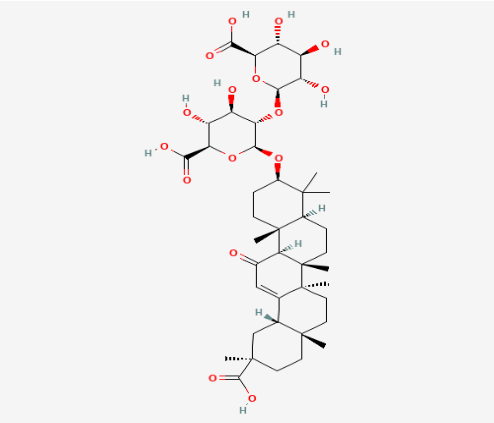

Glycyrrhizin

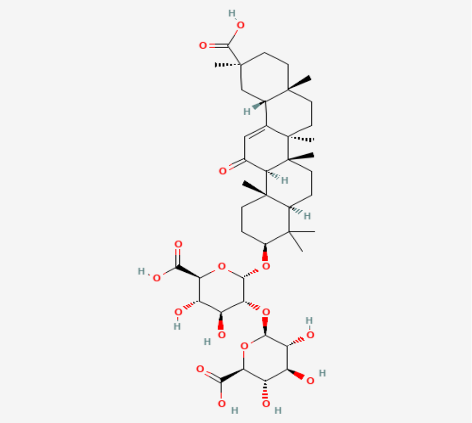

Glycyrrhizinic acid

Cephalotaxus harringtonia

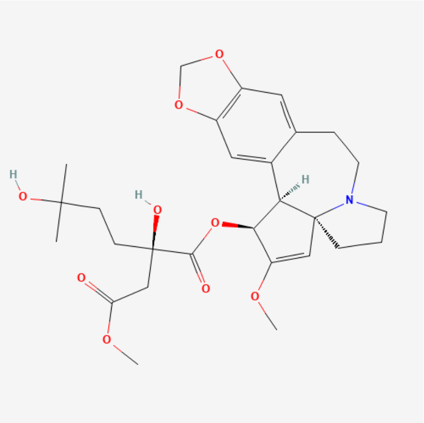

Harringtonin

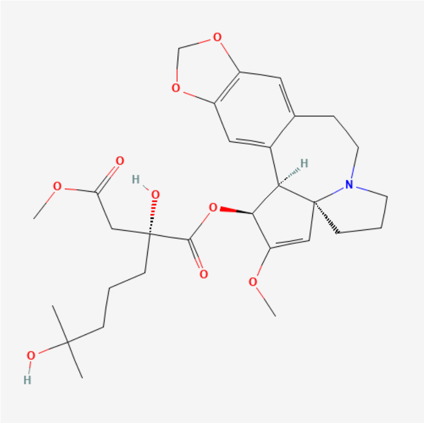

Homoharringtonin

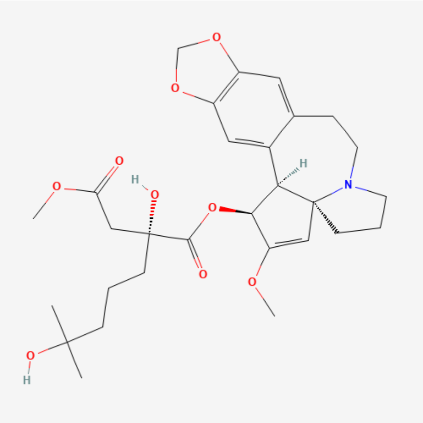

Omacetaxine

Scutellaria barbata

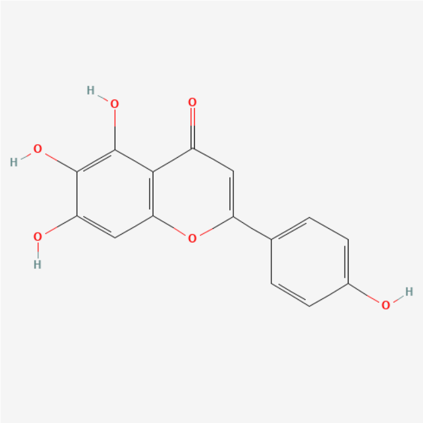

Scutellarein

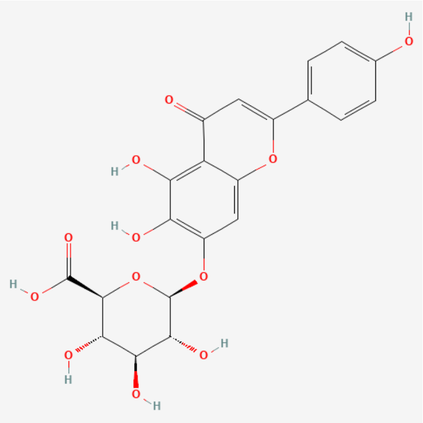

Scutellarin

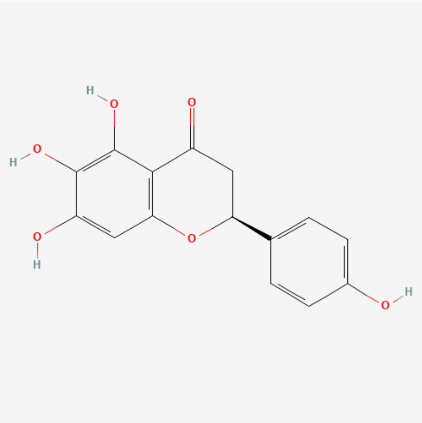

Carthamidin

Withania somnifera

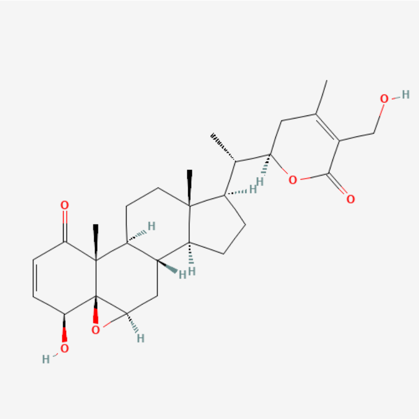

Whithaferin A

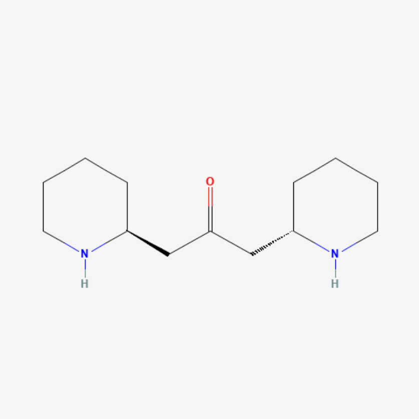

Anaferine

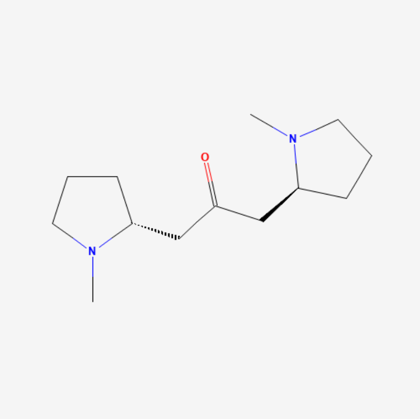

Cuseohygrine

Guera senegalis

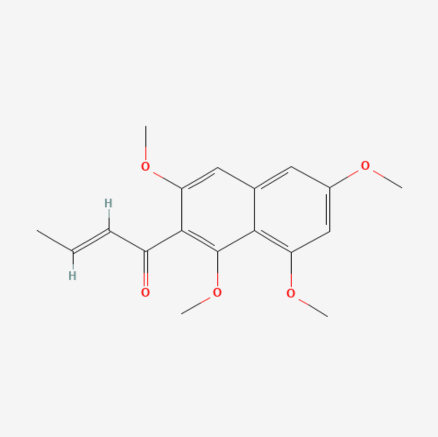

Guieranone A

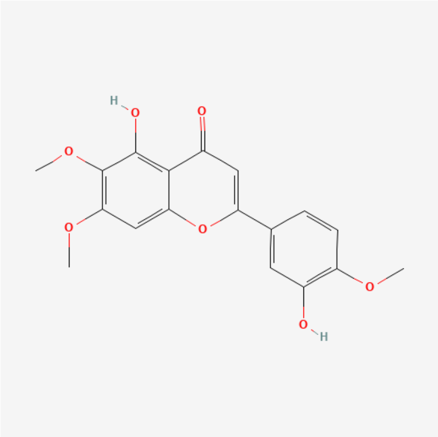

Eupatorin

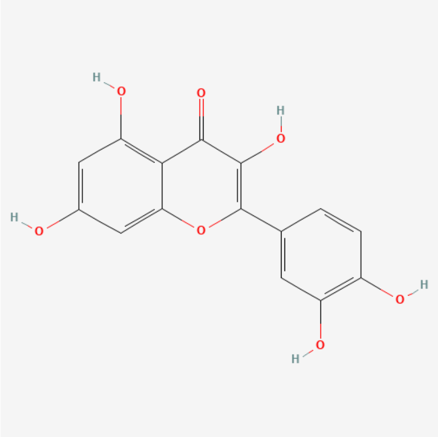

Quercetin

Acacia nilotica

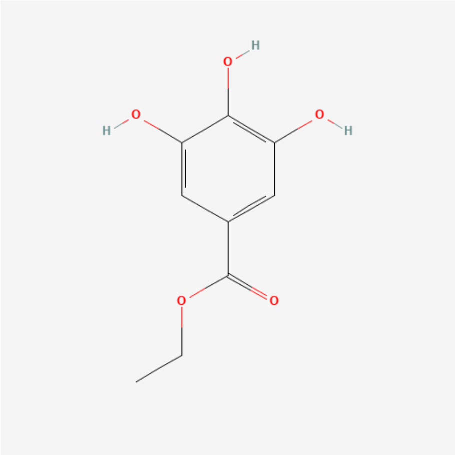

Ethylgallate

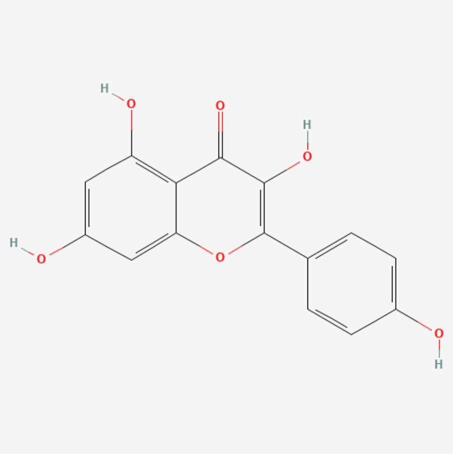

Kaempferol

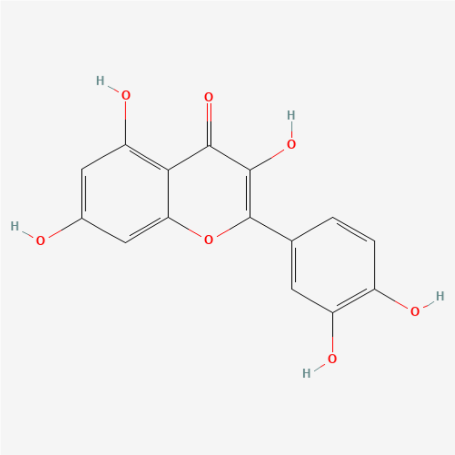

Quercetin

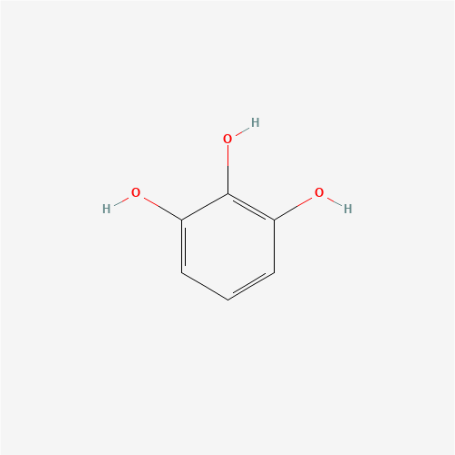

Pyrogallol

Xanthium spinosum

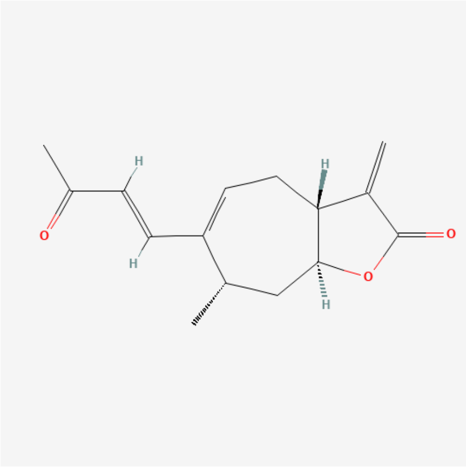

Xanthatin

Oldenlandia diffusa

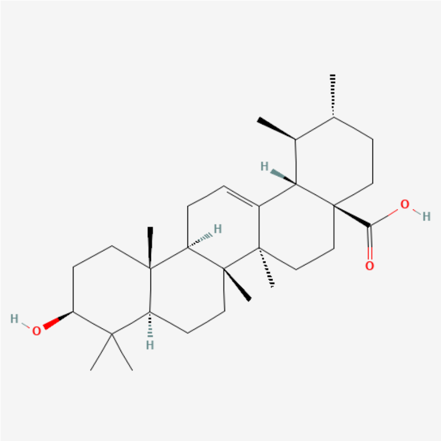

Ursolic acid

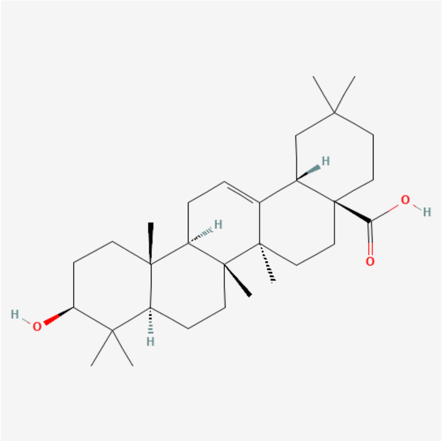

Oleanolic acid

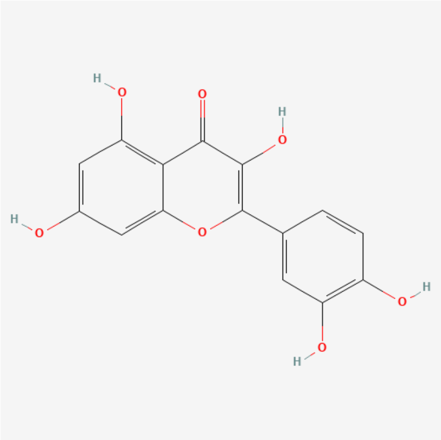

Quercetin

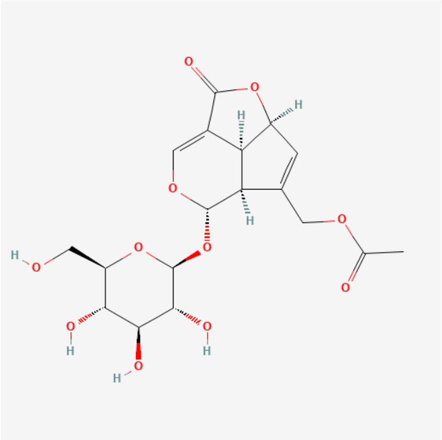

Asperuloside

Curcuma longa

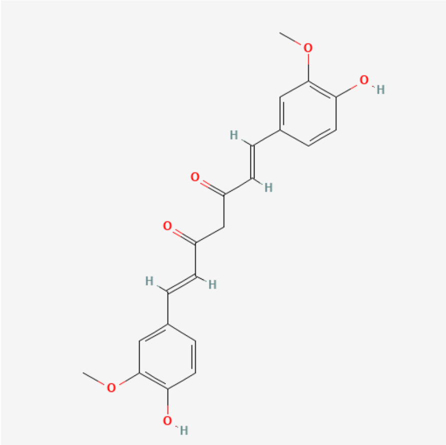

Curcumin

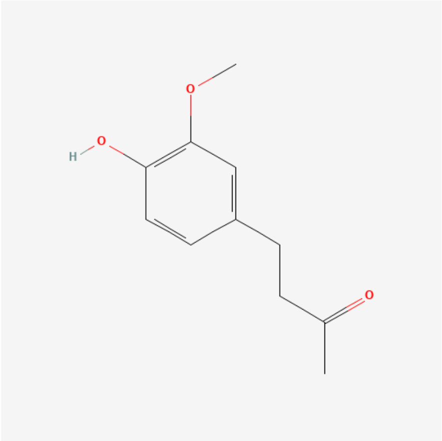

Zingiberone

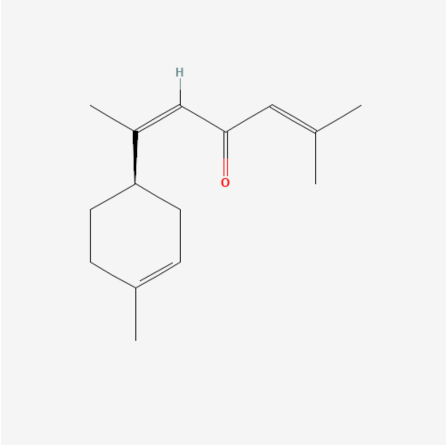

Atlantone

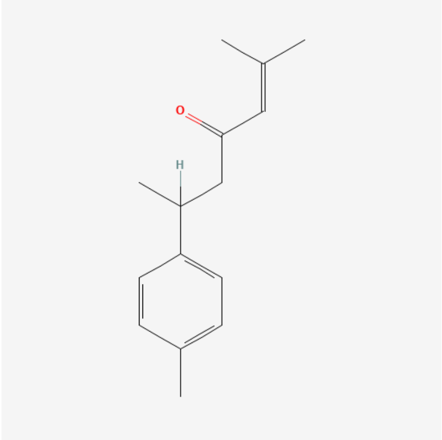

Tumerone

Silybum marianum

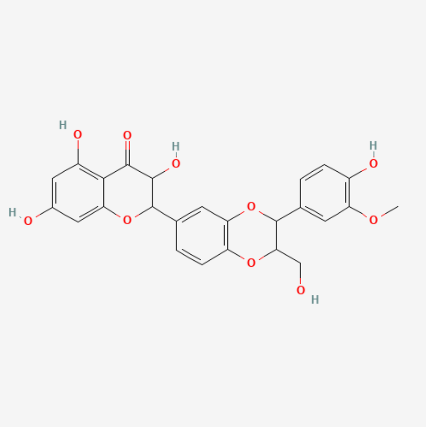

Symilarin

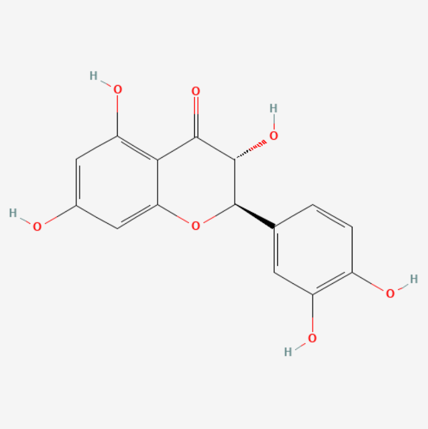

Taxifolin

Capsicum frutenese

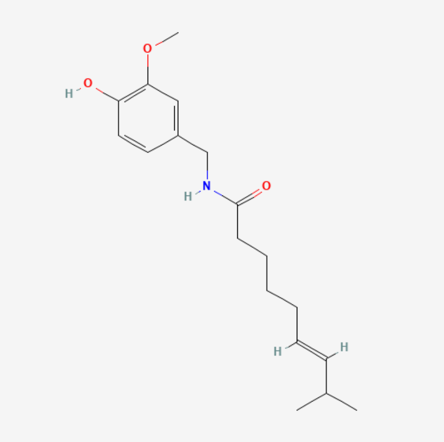

Capsaicin

Aloe vera and Aloe arborescence

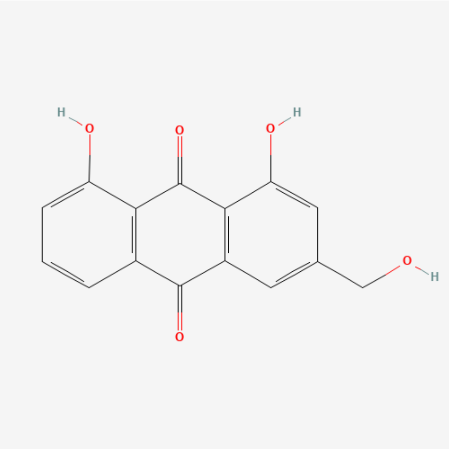

Aloe-emodin

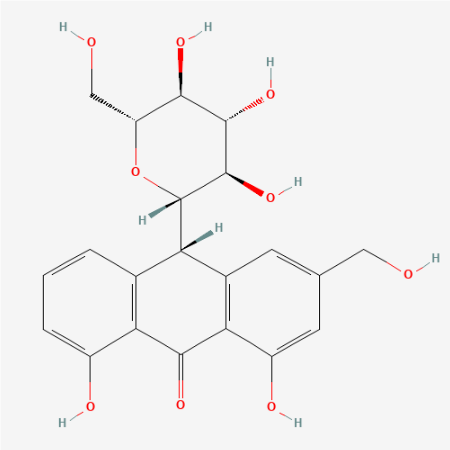

Barbaloin

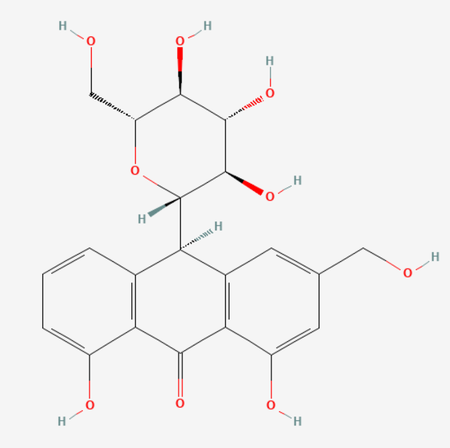

Isobarbaloin

Panax quinquefolius

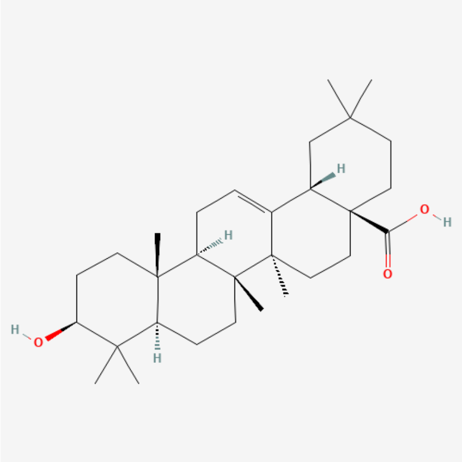

Oleanolic acid

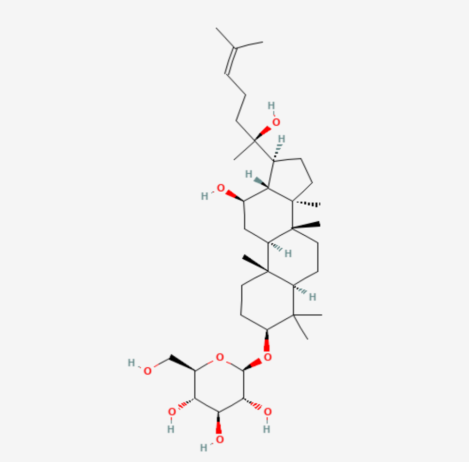

Ginsenoside-Rh2

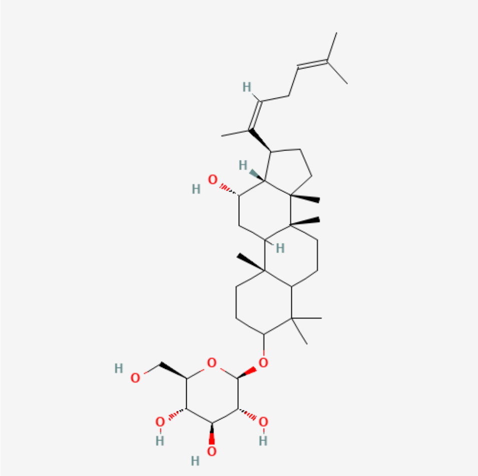

Ginsenoside-Rh3

Tabebuia impetiginosa

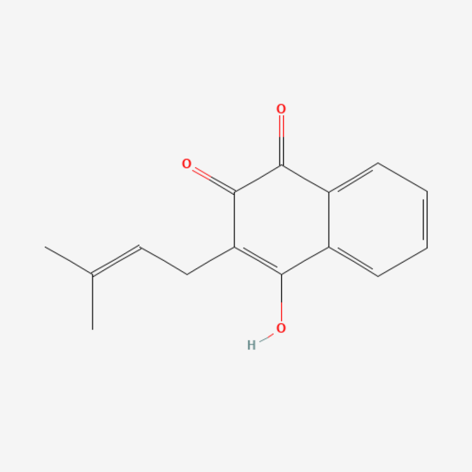

Lapachol

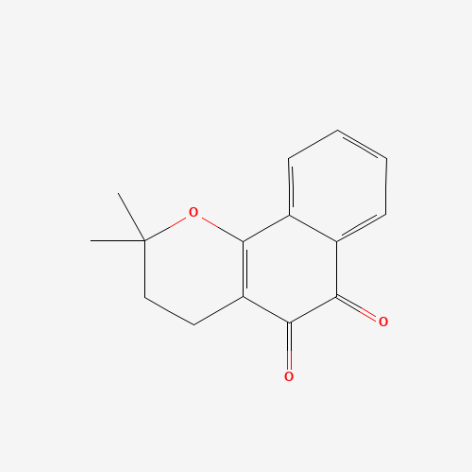

β-lapachone

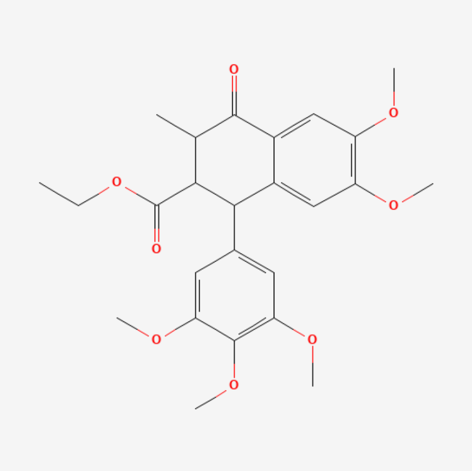

Lignan

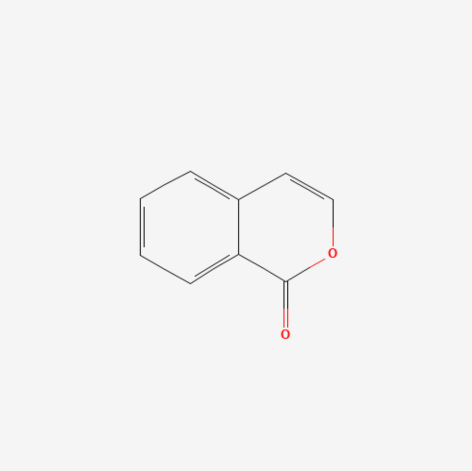

Isocoumarin

Combretum caffrum

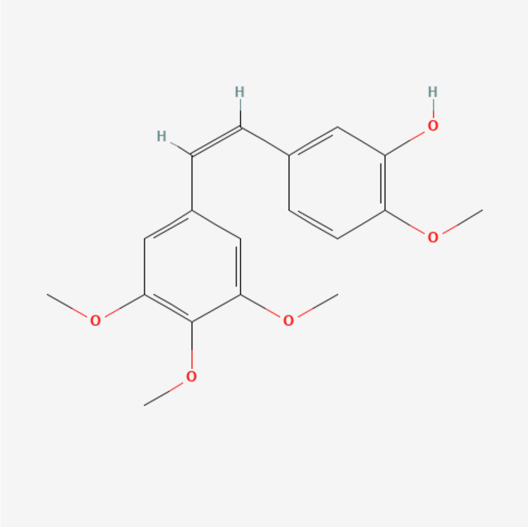

Combretastatin A4

Chaga (Inonotus obliquus)

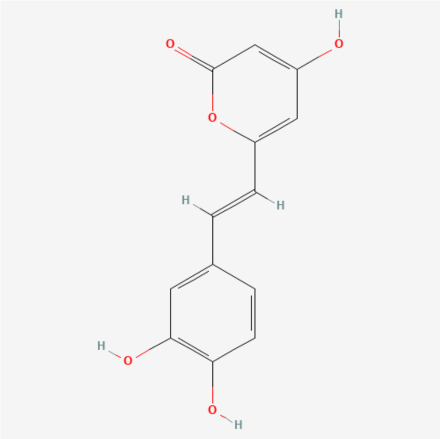

Hispidin

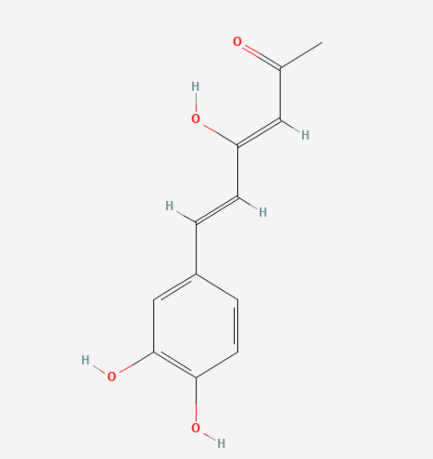

Hispolon

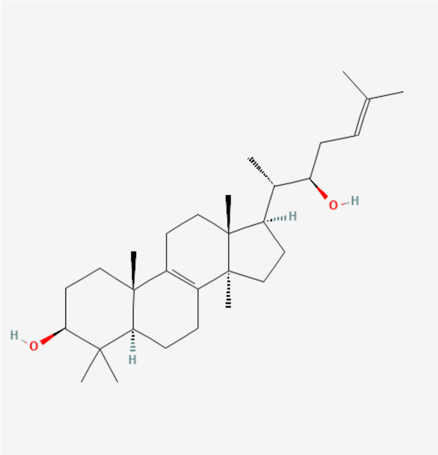

Inotodiol

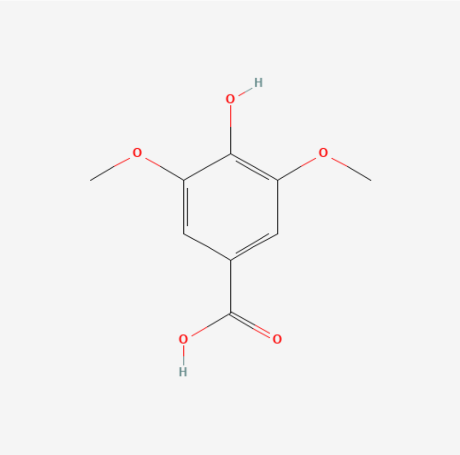

Syringic acid

Reishi (Ganoderma lucidum)

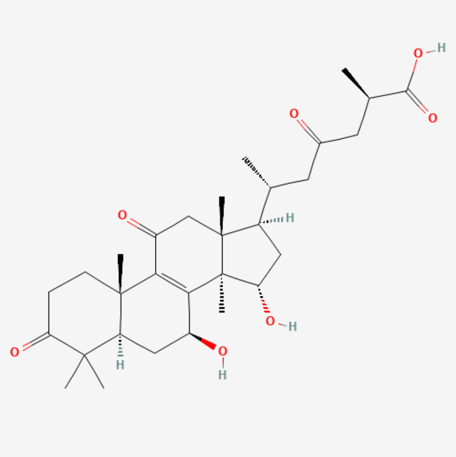

Ganoderic acid

Cordyceps sinensis

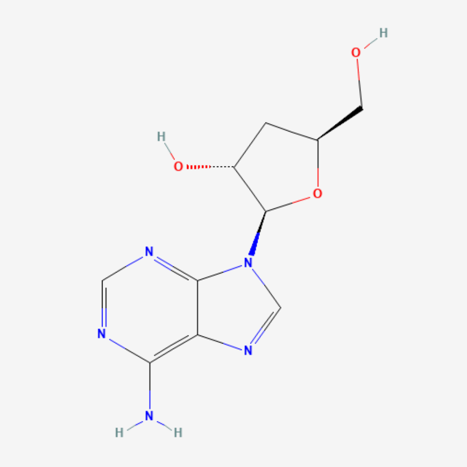

Cordycepin

Shiitake (Lentinula edodes)

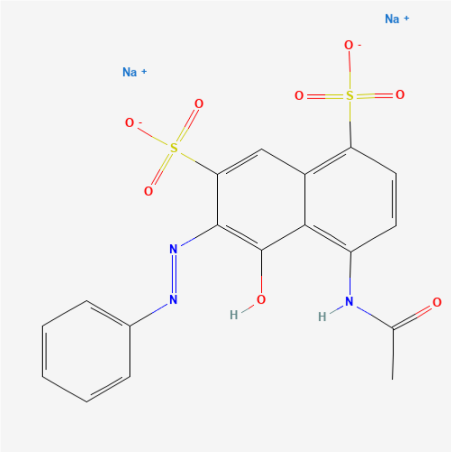

Lignin

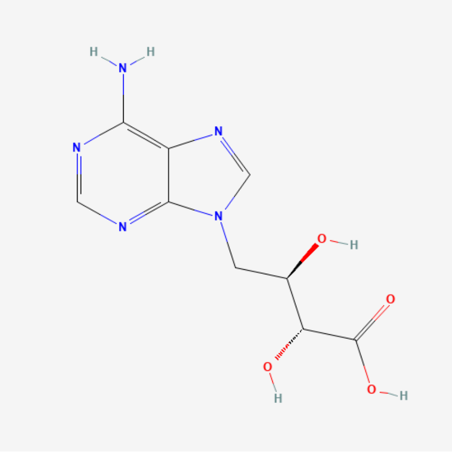

Erytadenine

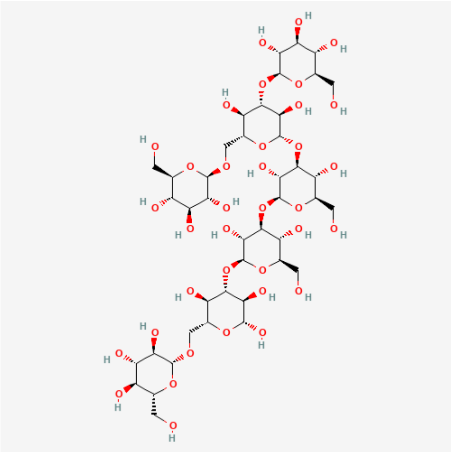

Lentinan
