# Supplementary material for: How Should the Worldwide Knowledge of Traditional Cancer Healing Be Integrated with Herbs and Mushrooms into Modern Molecular Pharmacology?
Source: Pharmaceuticals (Basel). 2022 Jul 14;15(7):868. doi: 10.3390/ph15070868 (PMC9320176; doi:10.3390/ph15070868)
Supplement: Supplementary file 1 [file pharmaceuticals-15-00868-s001.zip › Table S1.pdf]

Drug	Organism	Malignancies which are treated	Mechanism
**Bleomycin sulfate**	*Streptomyces verticillus*	Hodgkin lymphoma, Non-Hodgkin lymphoma, Squamous cell carcinoma of the penis, Squamous cell carcinoma of the cervix, Squamous cell carcinoma of the head and neck, Squamous cell carcinoma of the vulva, Testicular cancer, Malignant pleural effusion	Induced of DNA strand breaks
**Cabazitaxel**	Semi-synthetic derivative of a natural taxoids	Prostate cancer	Inhibitor of microtubule dymamics
**Dactinomycin**	*Streptomyces parvulus*	Ewing sarcoma Gestational trophoblastic disease Rhabdomyosarcoma Solid tumors Testicular cancer Wilms tumor	Inhibitor of transcription
**Daunorubicin hydrochloride**	*Streptomyces coeruleorubidus, Streptomyces peucetius*	Acute lymphoblastic leukemia Acute myeloid leukemia	Inhibitor of topoisomerase II
**Docetaxel**	Semi-synthetic analogue of paclitaxel (Taxol), which is derived from Pacific yew tree *Taxus brevifolia*	Breast cance,r Non-small cell lung cancer, Prostate cancer, Squamous cell carcinoma of the head and neck, Stomach adenocarcinoma and gastroesophageal junction adenocarcinoma	Inhibitor of microtubule dymamics
**Doxorubicin hydrochloride**	*Streptomyces coeruleorubidus* *Streptomyces peucetius*	Acute lymphoblastic leukemia (ALL), Acute myeloid leukemia (AML), Metastatic breast cancer, Gastric (stomach) cancer, Hodgkin lymphoma, Neuroblastoma, Non-Hodgkin lymphoma, Non-small cell lung cancer, Ovarian cancer, Small cell lung cancer, Soft tissue and bone sarcomas, Thyroid cancer, Bladder cancer, Kaposi's sarcoma, Wilms tumors, and others	Inhibits topoisomerase II
**Eribulin (Halaven)**	Fully synthetic macrocyclic ketone analogue of the marine natural product halichondrin B (*Halichondria okadai*)	Breast cancer Liposarcoma	Inhibitor of microtubule dynamics
**Etoposide**	Semisynthetic derivative of podophyllotoxin, a natural compound from root of *Podophyllum peltatum*	Small cell lung cancer, Testicular Cancer, Non-small Cell Lung Cancer, Hodgkin’s Disease, Acute Myeloid Leukemia, Acute Lymphocytic Leukemia, Wilms’ Tumor, Neuroblastoma, Kaposi’s Sarcoma	Inhibittor of topoisomerase II
**Everolimus**	*Streptomyces hygroscopicus*	Breast cancer,Pancreatic cancer,Ggastrointestinal cancer, Lung cancer, Renal cell carcinoma, Subependymal giant cell astrocytoma	Inhibitor of mTOR
**Idarubicin hydrochloride**	Semi-synthetic 4-demethoxy analogue of the antineoplastic anthracycline antibiotic daunorubicin	Acute myeloid leukemia (AML)	Inhibitor topoisomerase II,

**Irinotecan hydrochloride**	Semisynthetic derivative of natural compound camptothecin which is found in the Camptotheca acuminata	Colorectal cancer, Pancreatic cancer	Inhibitor topoisomerase I
**Ixabepilone**	Ixabepilone is a semi-synthetic analog of epothilone B, a natural chemical compound produced by *Sorangium cellulosum*	Breast cancer	Inhibitor of microtubule dymamics
**Leucovorin calcium (Folinic acid)**	Was discovered as growth factor required for the bacterium L*euconostoc citrovorum*	Colorectal cancer Anemia	Prevent harmful effects of methotrexate. It allows some purine/pyrimidine synthesis and normal DNA replication in normal cells in the presence of dihydrofolate reductase inhibitors
**Midostaurin**	Semi-synthetic derivative of staurosporine, an alkaloid from the *bacterium Streptomyces staurosporeus*	Acute myeloid leukemia (AML), Aggressive systemic mastocytosis with associated hematologic neoplasm mast cell leukemia	Protein kinase inhibitor
**Mitomycin**	*Streptomyces caespitosus*	Gastric (stomach) and pancreatic adenocarcinoma, Urothelial cancer	DNA crosslinker
**Omacetaxine mepesuccinate**	Semi-synthetic derivate of homoharringtonine (HHT) - a natural plant alkaloid derived from Cephalotaxus fortunei	Chronic myelogenous leukemia	Inhibitor of translation
**Paclitaxel**	*Pacific yew*	Various cancers	Inhibitor of microtubule dymamics
**Romidepsin**	Natural product obtained from the bacterium *Chromobacterium violaceum*	Cutaneous T-cell lymphoma	Inhibitor of histone deacetylases
**Asparaginase**	Enzyme from *Erwinia chrysanthemi*	Acute lymphoblastic leukemia	Depletion of asparagine amino acid
**Temsirolimus**	Temsirolimus is an ester derived from rapamycin - (Sirolimus) wich is a natural macrocyclic lactone produced by the bacterium *Streptomyces hygroscopicus*	Renal cell carcinoma	Inhibitor of mTOR
**topotecan hydrochloride**	Synthetic, water-soluble analog of the natural chemical compound camptothecin	Cervical cancer, Ovarian cancer, Small cell lung cancer	Inhibitor of topoisomerase I
**Tabectedin**	Semisynthetic antibiotic obtained from the bacterium *Pseudomonas fluorescens*	Liposarcoma and leiomyosarcoma	Blocks oncogenic transcription factor FUS-CHOP binding with DNA

**Valrubicin**	Semi-synthetic analog of the doxorubicin	Bladder cancer	Inhibitor of topoisomerase II.
**Vinblastine sulfate**	Madagascar periwinkle *Catharanthus roseus*	Breast cancer, Choriocarcinoma, Hodgkin lymphoma, Kaposi sarcoma, Non-Hodgkin lymphoma, Testicular germ cell tumors, other malignancies	Inhibitor of microtubule dymamics
**Vincristine sulfate**	Madagascar periwinkle *Catharanthus roseus*	Acute leukemia. Hodgkin lymphoma.<br>Neuroblastoma.<br>Non-Hodgkin lymphoma (NHL).<br>Rhabdomyosarcoma.<br>Wilms tumor.	Inhibitor of microtubule dymamics
**Vinorelbine tartrate**	Semi-ynthetic derivate of vinca alkaloids	Non-small cell lung cancer	Inhibitor of microtubule dymamics
