# Supplementary material for: How Should the Worldwide Knowledge of Traditional Cancer Healing Be Integrated with Herbs and Mushrooms into Modern Molecular Pharmacology?
Source: Pharmaceuticals (Basel). 2022 Jul 14;15(7):868. doi: 10.3390/ph15070868 (PMC9320176; doi:10.3390/ph15070868)
Supplement: Supplementary file 1 [file pharmaceuticals-15-00868-s001.zip › Table S2.pdf]

Combination chemotherapy regimens	Components
ABVD	Doxorubicin Hydrochloride (Adriamycin), Bleomycin, Vinblastine Sulfate, Dacarbazine
ABVE	Doxorubicin Hydrochloride (Adriamycin), Bleomycin, Vincristine Sulfate, Etoposide Phosphate
ABVE-PC	Doxorubicin Hydrochloride (Adriamycin), Bleomycin, Vincristine Sulfate, Etoposide Phosphate, Prednisone, Cyclophosphamide
AC	Doxorubicin Hydrochloride (Adriamycin), Cyclophosphamide
AC-T	Doxorubicin Hydrochloride (Adriamycin), Cyclophosphamide, Paclitaxel (Taxol)
ADE	Cytarabine (Ara-C), Daunorubicin Hydrochloride, Etoposide Phosphate
BEACOPP	Bleomycin Etoposide, Phosphate Doxorubicin Hydrochloride (Adriamycin), Cyclophosphamide, Vincristine Sulfate (Oncovin), Procarbazine Hydrochloride, Prednisone
BEP	Bleomycin, Etoposide Phosphate, Cisplatin
CAF	Cyclophosphamide, Doxorubicin Hydrochloride (Adriamycin), Fluorouracil
CEM	Carboplatin Etoposide Phosphate Melphalan Hydrochloride
CEV	Carboplatin, Etoposide Phosphate, Vincristine Sulfate
CHOP	Cyclophosphamide, Doxorubicin hydrochloride (Hydroxydaunorubicin), vincristine sulfate (Oncovin), Prednisone
COPDAC	Cyclophosphamide, Vincristine Sulfate (Oncovin), Prednisone, Dacarbazine
COPP	Cyclophosphamide, Vincristine Sulfate (Oncovin), Procarbazine Hydrochloride, Prednisone
COPP-ABV	Cyclophosphamide, Vincristine Sulfate (Oncovin), Procarbazine Hydrochloride, Prednisone, Bleomycin, Vinblastine Sulfate
CVP	Cyclophosphamide, Vincristine Sulfate, Prednisone
EPOCH	Etoposide Phosphate, Prednisone, Vincristine Sulfate (Oncovin), Cyclophosphamide, Doxorubicine Hydrochloride (Hydroxydaunomycin)
FOLFIRI	Leucovorin (Folinic Acid), Fluorouracil, Irinotecan Hydrochloride
FOLFIRI-BEVACIZUMAB	Leucovorin Calcium (Folinic Acid), Fluorouracil, Irinotecan Hydrochloride, Bevacizumab

FOLFIRI-CETUXIMAB	FOL– Leucovorin Calcium (Folinic Acid)<br>F– Fluorouracil<br>IRI– Irinotecan Hydrochloride<br>+ Cetuximab
FOLFIRINOX	FOL– Leucovorin Calcium (Folinic Acid)<br>F– Fluorouracil<br>IRIN– Irinotecan Hydrochloride<br>OX– Oxaliplatin
hyper-CVAD	C– Cyclophosphamide<br>V– Vincristine Sulfate<br>A– Doxorubicin Hydrochloride (Adriamycin)<br>D– Dexamethasone
ICE	I– Ifosfamide<br>C– Carboplatin<br>E– Etoposide Phosphate
JEB	J– Carboplatin (JM8)<br>E– Etoposide Phosphate<br>B– Bleomycin
MVAC	M– Methotrexate<br>V– Vinblastine Sulfate<br>A– Doxorubicin Hydrochloride (Adriamycin)<br>C– Cisplatin
OEPA	O– Vincristine Sulfate (Oncovin)<br>E– Etoposide Phosphate<br>P– Prednisone<br>A– Doxorubicin Hydrochloride (Adriamycin)
OPPA	O– Vincristine Sulfate (Oncovin)<br>P– Procarbazine Hydrochloride<br>P– Prednisone<br>A– Doxorubicin Hydrochloride (Adriamycin)
PAD	P– Bortezomib (PS-341)<br>A– Doxorubicin Hydrochloride (Adriamycin)<br>D– Dexamethasone
PCV	P– Procarbazine Hydrochloride<br>C– Lomustine (CCNU)<br>V– Vincristine Sulfate
PEB	P– Cisplatin (Platinol)<br>E– Etoposide Phosphate<br>B– Bleomycin
R-CHOP	R– Rituximab<br>C– Cyclophosphamide<br>H– Doxorubicin Hydrochloride (Hydroxydaunomycin)<br>O– Vincristine Sulfate (Oncovin)<br>P– Prednisone
R-CVP	R– Rituximab<br>C– Cyclophosphamide<br>V– Vincristine Sulfate<br>P– Prednisone

R-EPOCH	R Rituximab<br>E Etoposide Phosphate<br>P Prednisone<br>O Vincristine Sulfate (Oncovin)<br>C Cyclophosphamide<br>H Doxorubicin Hydrochloride (Hydroxydaunorubicin)
R-ICE	R Rituximab<br>I Ifosfamide<br>C Carboplatin<br>E Etoposide Phosphate
STANFORD V	Mechlorethamine Hydrochloride<br>Doxorubicin Hydrochloride<br>Vinblastine Sulfate<br>Vincristine Sulfate<br>Bleomycin<br>Etoposide Phosphate<br>Prednisone
TAC	T Docetaxel (Taxotere)<br>A Doxorubicin Hydrochloride (Adriamycin)<br>C Cyclophosphamide
VAC	V Vincristine Sulfate<br>A Dactinomycin (Actinomycin-D)<br>C Cyclophosphamide
VAMP	V Vincristine Sulfate<br>A Doxorubicin Hydrochloride (Adriamycin)<br>M Methotrexate<br>P Prednisone
VeIP	Ve Vinblastine Sulfate (Velban)<br>I Ifosfamide<br>P Cisplatin (Platinol)
VIP	V Etoposide (VP-16)<br>I Ifosfamide<br>P Cisplatin (Platinol)
XELIRI	XEL Capecitabine (Xeloda)<br>IRI Irinotecan Hydrochloride
